# Aggressive breast cancers secrete heme metabolites to alter macrophage immune suppression and function

**DOI:** 10.1038/s42003-026-10212-0

**Published:** 2026-05-09

**Authors:** Michelle M. Williams, Jessica L. Christenson, Nicole S. Spoelstra, Sabrina A. Hafeez, Li-Wei Kuo, Lyndsey S. Crump, Kathleen I. O’Neill, Alexis A. Hill, Angelica Phan, Lauren M. McCue, Thomas M. Hintelmann, Juanantonio Ruiz, Haley E. Sax, Jared Williams, Meher Preethi Boorgula, Andrew Goodspeed, Jill E. Slansky, Jennifer K. Richer

**Affiliations:** 1 Department of Pathology, University of Colorado Anschutz Medical Campus, Aurora, CO USA; 2 Department of Pharmacology and Chemical Biology, University of Pittsburgh, Pittsburgh, PA USA; 3 Cancer Biology Program, UPMC Hillman Cancer Center, Pittsburgh, PA USA; 4 University of Colorado Cancer Center, University of Colorado Anschutz Medical Campus, Aurora, CO USA; 5 Department of Biomedical Informatics, University of Colorado Anschutz Medical Campus, Aurora, CO USA; 6 Department of Immunology & Microbiology, University of Colorado Anschutz Medical Campus, Aurora, CO USA

**Keywords:** Breast cancer, Cancer microenvironment

## Abstract

The heme catabolism pathway is often elevated in aggressive cancers; however, the impact of this pathway and some of its byproducts on the tumor microenvironment remain largely unknown. In human breast cancers, tumor expression of the heme catabolizing enzyme heme oxygenase-1 (HO-1/*HMOX1*) is positively associated with macrophage abundance. In mouse mammary tumors, knockdown of *Hmox1* significantly decreased tumor growth and lung metastasis. Analysis of mammary tumor interstitial fluid compared to matching plasma revealed that the heme metabolite bilirubin was elevated intratumorally, which could be partially reversed via *Hmox1* knockdown. Further investigation revealed that bilirubin nearly ablates macrophage engulfment of dead tumor cells and significantly increases macrophage T cell suppression. Mammary tumors harboring *Hmox1* knockdown had a significant decrease in tumor growth rate and number of pro-metastatic CD206^+^ macrophages upon treatment with αPD-1. Depletion of intratumoral bilirubin levels impacts pro-tumor macrophage populations, particularly in combination with immunotherapy, demonstrating that heme catabolism and bilirubin act as immunomodulators in cancer.

## Abstract (web summary)

Insight into the underappreciated role of heme degradation and the heme metabolite bilirubin as an immunometabolite in aggressive breast cancers.

## Introduction

In 2019, the U.S. Food and Drug Administration approved the first checkpoint inhibitor in metastatic triple-negative breast cancer (TNBC), opening the door for immunotherapy use in breast cancer (BC).^1^ Despite initial excitement, checkpoint inhibitors have only been approved in select BC cases and for estrogen receptor-positive (ER^+^) BC, the most common BC subtype, immunotherapies remain in preclinical or clinical development. To reveal additional molecular drivers of BC progression that suppress anti-tumor immunity, our group and others manipulated an Epithelial-to-Mesenchymal Transition (EMT) program characteristically upregulated in TNBC compared to ER^+^ BC.^2–5^ Reversal of EMT in aggressive mesenchymal-like TNBC models limited immune suppression and identified metabolic pathways such as tryptophan and heme catabolism that may support tumor progression through production of immune modulatory metabolites.^2,6,7^

**Fig. 1:**
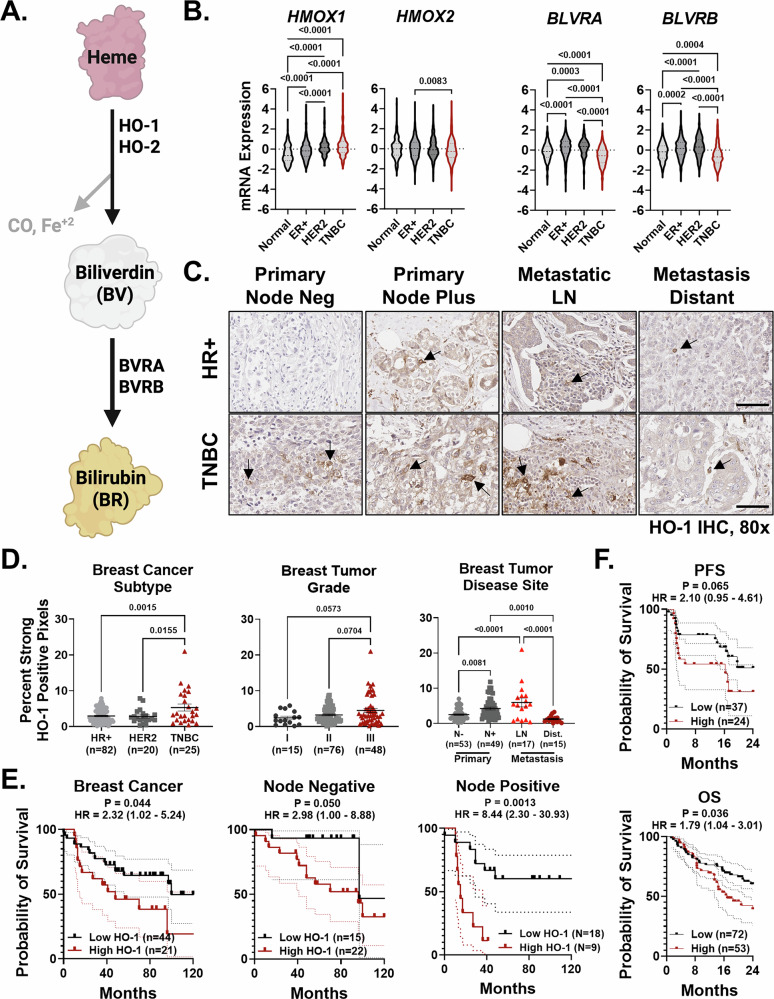
Heme oxygenase-1 expression is elevated in TNBC and predicts decreased survival. **A** Model of heme breakdown by heme oxygenase-1 (HO-1) and heme oxygenase-2 (HO-2) into carbon monoxide (CO), iron (Fe^+2^), and biliverdin (BV). BV is then converted into bilirubin (BR) by biliverdin reductase-A (BLVRA) or biliverdin reductase-B (BLVRB). Created in BioRender. Williams, M. (2026) https://BioRender.com/ua57hd3**B**. Expression of heme breakdown enzymes was observed using CBioPortal. Shown is a violin plot of the Log2 transformed z-score for each gene compared to the expression distribution of all samples (normal n = 140, ER^+^ n = 1140, HER-enriched n = 220, TNBC n = 398, one-way ANOVA with Tukey’s multiple comparison test). HO-1 levels were assessed in a tissue microarray via IHC. Shown are representative images (**C**, scale bar = 60 microns) and quantification of the average percent medium (2+) and strong (3+) positive pixels determined with ImageScope software for 1-4 tissue cores per specimen for primary node negative (N-), primary node positive (N+), local lymph node metastases (LN), and distant metastases (Dist.) (**D**, mean ± SEM, one-way ANOVA with Tukey’s multiple comparison test). Examples of HO-1 positive cells are denoted with arrows. **E** Overall survival of breast cancer patients was assessed using Kaplan-Meier Plotter and the best cutoff for HO-1 protein expression (p-value log-rank/Mantel-Cox test and hazard ratio/HR Mantel-Haenszel test). The same analysis was completed after patients were stratified by lymph node involvement. **F** Progression-free (PFS, n = 61) and overall survival (OS, n = 125) of cancer patients assessed using Kaplan-Meier Plotter and the best cutoff for the mean expression of *HMOX1*, *HMOX2*, *BLVRA*, and *BLVRB* (p-value log-rank/Mantel-Cox test and hazard ratio/HR Mantel-Haenszel test).

The enzymes heme oxygenase-1 (HO-1/*HMOX1*) and heme oxygenase-2 (HO-2/*HMOX2*) catalyze the rate limiting step of heme degradation, catabolizing heme into carbon monoxide (CO), iron (Fe^+2^), and biliverdin (Fig. 1A). Biliverdin is then rapidly converted to bilirubin by biliverdin reductase-A (BLVRA) or biliverdin reductase-B (BLVRB). Enzymes in this pathway have been implicated in breast tumor progression and therapy resistance. For example, genetic or pharmacologic inhibition of BLVRA in human ER^+^ or TNBC models enhanced cell death and reduced expression of EMT transcription factors.^8,9^ High *HMOX1* correlates with a shorter relapse free survival in BC patients,^10,11^ possibly due to the impact of HO-1 on tumor cell extravasation and metastatic colonization.^11,12^ Furthermore, treatment with HO-1 inhibitors alone or in combination with chemo- or immunotherapy decreased tumor growth in BC, melanoma, and lung cancer models by impacting immune cells in the tumor microenvironment (TME).^13–17^ However, these works do not interrogate heme catabolism as a mediator of communication between tumor cells and the TME.

Heme metabolites, CO, biliverdin, and bilirubin, have established antioxidant and pro-tumor properties. For instance, CO produced by HO-1 enzymatic activity supports tumor cell transendothelial migration.^12^ Meanwhile, Fe^+2^ is an established pro-oxidant that can induce ferroptosis, or cell death resulting from excess iron.^18^ Interestingly, a recent report showed that HO-1-dependent modulation of intracellular heme and iron pools serves as a rheostat for TNBC migration.^19^ These examples demonstrate that when tumor-HO-1 is upregulated it can support disease progression, in part through increased production and secretion of HO-1 metabolites. However, the impact of these metabolites, especially biliverdin and bilirubin, on the TME remains understudied. Evidence from cardiac allografts demonstrated that biliverdin reduces T cell infiltration and activation by suppressing IL-2 production^20^ and inflammatory TLR4 signaling in macrophages.^21^ In severe cases of jaundice, autoimmune diseases, or in organ transplant models, bilirubin limited B cell, T cell, and macrophage infiltration and function.^22–27^ Even though serum bilirubin levels are elevated in patients with metastatic versus early stage disease, and they predicted poor BC outcomes,^28–31^ bilirubin has never been studied as an immune modulator in cancer. Further insight into the effects of heme catabolism on BC progression, including the impact of bilirubin on the TME, is needed. Herein, we focused on the impact of bilirubin on macrophages and showed that TNBC heme catabolism supports a pro-tumor immune microenvironment.

## Results

### Heme oxygenase-1 is elevated in TNBC and predicts decreased survival

To interrogate the role of heme catabolism in BC progression, publicly available datasets were utilized to observe gene expression of pathway enzymes in various BC subtypes compared to normal breast tissue.^32–34^ Expression of *HMOX1*, *BLVRA*, and *BLVRB* were upregulated in ER^+^ and HER2-enriched BC specimens when compared to normal breast (Fig. 1B). *HMOX1* was also significantly upregulated in TNBC specimens. However, expression of *BLVRA* and *BLVRB* was downregulated in TNBC when compared to normal breast specimens. In contrast, *HMOX2* did not significantly change in any subtype when compared to normal tissues, consistent with the fact that HO-2 is constitutively active and not stimulated by factors commonly seen in the TME, such as inflammation and hypoxia.^35^ Overall, these results suggest that BC relies on activation of the heme breakdown pathway.

We observed expression of HO-1 by immunohistochemistry (IHC) in a tissue microarray (TMA) containing specimens from various BC subtypes throughout tumor progression. First, HO-1 levels were quantified using ImageScope software to detect percent weak (1+), medium (2+), and strong (3+) positive pixels in control tissues: myometrium, placenta, liver, and spleen (Supplementary Fig 1A). As anticipated, spleen had the highest percent HO-1 positivity, followed by the liver, placenta, and myometrium (Supplementary Fig. 1B). Medium and strong HO-1 staining was significantly increased in TNBC specimens when compared to those from the other BC subtypes (Fig. 1C, D). HO-1 levels were also increased in grade III compared to grade I or II tumors when analyzing specimens from all BC subtypes (Fig. 1C, D). When observing HO-1 expression in all BC subtypes across disease progression, HO-1 was significantly elevated in breast tumors with lymph node (LN) involvement compared to those without. HO-1 was most highly expressed in LN metastases. When distant metastases from all BC subtypes were stratified by site, HO-1 expression was elevated in the brain, lung, and liver when compared to bone (Supplementary Fig 1C). However, this finding did not reach statistical significance, likely due to sample size. HO-1 did not significantly correlate with other known risk factors such as histologic type or race (Supplementary Fig. 1C). Finally, we explored whether HO-1 predicts differences in survival using KM Plotter^36^ and found that in all BC subtypes high HO-1 protein expression predicted decreased overall survival when compared to low HO-1 (Fig. 1E). Although this cohort size did not allow us to assess whether HO-1 is more predictive in TNBC versus other BC subtypes, the difference in OS was particularly striking in the cohort with LN positive disease, suggesting that HO-1 may support disease progression. Finally, we assessed the impact of heme catabolism on immunotherapy response as recent reports link this pathway to immunotherapy resistance.^15,16^ High expression of heme catabolism genes (*HMOX1, HMOX2, BLVRA*, and *BLVRB*) during immunotherapy treatment predicted a decrease in progression-free and overall survival in a cohort of patients representing diverse cancer types (bladder, esophageal, adenocarcinoma, glioblastoma, hepatocellular cancer, head and neck squamous cell carcinoma, non-small cell lung cancer, melanoma, and urothelial cancer, Fig. 1F).

### Macrophage density is associated with breast tumor-HO-1

**Fig. 2:**
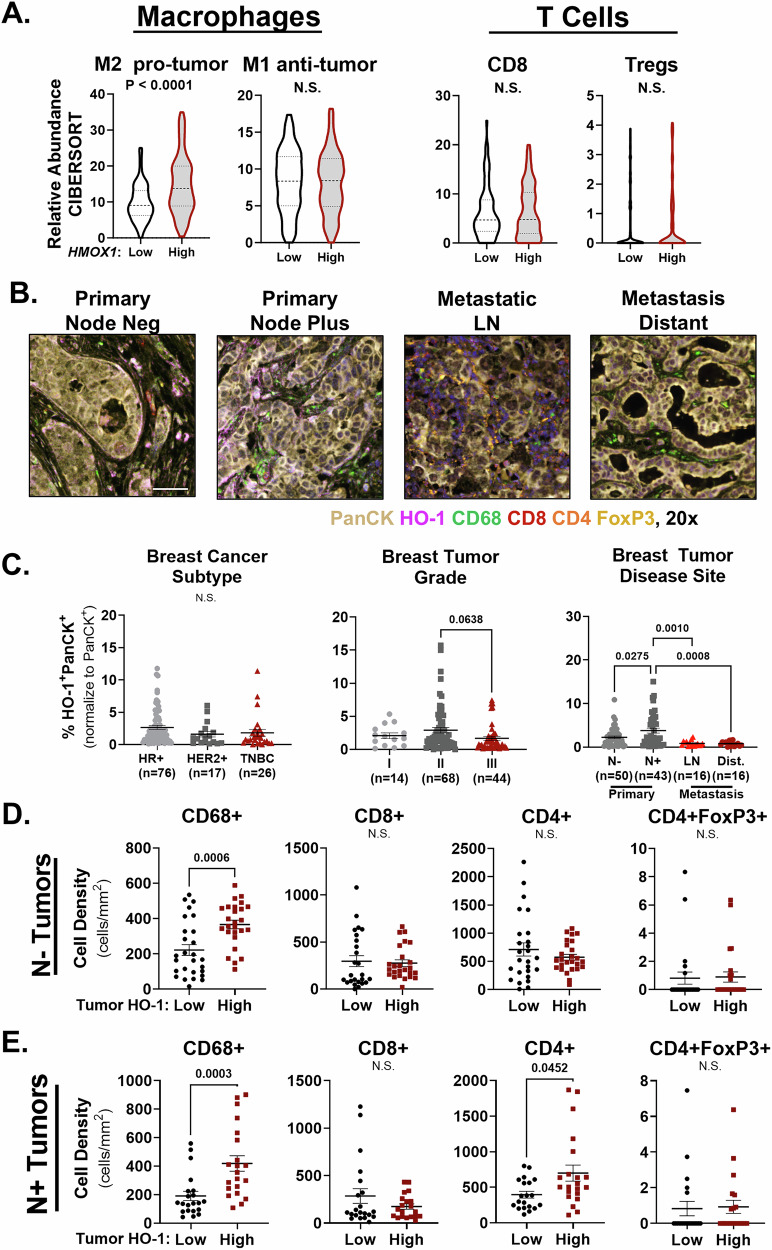
Macrophage density is associated with breast tumor-HO-1. **A** Bulk mRNA sequencing data of human breast cancer specimens was accessed via CBioPortal and analyzed using CIBERSORT. Shown is a violin plot for the predicted relative abundance of macrophage and T cell populations in TNBC specimens after stratification by *HMOX1* expression (low signifies *HMOX1* in the bottom quartile and high signifies *HMOX1* in the upper quartile, n = 101 per group, unpaired two-tailed T test). HO-1 and immune cell markers were observed in a tissue microarray using a custom multispectral fluorescence panel (PanCK/tan = tumor cell, HO-1/magenta, CD68/green = macrophage, CD8/red = cytotoxic T cell, CD4/orange = helper T cell, FoxP3/yellow = regulatory T cell). Shown are representative images (**B**, 20×, scale bar = 50 microns) and quantification for the percent HO-1^+^ tumor cells (HO-1^+^PanCK^+^) relative to the total number of tumor cells (**C**, mean ± SEM, one-way ANOVA with Tukey’s multiple comparison test). The density of immune cells in primary node negative (N-) (**D**, n = 24-27 per group) and primary node positive (N+) (**E**, n = 22 per group) tissues is shown after stratification by median tumor-HO-1 expression (mean ± SEM, unpaired two-tailed T test).

Since heme metabolites are known to be immune modulatory, we conducted a CIBERSORT analysis to predict relative immune cell abundance in TNBC specimens.^37^ Specimens were stratified based on mRNA expression of *HMOX1* in the top or bottom quartile (Fig. 2A). High expression of *HMOX1* predicted a striking increase in the number of M2-like pro-tumor macrophages when compared to tumors with low *HMOX1* (Fig. 2A). However, there was no predicted change in M1 macrophages, cytotoxic CD8^+^ T cells, or suppressive T regulatory cells (Tregs) between groups. Interestingly, B cell and follicular helper T cell populations were lower in *HMOX1* high versus low tumors, suggesting a possible decrease in tertiary lymphoid structures in HO-1 high tumors (Supplementary Fig 2A). Changes in T cell and macrophage populations were not predicted in normal breast specimens but were present in the other BC subtypes (Supplementary Fig 2B). Overall, this data demonstrates that HO-1^+^ BC, particularly TNBC, harbor a TME with abundant suppressive macrophages.

Since this CIBERSORT analysis was conducted on bulk mRNA and the IHC results in Fig. 1 suggest that HO-1 is expressed by both tumor and immune cells, we further explored tumor-specific HO-1 expression using multispectral fluorescence (mIF). A panel was designed to quantify the number of HO-1^+^ tumor cells (PanCK^+^HO-1^+^) and the presence of cytotoxic T cells (CD8^+^), helper T cells (CD4^+^FoxP3^-^), Tregs (CD4^+^FoxP3^+^), and macrophages (CD68). We stained the same TMA used in Fig. 1 and analyzed the percent tumor cells positive for HO-1, which revealed no significant change with BC subtype or tumor grade (Fig. 2B, C). However, tumor-HO-1 was significantly increased in breast tumors from patients with LN involvement when compared to those without. Since these results were disparate from the HO-1 IHC analysis, we observed what immune cell populations expressed HO-1. HO-1 was detected in macrophages, CD4^+^ T cells, and CD8^+^ T cells (Supplementary Fig 3A). These findings suggest that HO-1 levels by IHC were highest in LN metastases due to an enrichment of HO-1^+^ immune cells in these specimens.

To confirm our CIBERSORT analysis, we investigated whether tumor-HO-1 levels predicted changes in immune cell abundance. In node negative and positive disease, specimens that had high tumor-HO-1 had a significant increase in macrophage density when compared to specimens with low tumor-HO-1 (Fig. 2D, E). However, there was no significant change in other immune cell populations except for CD4^+^ T cells that increased with tumor-HO-1 in LN involved tumors. In LN or distant metastasis specimens, tumor-HO-1 was not associated with differential macrophage density (Supplementary Fig 3B, C). Together these results suggest that breast tumors with high HO-1 have increased macrophage abundance.

### HO-1 supports tumor growth and metastasis

**Fig. 3:**
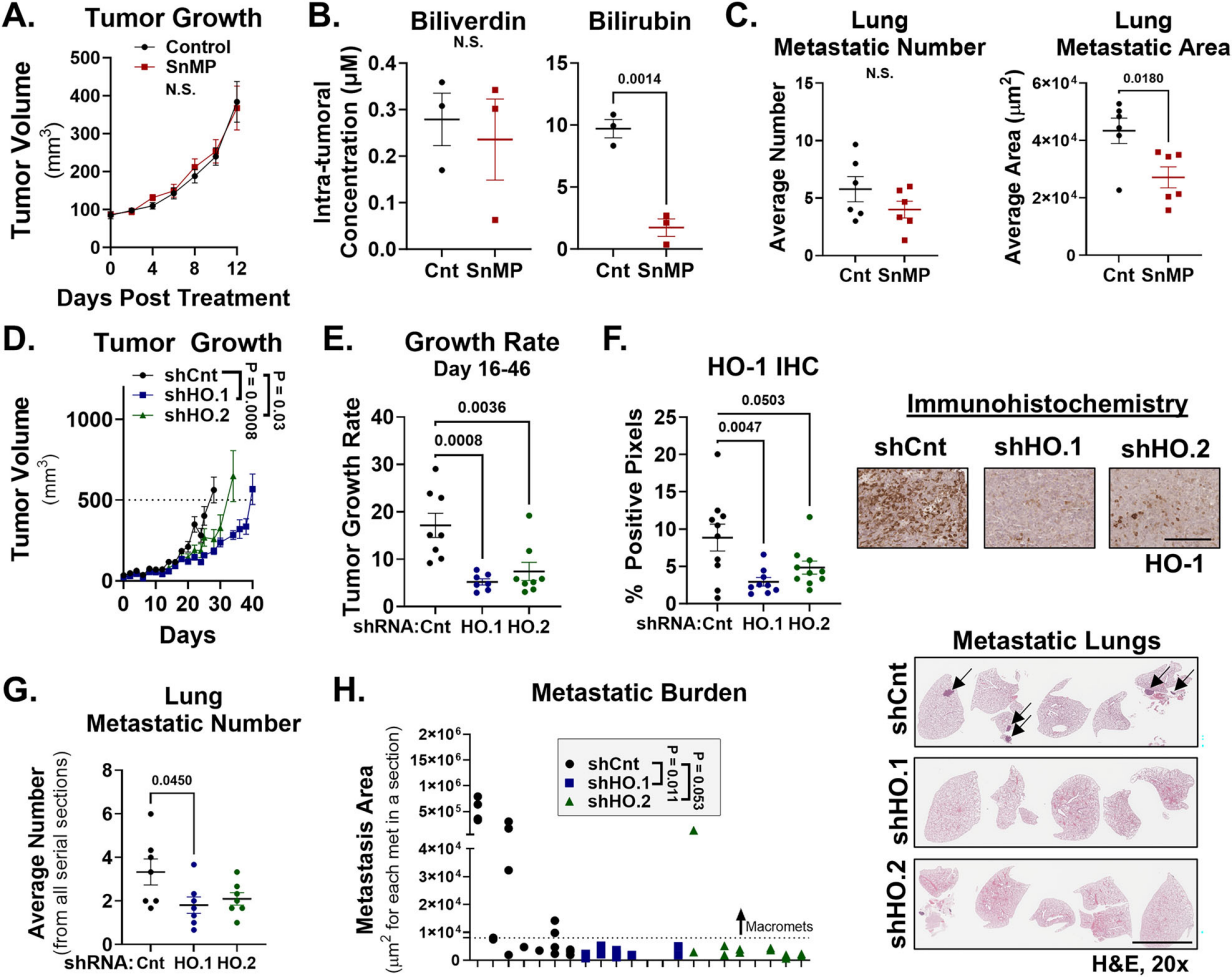
HO-1 supports tumor progression to metastasis. 66Cl-4 mammary carcinoma cells were injected unilaterally into the mammary fat pads of syngeneic BALB/CJ mice (n = 10 per group, total mice n = 20). When tumors reach an average of 80 mm^3^ animals were randomized to treatment with SnMP (25 mg/kg, daily oral gavage) or vehicle control. Shown is the mean ± SEM for tumor volume (**A**). Tumoral levels of biliverdin and bilirubin were determined in whole tumor lysates at endpoint via mass spectrometry (**B**, n = 3 per group, mean ± SEM, unpaired two-tailed T test). Metastasis was also assessed via H&E staining on three serial sections per lung using ImageScope Software (**C**). Shown is the average number of metastases and metastatic area per mouse (n = 6 per group, mean ± SEM, unpaired two-tailed T test). Similar experiments were conducted with 66Cl-4 cells harboring scramble control shRNA (shCnt) or two separate shRNA sequences against HO-1 (shHO.1 and shHO.2, n = 8 per group, total mice n = 24). Depicted is the tumor volume (**D**, mean ± SEM, one-way ANOVA with Dunnett’s multiplex comparison test on area under the curve for each tumor) and growth rate from day 16 to 46 of each tumor (**E**, mean ± SEM, one-way ANOVA with Dunnett’s multiple comparison test). **F** IHC staining for HO-1 was conducted in tumors from **D**. The percent HO-1 positive pixels and representative images are shown (**F**, n = 8–10 per group, scale bar = 200 microns, mean ± SEM, one-way ANOVA with Dunnett’s multiple comparison test). Metastatic number was observed via H&E staining on three serial sections (**G**, mean ± SEM, one-way ANOVA with Dunnett’s multiple comparison test). Also shown are the area of each detected metastasis per mouse (**H**, one-way ANOVA with Dunnett’s multiple comparison test of the average metastatic area per mouse) and representative images (arrows denote metastases, scale bar = 7 mm).

To test the impact of HO-1 on tumor progression, we utilized the 66Cl-4 mammary carcinoma cell line that metastasizes to the lung from the orthotopic site (mammary fat pad). This model was selected because 66Cl-4 cells express HO-1 and previous studies assessing the impact of HO-1 inhibitors on breast tumor progression used mammary carcinoma models that have little to no HO-1 expression in the tumor compartment (Supplementary Fig 4A).^14^ 66Cl-4 cells were introduced into the mammary fat pads of syngeneic female BALB/CJ mice. After tumors were established, mice were treated daily by oral gavage with tin mesoporphyrin (SnMP). SnMP was selected for this study because it is a potent HO-1/HO-2 competitive inhibitor that is used to treat severe cases of jaundice in neonates.^38–40^ SnMP treatment did not impact tumor growth when compared to vehicle control (Fig. 3A). Although SnMP did not decrease tumor biliverdin levels (Fig. 3B), it did significantly deplete levels of bilirubin by 4-fold when compared to vehicle, suggesting the inhibitor effectively limited HO-1/HO-2 enzymatic activity. To assess metastasis, lungs were serially sectioned and three sections per lung were stained with hematoxylin and eosin (H&E). ImageScope Software was utilized to measure the number of metastases and the area of each metastasis. SnMP treatment had a non-significant impact on metastatic number (Fig. 3C); however, there was a significant decrease in the average metastatic area per lung with SnMP treatment compared to vehicle control. Thus, while HO-1 pharmacologic inhibition did not impact tumor growth it modestly affected metastasis, a result that was also seen in the 4T1 model.^12^

Since HO-1 is expressed by tumor and immune cells in human BC, we tested the effects of tumor-specific HO-1 inhibition by knocking down *Hmox1* in 66Cl-4 mammary carcinoma cells using three distinct shRNA sequences (66Cl-4 shHO.1, 66Cl-4 shHO.2, and 66Cl-4 shHO.3). Western blot analysis confirmed that HO-1 expression was decreased by 70% or more in each knockdown line when compared to a non-targeting control (66Cl-4 shCnt, Supplementary Fig 4B). When grown in culture, *Hmox1* knockdown significantly decreased tumor cell proliferation and soft agar growth in two of three knockdown lines when compared to 66Cl-4 shCnt cells; however, soft agar colony size was not impacted by *Hmox1* knockdown (Supplementary Fig 4C, D). After orthotopic injection into female BALB/CJ mice, 66Cl-4 shHO.1 and shHO.2 tumors had delayed tumor growth and a significant decrease in tumor growth rate when compared to shCnt counterparts (Fig. 3D, E). To confirm that HO-1 depletion was maintained at endpoint, we formalin fixed and paraffin embedded (FFPE) tumors for IHC analysis. HO-1 levels were decreased in knockdown compared to control tumors at endpoint (Fig. 3F). HO-1 inhibition significantly decreased the number of lung metastases (Fig. 3G). We also assessed metastatic burden by quantifying the area of each metastasis. While metastases from shCnt tumors outgrew into large macrometastases, those from *Hmox1* knockdown tumors remained small (Fig. 3H), demonstrating that tumor-HO-1 may support tumor growth and lung metastasis.

We examined the histology of tumors via H&E analysis to gain insight into the effects of *Hmox1* knockdown on tumor progression and noted morphological changes in HO-1-depleted tumors (Supplementary Fig. 5A). For example, *Hmox1* knockdown tumors had more small round cells resembling immune cells. We also quantified expression of cell death (cleaved caspase-3) and proliferation (Ki67) markers. Cell turnover (cleaved caspase-3 divided by Ki67) did not change between experimental groups (Supplementary Fig. 5B), demonstrating that HO-1 inhibition did not directly alter these tumor intrinsic properties. To test whether *Hmox1* knockdown impacts progression via modulation of immune cells in the TME, 66Cl-4 shCnt and shHO.1 lines were introduced into female BALB/CJ RAG2 KO/IL2Rgc KO/SIRPa(NOD) mice that lack functioning T, B, and NK cells. Unlike in immune competent mice, tumor growth and metastatic burden were not significantly different between groups (Supplementary Fig. 5C, D). Together these results suggest that tumor-HO-1 impacts progression in part via effects on the TME.

### Tumor-secreted bilirubin alters macrophage function

**Fig. 4:**
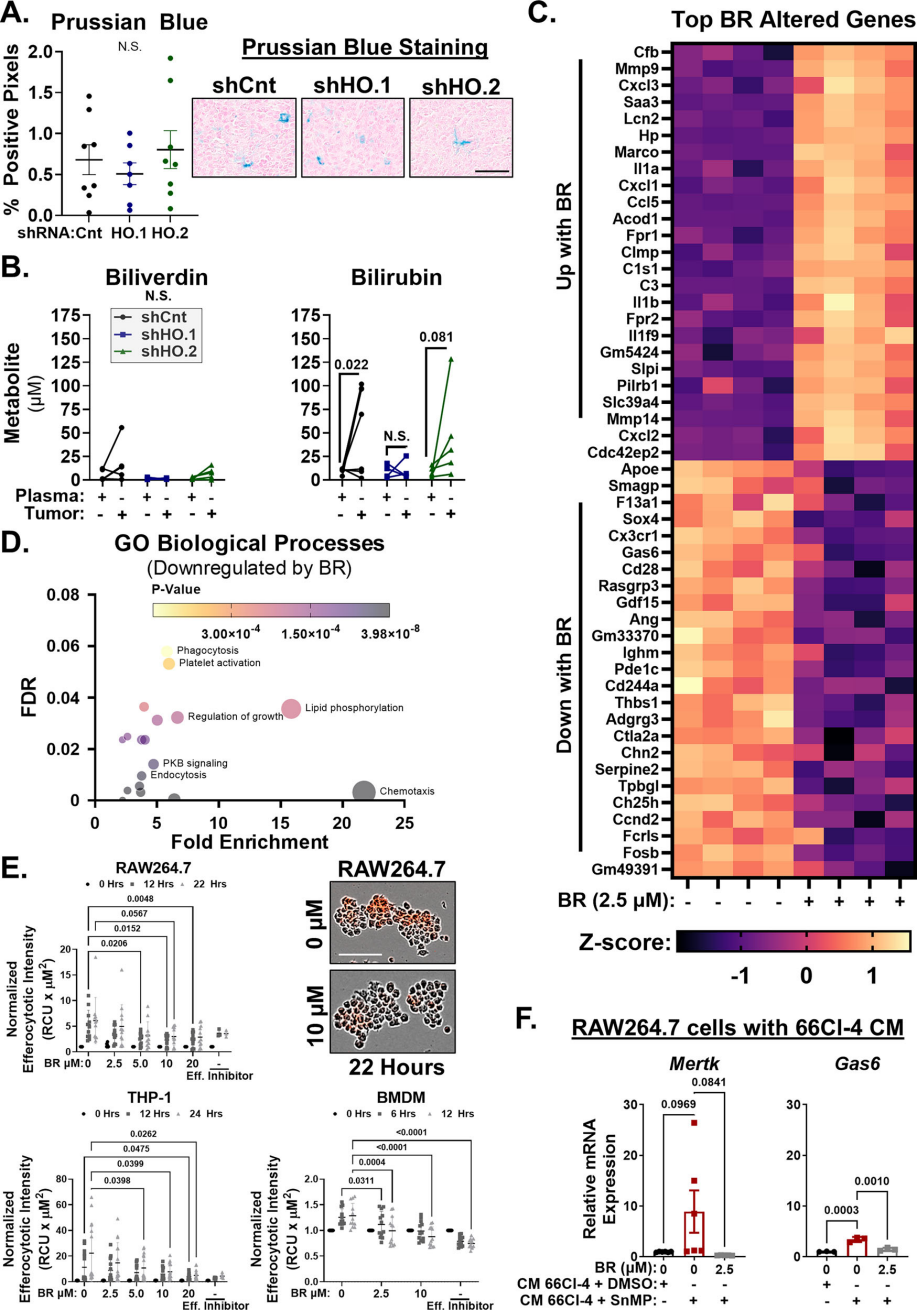
Tumor secreted bilirubin alters macrophage function. **A** Free iron was assessed in 66Cl-4 shCnt, shHO.1, and shHO.2 mammary tumors at endpoint using Prussian blue staining. Shown is the percent positive pixels and representative images (scale bar = 50 microns, n = 7–8 per group, mean ± SEM, one-way ANOVA with Dunnett’s multiple comparison test). **B** 66Cl-4 shCnt, shHO.1, and shHO.2 mammary tumors were centrifuged to collect tumor interstitial fluid (TIF) that was analyzed with matching plasma by mass spectrometry to detect levels of biliverdin and bilirubin (n = 4–5 per group, paired two-tailed T test). **C** BMDM harvested from 4 separate mice were treated with 2.5 μM bilirubin (BR) or DMSO control for 48 h then mRNA was extracted and sequenced. Shown is a heatmap of the Z-score normalized gene expression of the top 25 significantly upregulated and downregulated genes with bilirubin treatment (FDR < 0.05). **D** NIH DAVID was utilized to assess the ontology of genes downregulated by bilirubin treatment in BMDM. Shown is the predicted fold enrichment in each biological process for the top 10 enriched processes with a *p*-value < 0.0005. **E** Macrophages were cultured with dead tumor cells (66Cl-4 for RAW264.7 and BMDM or BT549 for THP-1) dyed with a fluorescent marker that emits a bright red light in the high pH of the lysosome. 0-10 μM BR or the efferocytosis inhibitor (UNC2025, 50 nM) were added at the same time as dead cells. Efferocytic intensity (red signal per μM^2^) was observed every 30 min for up to 24 h using the IncuCyte Zoom imaging system (n = 12-15, mean ± SD, two-way ANOVA with Dunnett’s multiple comparison test, scale bar = 75 microns). **F** Efferocytosis genes (*MertK* and *Gas6*) were observed via qRT-PCR in RAW264.7 cells after 48 h treatment with conditioned medium (CM) from 66Cl-4 cells with and without HO-1 inhibition (10 µM SnMP) and 2.5 µM bilirubin treatment (n = 3–6, mean ± SD, one-way ANOVA with Tukey’s multiple comparison test).

Since tumor-HO-1 may impact the TME via its metabolites, we observed the levels of free iron in 66Cl-4 mammary tumors with and without *Hmox1* knockdown using Prussian Blue staining (Fig. 4A). Analysis of percent Prussian Blue positive pixels revealed no significant change between groups. In a separate experiment, we collected and measured biliverdin and bilirubin levels in matching tumor interstitial fluid (TIF) and plasma from mice harboring 66Cl-4 shCnt, shHO.1, or shHO.2 tumors (Supplementary Fig 6A, B). Although biliverdin was expressed at low levels in many of the specimens tested, bilirubin was significantly enriched in TIF of shCnt tumors when compared to matching plasma from the same mice (Fig. 4B). However, in mice harboring shHO.1 or shHO.2 tumors, bilirubin levels were not significantly different in TIF compared to matching plasma. These results suggest that mammary tumors have a unique bilirubin-rich microenvironment that can be reversed by tumor-HO-1 inhibition.

To determine if bilirubin alters immune cell function, we tested the impact of exogenous bilirubin treatment on macrophages. We focused on macrophages because they are enriched in HO-1^+^ BC specimens. Primary bone marrow cells were isolated from 4 different 8-week-old BALB/CJ mice and differentiated into macrophages (bone marrow-derived or BMDM). BMDM were then treated with 2.5 μM unconjugated bilirubin or vehicle control for 48 h. After treatment, mRNA harvested using TriZol was sequenced using the NOVASEQ 600 platform. The top 25 upregulated and downregulated genes were presented in a heatmap (Fig. 4C and Supplementary Table 1). Bilirubin treatment significantly increased expression of genes that encode inflammatory cytokines such as (C-X-X motif) ligand 1 (*Cxcl1*), interleukin-36 gamma (*Il1f9*), and interleukin-1 alpha and beta (*Il1α* and *Il1β*). Also elevated with bilirubin treatment were immune suppressive scavengers such as haptoglobin (*Hp*) and *Marco*^41,42^ and pro-metastatic factors such as matrix metalloprotease-9 (*Mmp9*), *Mmp14*, and C-C motif chemokine ligand 5 (*Ccl5*).^43^ Meanwhile, bilirubin significantly decreased genes that regulate macrophage physiologic function such as apolipoprotein E (*Apoe*) and SLAMF4 (*Cd244a*) that affect phagocytosis,^44,45^ and growth arrest specific-6 (*Gas6*) and cholesterol 25-hydrolase (*Ch25h*), which regulate dead cell engulfment or efferocytosis.^46^ This gene signature suggests that bilirubin supports macrophage immune suppression and dysfunction.

NIH DAVID was conducted on top differentially expressed genes (Supplementary Table 2) to observe predicted changes in Gene Ontology Biological Processes (GO BP). GO BP downregulated by bilirubin included “Phagocytosis“ and “Endocytosis” (Fig. 4D and Supplementary Table 3), while those upregulated by bilirubin included “Immune Response”, “Cell Adhesion”, and “Bone Remodeling” (Supplementary Fig 7A and Supplementary Table 4). Gene Set Enrichment Analysis (GSEA) revealed the top Hallmark Gene Sets upregulated by bilirubin were ‘Interferon Gamma’, ‘Inflammatory’, and “IL-6-Jak-Stat3” (Supplementary Table 5). Finally, we utilized a program to predict which upstream transcriptional regulators may account for differential gene expression after bilirubin treatment. The top 50 hits were involved in the NFκB signaling pathway (Supplementary Table 6), suggesting that bilirubin may impact macrophage function by modulating NFκB activity. We confirmed this finding using RAW264.7 cells treated with 10 µM bilirubin for 0–48 h. Bilirubin treatment stimulated phosphorylation of NFκB p65 at each timepoint (Supplementary Fig. 7B). Further, 48 h bilirubin treatment significantly increased IL-6 secretion by RAW264.7 cells. These preliminary studies suggest that bilirubin may rewire macrophage signaling pathways by inducing the NFκB pathway.

Based on our mRNAseq results, we were interested in testing the impact of bilirubin on macrophage function. We decided to focus on efferocytosis because expression of the efferocytosis receptors (*Mertk* and *Axl*) and ligands (*Gas6* and *Pros1*) was downregulated upon bilirubin treatment (Supplementary Fig. 7C). We confirmed these results via qRT-PCR in three macrophage models: the RAW264.7 cell line, additional primary BMDM, and human THP-1 monocyte-derived macrophages. Although there was heterogeneity between models, mRNA expression of the efferocytosis receptor *Mertk* and/or the ligand *Gas6* was decreased in each model with 2.5 or 10 µM bilirubin (Supplementary Fig. 7D). Efferocytosis is a physiologic function that can be triggered in macrophages by high levels of cell death resulting from mammary gland/breast involution or treatment with cytotoxic cancer therapies such as chemotherapy and radiation. Thus, we further assessed the impact of bilirubin on macrophage efferocytosis by culturing RAW264.7, BMDM, and THP-1 cells with dead tumor cells. Prior to co-culture, dead cells were dyed with pHrodo, a fluorescent marker that emits a bright red signal in the high pH of macrophage lysosomes. Red signal was then tracked over time using the IncuCyte imaging system, which revealed that bilirubin significantly decreased red signal intensity, indicative of macrophage efferocytosis in each of the models tested (Fig. 4E). Remarkably, in the RAW264.7 model, bilirubin limited efferocytosis to a greater extent than the positive control UNC2025, a MerTK/FLT3 inhibitor that has been shown to block efferocytosis.^47^ Next, we assessed the impact of bilirubin-induced NFκB signaling on efferocytosis conducting a similar assay in the presence and absence of an NFκB inhibitor (Supplementary Fig. 7E). Blockade of NFκB signaling rescued efferocytosis when used in combination with physiologic bilirubin concentrations. Thus, bilirubin may support a dysfunctional macrophage population that cannot engulf and clear dead cells via NFκB signaling.

To test the impact of tumor-secreted bilirubin on macrophages, we conducted conditioned medium (CM) rescue experiments. RAW264.7 macrophages were cultured with CM from 66Cl-4 cells treated with DMSO control or 10 µM SnMP, a dose that limits HO enzymatic activity and bilirubin secretion but does not limit tumor cell proliferation (Supplementary Fig. 8A, B). Culturing RAW264.7 macrophages with CM from HO inhibited mammary carcinoma cells increased *Mertk* and *Gas6* (Fig. 4F). Interestingly, when bilirubin concentrations were restored in SnMP CM by supplementing with 2.5 μM bilirubin, gene levels of *Mertk* and *Gas6* were rescued, suggesting their expression may be regulated by tumor-produced bilirubin. Similar findings were seen in THP-1 macrophage model treated with CM from BT549 cells, and RAW264.7 macrophages treated with CM from *Hmox1* knockdown cells (Supplementary Fig. 8C–G). These results suggest that tumor-HO-1 may impact the function of macrophages in the TME via its secreted metabolite bilirubin.

### Bilirubin enhances macrophage immune suppression

We tested the effects of bilirubin on phenotypes that are more characteristic of tumor-associated macrophages (TAMs). To start, we conducted flow cytometry on BMDM treated with bilirubin for 48 h using the anti-tumor macrophage marker iNOS, pro-tumor macrophage marker Arg1, and the efferocytosis marker MerTK (gating strategy in Supplementary Fig. 9A). Bilirubin treatment significantly increased the number of live macrophages (F4/80^+^CD11b^+^), but this was not the case with control treatments used to induce anti-tumor (lipopolysaccharide/LPS and interferon-gamma/IFNγ) or pro-tumor (interleukin-4/IL-4 and IL-13) macrophage populations (Supplementary Fig. 9B). Interestingly, bilirubin did not impact the percent macrophages expressing Arg1 but it did increase the percent iNOS^+^ macrophages (Supplementary Fig. 9C, D). There was a modest but significant increase in the percent MerTK-positive BMDM and the mean fluorescence intensity (MFI) of MerTK in BMDM treated with bilirubin (Supplementary Fig. 9E). These findings are consistent with our transcriptional data that revealed that bilirubin induces an NFκB transcriptional program in macrophages, which has been shown by others to support macrophage survival and iNOS expression.^48,49^

**Fig. 5:**
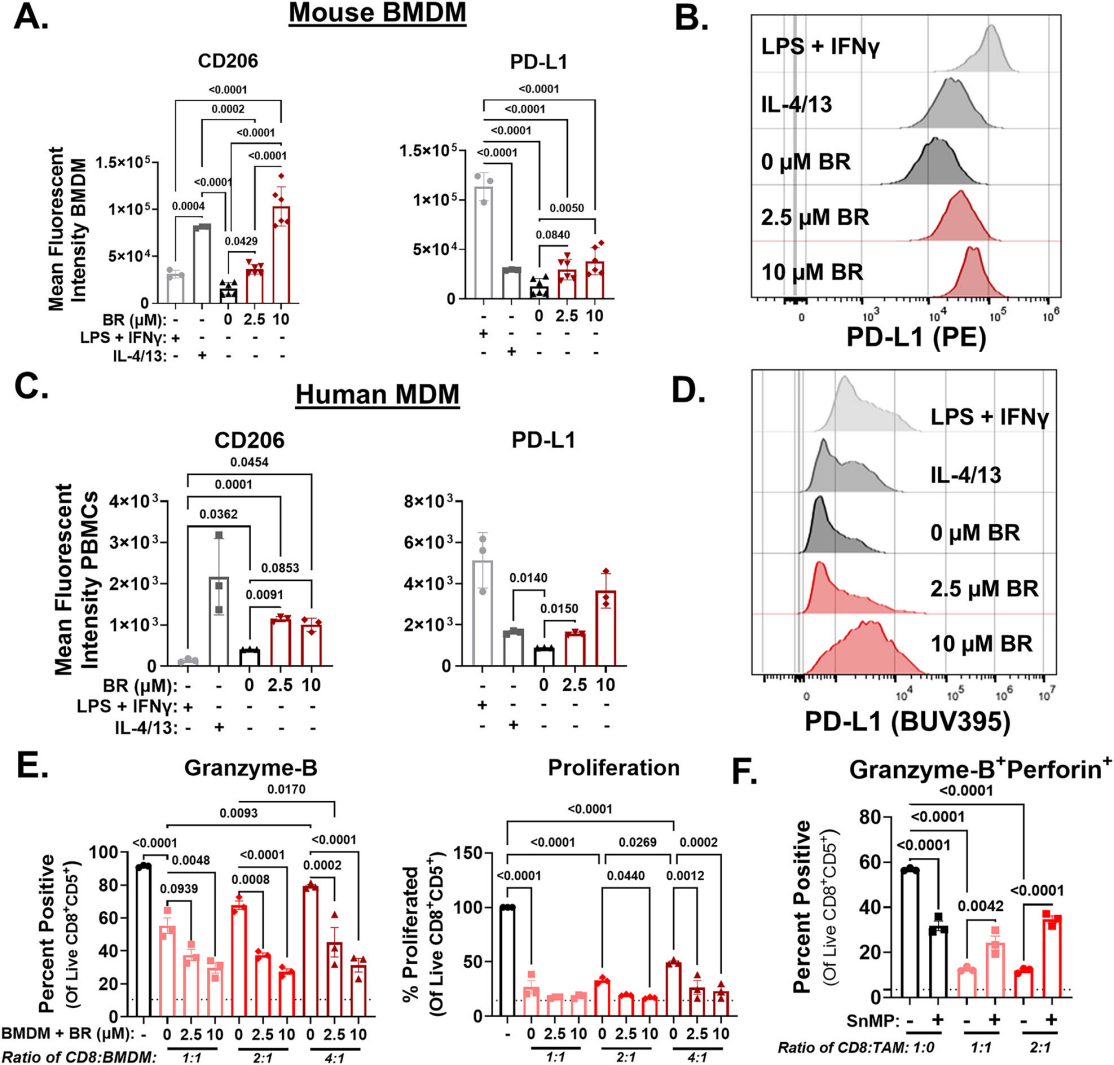
Bilirubin enhances macrophage immune suppression. BMDM were treated with positive controls to stimulate pro-tumor (20 ng/mL IL-4 and 20 ng/mL IL-13) or anti-tumor (100 ng/mL LPS and 20 ng/mL IFNγ) phenotypes or 0–10 μM bilirubin (BR) for 48 h. Live CD11b^+^F4/80^+^ cells were then analyzed for expression of pro-tumor (Arg1, CD206, PD-L1) and anti-tumor markers (iNOS) via flow cytometry. Shown is the mean fluorescence intensity (MFI) of CD206 and PD-L1 (**A**, n = 3-6, mean ± SD, one-way ANOVA with Tukey’s multiple comparison test). A representative flow plot for PD-L1 MFI is depicted in **B**. Human MDM were treated with positive controls to stimulate pro-tumor (20 ng/mL IL-4 and 20 ng/mL IL-13) or anti-tumor (100 ng/mL LPS and 20 ng/mL IFNγ) phenotypes or 0–10 μM bilirubin (BR) for 48 h. Live CD45^+^CD14^+^CD11b^+^ cells were then analyzed for expression of pro-tumor (CD206 and PD-L1) and anti-tumor markers (HLA-DR) via flow cytometry. Shown is the mean fluorescence intensity (MFI) of CD206 and PD-L1 (**C**, n = 3, mean ± SD, Brown-Forsythe and Welch ANOVA with Dunnett’s T3 multiple comparison test). A representative flow plot for PD-L1 MFI is depicted in D. **E** BMDM were cultured with 0–10 μM BR for 48 h after which they were co-cultured at varying ratios with CD8^+^ splenocytes stained with CellTrace Violet and treated with T cell-stimulating factors (CD28/CD3 dynabeads and IL-2). After 72 h co-culture, splenocytes were harvested and expression of cytotoxic molecules and CellTrace Violet was observed via flow cytometry in CD8^+^CD5^+^ cells (n = 3, one-way ANOVA with Tukey’s multiple comparison test). **F** CD11b^+^F4/80^+^ tumor-associated macrophages (TAMs) were isolated from 66Cl-4 mammary tumor-bearing mice treated with vehicle control or SnMP (25 mg/kg daily) for 8 days. CD8^+^ splenocytes were isolated from the same mice and co-cultured with TAMs in the presence of the T cell-stimulating factors described above. Percent CD8^+^CD5^+^ cells positive for cytotoxic markers was determined via flow cytometry after 72 h co-culture (n = 3, mean ± SD, one-way ANOVA with Tukey’s multiple comparison test).

We also measured expression of TAM markers CD206 and PD-L1 in BMDM. Bilirubin significantly increased the MFI of CD206 and PD-L1 when compared to control-treated BMDM (Fig. 5A, B). These findings were confirmed in CD14^+^ human peripheral blood mononuclear cells (PMBC) that were differentiated into macrophages or monocyte-derived macrophages (MDM) (gating strategy Supplementary Fig. 10A). Similar to BMDM, there was a significant increase in the number of live MDM after 10 μM compared to 0 μM bilirubin treatment (Supplementary Fig. 10B). Additionally, bilirubin treatment increased the MFI of CD206, PD-L1, and HLA-DR on MDM when compared to vehicle control (Fig. 5C, D, Supplementary Fig. 10C), demonstrating that bilirubin alters the expression of immunomodulatory cell surface proteins on macrophages.

To functionally test the impact of bilirubin on macrophage-mediated immune suppression, we isolated and differentiated BMDM. After 48 h bilirubin treatment, we co-cultured BMDM with stimulated CD8^+^ splenocytes isolated from different wild-type mice at three ratios. Prior to co-culture T cells were dyed with CellTrace Violet. At 72 h co-culture, T cells were harvested and analyzed via flow cytometry for dilution of CellTrace Violet (signifying proliferation) and the cytotoxic markers Granzyme-B and Perforin (gating strategy in Supplementary Fig. 11A). An isotype control antibody was used in a similar T cell activation assay to ensure that the detected CD8a staining was specific (Supplementary Fig. 11B). T cells co-cultured with bilirubin-treated BMDM had a significant decrease in Granzyme-B^+^ cells when compared to those co-cultured with control-treated BMDM (Fig. 5E). Although few splenocytes were Perforin^+^ in this experiment (Supplementary Fig. 11C), bilirubin-induced BMDM also limited T cell proliferation (Fig. 5E), suggesting that bilirubin-exposed macrophages have a higher suppressive capacity. To ensure bilirubin, which may persist in the media after BMDM treatment, did not directly affect T cells, a T cell stimulation assay was conducted and CD8^+^ splenocytes were directly treated with bilirubin. Interestingly, bilirubin treatment significantly increased the percent Granzyme-B^+^Perforin^+^ T cells when compared to control-treated cells (Supplementary Fig. 11D), suggesting that bilirubin did not directly impact T cells. Since we showed that bilirubin induces PD-L1 on BMDM and MDM, we repeated this experiment in the presence of a PD-L1 blocking antibody. While bilirubin significantly decreased the percent Granzyme-B^+^Perforin^+^ T cells when compared to control treatment, the addition of αPD-L1 rescued levels of these cytotoxic molecules (Supplementary Fig. 11E). These results suggest that bilirubin mediates macrophage immune suppression in part via upregulation of PD-L1.

Finally, we tested whether inhibition of HO-1 may limit macrophage immune suppression by conducting a similar co-culture experiment with TAMs isolated from 66Cl-4 mammary tumors from mice treated with SnMP or vehicle control. Splenocytes were isolated from the same animals and co-cultured with matching TAMs. After 72 h co-culture, T cells isolated from SnMP-treated mice cultured alone had decreased production of cytotoxic molecules when compared to CD8^+^ splenocytes isolated from control-treated counterparts (Fig. 5F), suggesting a direct impact of SnMP on T cells. Co-culture of TAMs with CD8^+^ splenocytes isolated from control-treated animals significantly decreased the number of CD8^+^ cells expressing Granzyme-B and Perforin when compared to control-treated CD8^+^ splenocytes culture alone. However, the addition of TAMs from SnMP-treated animals did not impact the percent Granzyme-B^+^Perforin^+^ splenocytes when compared to SnMP-treated CD8^+^ splenocytes culture alone. These findings suggest that macrophages in tumors with HO-1 inhibition and subsequent bilirubin depletion may be less immune suppressive.

### Tumor-HO-1 inhibition alters the macrophage microenvironment in combination with αPD-1

**Fig. 6:**
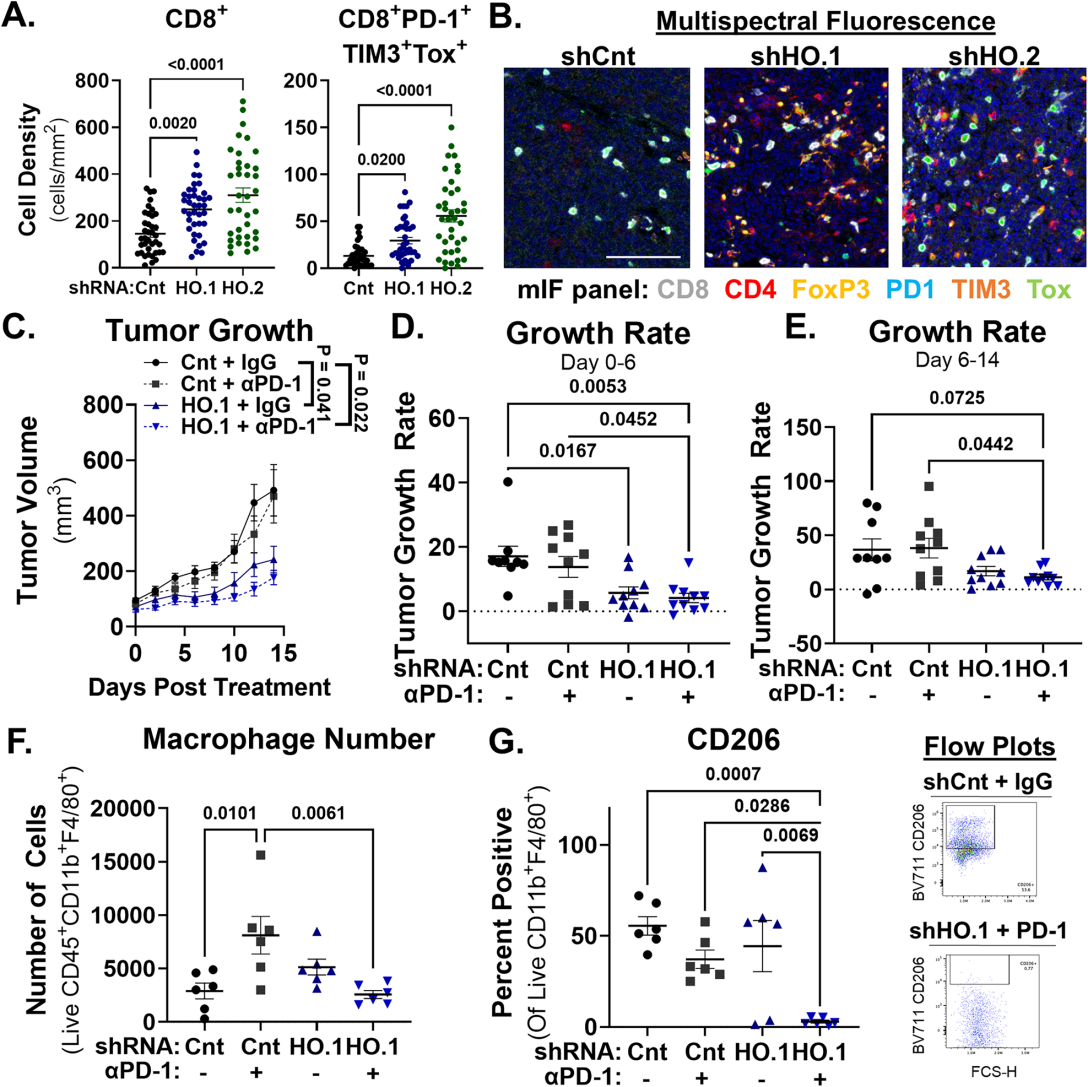
Tumor-HO-1 inhibition alters the macrophage microenvironment in combination with αPD-1. Suppressive T cells were observed in 66Cl-4 shCnt, shHO.1, and shHO.2 mammary tumors using a custom multispectral fluorescence panel (CD8/gray, CD4/red, FoxP3/yellow, PD-1/cyan, TIM3/orange, TOX1/2/green, scale bar = 100 microns). Shown is the quantification for the density of CD8^+^ cells and exhausted (PD-1^+^TIM3^+^Tox^+^) CD8^+^ cells (**A**) and representative images (**B**, n = 37–38 per group, mean ± SEM, one-way ANOVA with Tukey’s multiple comparison test). Mice bearing bilateral 66Cl-4 shCnt or shHO.1 tumors were treated when average tumor volume reached 80 mm^3^ with IgG control or αPD-1 (250 µg every three days, i.p. injection, n = 10 per group, total mice n = 20). Shown is the tumor volume (**C**, mean ± SEM, one-way ANOVA with Tukey’s multiple comparison test of area under the curve) and growth rate of tumors for the first half (day 0 – day 6, **D**) and second half (day 6 – day 14, **E**) of treatment (one-way ANOVA with Tukey’s multiple comparison test). Tumor-associated macrophages were analyzed via flow cytometry. Shown is the number of macrophages (**F**, n = 6 per group, mean ± SEM, one-way ANOVA with Tukey’s multiple comparison test) and the percent CD206^+^ macrophages (**G**, mean ± SEM, one-way ANOVA with Tukey’s multiple comparison test). Representative flow plots of CD206^+^ macrophages are also shown.

Our data suggest that tumor-HO-1 supports a suppressive microenvironment and immunotherapy resistance. Thus, we assessed the effects of HO-1 inhibition on immune cells that are targeted by checkpoint inhibitors. Using a mIF panel, we observed markers for suppressive macrophages (F4/80^+^PD-L1^+^) and exhausted T cells (CD8^+^PD-1^+^Tox^+^) in 66Cl-4 tumor-bearing mice treated with SnMP or vehicle control. SnMP treatment significantly decreased PD-L1^+^ macrophage density (Supplementary Fig. 12A). However, there was no change in exhausted T cell density between groups. We conducted a similar analysis in 66Cl-4 shCnt, shHO.1, and shHO.2 tumors using a mIF panel to quantify exhausted T cells (CD8^+^PD-1^+^Tim3^+^Tox^+^) and regulatory T cells (CD4^+^Foxp3^+^). Although CD4^+^ cell density did not change between groups, the density of regulatory T cell was significantly increased in shHO.1 tumors when compared to controls (Supplementary Fig. 12B). Interestingly, CD8^+^ T cells and exhausted T cells were not spatially restricted to the stroma or periphery in any of the treatment groups, but they were significantly increased in *Hmox1* knockdown tumors when compared to shCnt tumors (Fig. 6A, B, Supplementary Fig. 12B). Together, this data suggest that tumor-HO-1 inhibition dramatically impacts the TME and may be preferred over SnMP to prime tumors for immune checkpoint blockade sensitivity.

We introduced 66Cl-4 cells into the mammary fat pads of syngeneic female mice and treated daily with SnMP by oral gavage and every three days with αPD-1 by intraperitoneal injection. Although there was no significant difference in tumor volume between any of the treatment groups (Supplementary Fig. 13A, B), tumor growth rate was significantly decreased in the combination therapy group after the first week of treatment (Supplementary Fig. 13C). However, the tumor growth rate increased in the combination group during the second week of treatment (Supplementary Fig. 13D), suggesting that the combination of HO-1/HO-2 and checkpoint inhibition may only temporarily delay tumor growth.

We next tested the combination of tumor-*Hmox1* knockdown and checkpoint inhibition. 66Cl-4 mammary tumor cells harboring shCnt or shHO.1 were introduced into syngeneic female mice, and animals were randomized to treatment with IgG control or αPD-1. Tumor growth was significantly decreased in both 66Cl-4 shHO.1 groups when compared to control counterparts (Fig. 6C, Supplementary Fig. 14A, B). When the growth rate was observed for the first half of treatment (Day 0-6), there was a similar growth rate between shCnt tumors treated with IgG or αPD-1 but the growth rate of *Hmox1* knockdown tumors was significantly decreased when compared to controls (Fig. 6D). This trend was maintained through the second half of treatment (Day 6–14, Fig. 6E), revealing that αPD-1 alone had little impact on tumor growth. Interestingly, H&E analysis of tumors at endpoint revealed that shHO.1 tumors from mice receiving αPD-1 were full of adipocyte-like structures and appeared to have fewer tumor cells (Supplementary Fig. 14C), demonstrating a possible impact on tumor progression. To test the effects of combination treatment on the TME, flow cytometry was conducted on TAMs at endpoint (CD11b, F4/80, Arg1, iNOS, CD206, PD-L1). Interestingly, the number of macrophages significantly increased in shCnt tumors treated with αPD-1, which was restored to baseline by *Hmox1* knockdown (Fig. 6F). The ratio of macrophages expressing the anti-tumor marker iNOS to those expressing the pro-tumor marker Arg1 was not altered in any treatment group (Supplementary Fig. 14D). However, there was a striking change in the percent CD206^+^ macrophages and the MFI of CD206 in shHO.1 tumors from mice receiving αPD-1 treatment when compared to all other treatment groups (Fig. 6G and Supplementary Fig. 14E). An opposite trend was seen in PD-L1 expressing macrophages (Supplementary Fig. 14F, G). An important pro-metastatic function of CD206^+^ macrophages is extracellular matrix (ECM) remodeling.^50^ Analysis of mammary tumors using Masson’s Trichrome staining revealed a significant decrease in collagen deposition within mammary tumors harboring *Hmox1* knockdown when compared to controls (Supplementary Fig. 15A). The density of Cox2^+^ macrophages, which can be induced by ECM^51^ and support breast tumor progression^52,53^, trended towards a decrease in HO-1-depleted mammary tumors compared to controls (Supplementary Fig. 15B, C). Together, these results suggest that *Hmox1* knockdown, particularly in combination with checkpoint inhibition, alters tumor progression by dynamically impacting the macrophage microenvironment.

## Discussion

To assess the impact of heme breakdown on the TME, we profiled human BC specimens and revealed an association between tumor-HO-1 and macrophages. Depletion of tumor-HO-1 in a TNBC-like mouse model limited tumor growth, metastasis, and intratumoral bilirubin levels. This study, the first to assess bilirubin levels in TIF and matching plasma, demonstrated that intratumoral bilirubin can be 10-fold higher than circulating levels. Importantly, interstitial bilirubin can be depleted by tumor-HO-1 inhibition. These findings suggest that breast tumors harbor a unique bilirubin-rich microenvironment. Our follow-up studies revealed that bilirubin enrichment decreased macrophage function and enhanced macrophage-mediated immune suppression demonstrating that bilirubin is a dynamic cancer immunometabolite.

Previous work showed that HO-1 blockade limits mammary tumor growth,^13,14,54^ metastasis,^12^ chemotherapy resistance,^14,55^ and immune suppression^55^ via direct effects on the myeloid compartment. Since the HO-1 inhibitors utilized in many of these studies were clinically tested in severe cases of jaundice, these findings garnered excitement for improving drug bioavailability^56^ to allow testing of HO-1 inhibitors in aggressive solid tumors. However, most pre-clinical studies were completed with BC models that lack tumor-HO-1 expression. Since we detected HO-1^+^ tumor cells in human BC specimens, we compared global versus tumor cell-specific HO-1 inhibition in a mammary tumor model that maintains HO-1 expression throughout tumor progression. While global HO-1 inhibition had little to no impact on tumor growth and lung metastasis, tumor-HO-1 inhibition delayed tumor growth and nearly depleted lung metastatic outgrowth. Similarly, immunotherapy treatment in combination with SnMP did not affect tumor growth. However, tumor-HO-1 depletion in combination with αPD-1 decreased the tumor growth rate and ablated a population of CD206^+^ macrophages that are known to remodel the TME to support tumor progression and lung metastasis.^50,57,58^

Our findings are supported by work in sarcoma, hepatoma, and melanoma models showing that inhibition of tumor-HO-1 limited disease progression, but depletion of HO-1 in immune cells increased tumor progression.^59,60^ Together, these results suggest that tumor-specific HO-1 inhibition may limit tumorigenesis more effectively than global HO-1 inhibition. However, this idea needs to be tested in additional models and tumor types, as the opposite phenotype was seen in some breast^61,62^ and ovarian cancer models.^56^ While the conclusion that effective HO-1 inhibition requires cell specificity could limit the feasibility of using HO-1 inhibitors in solid tumors, the rapid advancement of antibody-drug conjugates in BC opens the door for future preclinical and clinical testing of tumor-specific HO-1 inhibition in TNBC.^63^

Although further studies are needed, evidence in the literature suggests that tumor-specific HO-1 inhibition may be more effective than SnMP because HO-1 alters T cell function. HO-1 activation enhanced proliferation of CD4^+^ T cells,^64^ while HO-1 inhibition promoted the proliferation of Tregs and inhibited antigen specific CD4^+^ T cell expansion.^27,65^ These works are in line with our observation that splenocytes isolated from SnMP-treated mice had decreased expression of cytotoxic molecules compared to controls. Alternatively, other HO-1 metabolites, such as iron,^66^ have been shown to support anti-tumor T cell responses, which could be impacted by SnMP. These works highlight the need to expand our findings with additional studies that assess the impact of HO-1 inhibition on immune cell populations other than macrophages, including myeloid-derived suppressor cells, dendritic cells, and T cells.

Analysis of genes altered in macrophages by bilirubin suggests that this metabolite supports both NFκB signaling and a dysfunctional macrophage phenotype. In follow-up studies, we assessed macrophage expression of immune suppressive PD-L1, pro-metastatic CD206, and the efferocytosis receptor MerTK in addition to canonical polarization markers iNOS and Arg1. The combination of *Hmox1* knockdown and checkpoint inhibition dramatically impacted expression of PD-L1 and CD206. Interestingly, others showed that increased PD-L1 and decreased CD206 expression in macrophages, as seen in our study, supports anti-tumor CD8^+^ T cell activity.^67,68^ Conversely, expression of macrophage polarization markers was not significantly altered in any of our treatment groups. These findings are consistent with the idea that macrophages are more heterogenous and diverse than the binary M1/M2 macrophage polarization model and led us to focus on testing the effects of bilirubin on macrophage function. Importantly, this is the first report to demonstrate that bilirubin blocks macrophage dead cell engulfment. Although the effects of efferocytosis on tumor progression are debated,^69^ these results, in addition to our finding that bilirubin supported macrophage-mediated T cell suppression, imply that macrophages in the bilirubin-rich TME may lose the ability to perform physiologic functions but gain the ability to dampen anti-tumor immunity. Others have demonstrated that breast tumor-HO-1 modulates response chemotherapy^70^ and immunotherapy^15^ by directly impacting T cell cytotoxicity. Our work expands these findings and reveals a complimentary mechanism that may be utilized by aggressive BC to reshape the microenvironment.

Although further exploration of how bilirubin mechanistically impacts macrophages is needed, our result and the work of others implicate NFκB signaling.^71^ However, additional studies showed that bilirubin limits NFκB activation in T cells and peritoneal macrophages.^27,72^ These disparate findings may be due to vastly different bilirubin concentrations utilized in varying contexts (with and without treatment with inflammatory LPS). Although we observed that macrophage efferocytosis was altered by a wide range of bilirubin concentrations (0-100 µM), dose dependency needs to be assessed in future studies that will first evaluate levels of bilirubin in human TIF to provide a clinically relevant intratumoral concentration. After which, dose response to physiologic bilirubin concentrations should be assessed to evaluate macrophage survival, efferocytosis, and immune suppression.

Collectively, these data demonstrate that tumor-HO-1 and its metabolite bilirubin support breast tumor progression and immune modulation. Bilirubin dynamically impacted both macrophage physiologic and tumor-associated functions. Our findings regarding the bilirubin-enriched TME demonstrate that HO-1 and bilirubin are an underappreciated immune modulatory axis in cancer. Since several types of cancer rely on heme metabolism to support oncogenesis, a phenomenon termed porphyrin overdrive,^73^ these findings may broadly apply to other cancers and suggest that bilirubin may be an important signaling metabolite in cancer.

## Methods

### Accessing and mining of publicly available datasets

Data from The Cancer Genome Atlas and METABRIC^32–34^ were accessed using CBioPortal (on April 10, 2022). mRNA expression (normalized z-score relative to all samples) of enzymes in the heme metabolism pathway (*HMOX1*, *HMOX2*, *BLVRA*, *BLVRB*) were downloaded and presented for specimens representing normal breast tissues and each breast cancer subtype based on the PAM50 molecular classification (TNBC includes basal and claudin-low, ER^+^ includes Luminal A and B). The same dataset was analyzed using the CIBERSORT analysis tool (on April 17, 2022) to predict relative immune call abundance in breast cancer and normal breast specimens using the LM22 reference gene set for twenty-two immune cell types.^37^ Presented are immune cell abundance scores for specimens in the top quartile or lowest quartile of *HMOX1* expression for each breast cancer subtype and normal tissues. For KM Plotter analysis, the overall survival out to 120 months of all breast cancer patients in the KM Plotter breast cancer protein database^36^ was plotted after stratification by HO-1 expression (HMOX1; Uniprot ID: P09601, conducted on June 20, 2024). Overall survival was further stratified based on lymph node positivity or negativity. The best median cutoff was used for all plots.

### Tissue analysis

For immunohistochemistry analysis, tissues were sectioned (5 microns) and heat immobilized on slides for 1 h at 60 °C. Tissues were then deparaffinized and re-hydrated with xylene and ethanol. After which heat-induced epitope retrieval (HEIR) was conducted using pH 6.0 or Tris/EDTA pH 9.0 buffer, followed by blocking of endogenous peroxidases with 3% hydrogen peroxide for 5 min and non-specific antigens with 2.5% normal goat serum (Vector #MP-7451) for 20 min. Primary antibody was diluted in TBST and applied to tissues for 1 h (HO-1: Abcam, AB13243, 1:400; Ki67, Abcam, AB15580, 1:1600; CC-3, Cell Signaling, 9661, 1:400). After which, tissues were washed with TBST (3x) then incubated with anti-rabbit secondary polymer (Vector #MP-7451). Tissue was washed with TBST (3x) prior to application of DAB (Vector #SK-4105) for 4 min and tissue dehydration. Hematoxylin (1:5, Anatech, 842) was used as a counterstain. For the tissue microarray (TMA), tissue samples were provided by the Cooperative Human Tissue Network (CHTN), which is funded by the National Cancer Institute. Other investigators may have received specimens from the same subjects. Prussian blue and Masson’s trichrome staining were conducted by the Research Histology Services of the Pitt Biospecimen Core. After staining, whole slides were scanned using the Aperio AT2 microscope and whole tumors or 1–4 tissue cores per specimen for the TMA were analyzed using ImageScope Software and the Positive Pixels Count v9 Macro. For quantification of HO-1 in the TMA we exclude weak 1+ staining in our analysis because a background level of HO-1 was detected in all tissues. For immunofluorescence, tissues were sectioned and deparaffinized/dehydrated as mentioned above. HEIR was conducted before and after each antibody incubation/detection using Akoya buffers (AR9 AR9001KT and AR6 AR6001KT). Primary antibodies included (Human - CD4, Dako, 4B12, 1.4 μg/mL; FoxP3, Abcam, 236 A/E7, 1:400; HO-1, Abcam, ab13243, 1:800; CD8, Dako, C8/144B, 0.4 μg/mL; Cd68, Dako, KP1, 0.12 μg/mL; PanCK, DAKO AE1/AE3, 0.18 μg/mL; Mouse – F4/80, Thermofisher, MF480000, 1:12800; PD-L1, Cell Signaling, 64988, 1:50; PD-1, Cell Signaling, 84651, 1:400; CD8a, Thermofisher, clone 4SM15, 1:1600; Tox/Tox2, Cell Signaling, 73758, 1:12800; CD206, Sigma, HPA004114, 1:2000; iNOS, Cell Signaling, D6B6S, 1:1600; Arg1, Cell Signaling, D4E3M, 1:12800; COX2, Cell Signaling, D5H5, 1:300) and secondary antibodies (Human—Anti-mu/rb Akoya ARH1001EA; Mouse - Vector MP-7451 or MP-7444). For coIF, TSA reagents were used to amplify signal (Perkin Elmer FITC, TS000200 1:100; Perkin Elmer, Cy3.5, TS-000202, 1:500) and for multiplex IF the Akoya Opals (Opals 520, 540, 570, 620, 690 NEL811001KT) and buffers were used to amplify fluorescent signal. Spectral DAPI (Akoya, FP1490) was used as a counterstain. Whole slides were scanned using the Vectra 3 microscope and an average of 5 fields per tumor tissue or 4 tissue punches for the TMA were assessed using InForm and Phenoptr Software. The suppressive T cell panel staining was completed with an autostainer in the Translational Pathology Imaging Laboratory at the UPMC Hillman Cancer Center. To assess metastatic burden, FPPE lungs were serially sectioned and three sections, each 50 microns apart, underwent hematoxylin and eosin staining. Whole slides were scanned on the Aperio AT2 microscope and ImageScope software was used to assess the number of metastases and area of each metastasis, which was averaged across serial sections.

### Tissue culture

All cells were grown in a humidified chamber supplemented with 5% carbon dioxide and kept at 37 °C. 66Cl-4 cells were grown in DMEM-high glucose (Corning: SH30243.01) supplemented with 1% penicillin-streptomycin (pen/strep, Fisher Scientific: 15140-122), 1% L-glutamine (Fisher Scientific: 25030-081), 1% non-essential amino acids (NEAA, Fisher Scientific: 11140-050), and 10% fetal calf serum (FCS, HyClone: SH30073.03). RAW264.7 mouse macrophages were grown in DMEM-high glucose supplemented with 1% pen/strep and 10% fetal bovine serum (FBS, PEAK purchased from CU AMC Tissue Culture Core). THP-1 human-derived macrophages and MH-S alveolar macrophages were cultured in RPMI (Gibco, 11875-093) supplemented with 1% pen/strep and 10% FBS. The human TNBC cell line BT549 was grown in RMPI supplemented with 1% pen/strep, 1% NEAA, 10% FBS, and 5 μg/mL insulin (Sigma: I6634). All human cell lines were authenticated by short tandem repeat analysis (Promega) at the University of Colorado Anschutz Medical Campus (CU AMC) Tissue Culture Core in 2019. All lines were regularly tested for Mycoplasma (every 3–6 months). Cells were passaged at 70–80% confluence using 0.25% trypsin (ThermoFisher: 15090046). The exception was THP-1 cells that grow in suspension and were passaged using centrifugation. To knockdown *Hmox1*, 66Cl-4 cells were plated in a 12-well plate. The following day, when cells were at 50% confluence, they were treated with increasing volumes of lentiviral particles prepared by the CU AMC Functional Genomics Shared Resource containing a MISSION pLKO.1-puro lentiviral vector with three sequences against *Hmox1* (TRCID: TRCN0000234075, TRCN0000234076, or TRCN0000234077) or a non-target control sequence (SHC216). At the same time, cells were also treated with 5 μg/mL polybrene. 16 h later, media was removed and replaced with fresh normal growth media. On day 5 post-transfection, cells were treated with 2 μg/mL puromycin. After which, cells were passaged as described above in the presence of puromycin except for when plating to expand for animal studies or analysis of tumorigenic properties. No cell lines were passaged more than eight times after thawing. After thawing and prior to each experiment, *Hmox1* knockdown was confirmed by western blot using the Odessey CLx imaging system (primary antibodies: HO-1—Abcam, ab13243, 1:1000 and beta-actin—Cell Signaling Technologies, 3770, 1:10,000; secondary antibody—LiCOR, Goat anti-rabbit 800 or Goal anti-mouse 680, 1:5000). All experiments were conducted using at least biological and technical triplicates.

### Animal studies

All animal experiments were performed in accordance with a protocol (#1056) approved by the CU AMC Institutional Animal Care and Use Committee using humane procedures. We have complied with all relevant ethical regulations for animal use. 200,000 66Cl-4 parental, shCnt, shHO.1, or shHO.2 cells were injected into the 4th inguinal mammary fatpads of 8-week-old host female BALB/CJ or BALB/CJ RAG2 KO/IL2Rgc KO/SIRPa(NOD) mice. Tumor volume was monitored after tumors became palpable every other day using calipers ((volume = 1/2(width^2^ × height)). When applicable, animals were randomized and tumor volume matched using excel, and treatment was initiated when average tumor volume reached 80 mm^3^. 25 mg/kg SnMP (Cayman Chemical, 19071) or vehicle control (4.5% sodium phosphate in sterile saline) were delivered daily by oral gavage. 250 μg/mouse anti-PD-1 antibody (BioXCell, RMP1-14) or IgG control antibody (BioXCell, HRPN) was delivered by intraperitoneal injections every 3 days. Endpoint was either when tumor volume reached over 500 mm^3^ per group or 2 weeks treatment. This tumor volume did not exceed the maximal allowed tumor volume permitted in our approved IACUC protocol of 1500 mm^3^. At endpoint, tumors were either formalin-fixed paraffin-embedded (FFPE) for histological analysis, centrifuged to collect interstitial fluid as in ref. ^74^, or digested into single cells using 500 units of Collagenase II and Collagenase IV (ThermoFisher, 17101015 and 17104019) and 2 mg/mL DNase (Sigma, 11284632001) to analyze by flow cytometry. At the same time, lungs were inflated with 10% formalin, then paraffin embedded for histological analysis, and blood was collected using a cardiac puncture and placed into EDTA coated tubes (FisherScientific, NC9414041) that were centrifuged as described in ref. ^74^ to collect plasma for mass spectrometry analysis of biliverdin and bilirubin by the CU AMC Mass Spectrometry Metabolomics Shared Resource. Since BC is most often diagnosed in women, female mice were used for all of these studies that included a total of 102 animals. Researchers administering treatments were aware of group allocation but those assessing the outcome and conducting data analysis were blinded to treatment groups. Confounders like treatment order, measurements, or animal/cage location were not controlled for. Mice were housed in the vivarium and were cared for by the University of Colorado Division of Animal Care. The facilities are AAALAC-accredited, and a full-time veterinarian was available for consultation on any problems arising with mice.

### Assessing macrophage function and gene expression

Bone marrow was flushed from the hind tibia and femurs of 8-week old female BALB/CJ mice using the protocol detailed in ref. ^75^. After which bone marrow was filtered with a 40-micron filter and pelleted via centrifugation. Pellets were treated with 2 mLs ACK (150 mM Ammonium Chloride, 1 mM Potassium Bicarbonate, 0.1 mM EDTA, pH 7.4) to lyse red blood cells, which was quenched with the addition of 10 mL of HBSS. Cells were washed 2x with HBSS (400 mg/L KCl, 60 mg/L KH_2_PO_4_, 350 mg/L NaHCO3, 8000 mg/L NaCl, 48 ng/L Na_2_HPO_4_, 1100 mg/L dextrose anhydrase) then resuspended in 10 mLs growth media (RMPI with 10% FBS and 1% pen/strep) and plated (>1.125 × 10^6 cells/ petri dish or 15,000 cells/well in 96 well plates). To differentiate cells into macrophages, they were treated with 25 ng/mL M-CSF (Shenandoah, 200-08) upon plating and again 2 days later. After 5 days, BMDM were treated with bilirubin or DMSO control, 20 ng/mL IL-4 and IL-13 (to support pro-tumor macrophage polarization, Shenandoah 200-18 and 200-22) or 20 ng/mL IFNγ and 100 ng/mL LPS (to support anti-tumor macrophage polarization, Sigma, L5668). For analysis by qRT-PCR, after 48 h treatment with bilirubin, mRNA was isolated from BMDM using TriZOL extraction as recommended by the manufacturer (FisherScientific, 15596-018). Using the manufacturer’s protocol, 1 μg of total mRNA was reverse transcribed into cDNA using QScript (Quantabio Cat# 95048-025). qRT-PCR was used to determine the relative expression (∆∆CT) of genes of interest after normalization to *Gapdh* using PowerUP SYBR Green Master Mix (Thermo Fisher Cat# A25742), a 7500 Fast Real-Time PCR System (Applied Biosystems), and 7500 Software (ver2.3, RRID:SCR_014596). A full list of primers is in Supplemental Table 7. In a separate experiment, mRNA was similarly isolated from BMDM using TriZOL and a Poly A-selected total RNA library was prepared after mRNA extraction and sequenced using the NOVASEQ 600 (paired-end reads at 40 million read pairs) by the CU AMC Genomics and Microarray Shared Resource. After which, RNA-seq data were processed using the nf-core RNAseq pipeline (v3.12.0)^76^. Briefly, Illumina adapters were removed using Cutadapt (v3.4)^77^ as part of the trimgalore (0.6.7) package.^78^ Reads were aligned using STAR (v2.7.9a)^79^ to the Ensembl mouse transcriptome (GRCm39 release 104) and quantified using Salmon (v1.10.1).^80^ Raw data with counts by gene were generated using tximport^81^ on salmon quantified data. Normalized data was generated to counts per million.^82^ Differential expression was calculated using the limma R package using the mouse identifier as a covariate.^83^ Heatmaps were generated with GraphPad Prism (v10.2.3 for Windows, GraphPad Software, Boston, Massachusetts USA) following z-score normalization of counts per million. Gene set enrichment analysis (GSEA) was performed using fold-change and the fgsea R package^84^ with Hallmark gene sets from the Molecular Signatures Database,^85^ which were downloaded using the msigdbr R package^86^. Over-representation analysis was performed using DAVID^87,88^ with significantly (FDR < 0.05) upregulated and downregulated genes. The RcisTarget^89^ R package was used to calculate transcription regulator enrichment in genes upregulated with bilirubin treatment with a *p*-value < 0.01. For efferocytosis assays, BMDM were plated and differentiated in 96-well plates. At the same time as bilirubin treatment, dead 66Cl-4 tumor cells were added. Tumor cells were killed with 10 μM staurosporine (Medchem Express, HY-15141) overnight and only cells floating in the media were collected via centrifugation the following day. Dead cells were then stained with 0.625 μg/mL pHrodo according to the manufacturer’s protocol (Sartorius, 4649). Dead cells were added to macrophages with appropriate concentrations of bilirubin or BAY 11-7082 (1.5 μM, Cayman Chemical, 10010266) and red signal was immediately monitored with the IncuCyte Zoom imaging system every 30 min for up to 24 h. IncuCyte Software was used to assess the area and intensity of red signal to generate an efferocytosis capacity score. For mouse RAW264.7 macrophages, the same experimental design mentioned above was followed, expect RAW264.7 macrophages were plated the day before bilirubin treatment. For THP-1 monocyte-derived macrophages that grow in suspension, cells were differentiated into macrophages with 24 h 1.0 μg/mL phorbol 12-myristate 13-acetate (PMA) treatment prior to the addition of bilirubin for 48 h. To repeat studies utilizing human-derived macrophages, CD14^+^ cells were isolated from healthy female de-identified human PBMCs purchased from Stem Cell (200-0077) using positive selection microbeads according to the manufacturer’s protocol (Miltenyi, 130-050-201). Cells were plated (>750,000 cells/well in non-tissue culture treated 6-well plates) and maintained in 1.5 mL growth media (RPMI + 20% FBS + 1% pen/strep) supplemented with 100 ng/mL M-CSF. Two days later, media was refreshed and an additional 100 ng/mL M-CSF was added. After 5 days, MDM were treated with bilirubin or DMSO control, 20 ng/mL IL-4 and IL-13 (to support pro-tumor macrophage polarization, Life Technologies 200-04 and 210-13) or 20 ng/mL IFNγ and 100 ng/mL LPS (to support anti-tumor macrophage polarization, Life Technologies, 300-02 and Sigma, L5668).

### T cell stimulation assays

BMDM were isolated, differentiated, and treated with bilirubin as mentioned above in 96-well U-bottom plates (Falcon, Fisher Scientific, 08-772-54). After 48 h of bilirubin treatment, CD8^+^ splenocytes were added to BMDM. Splenocytes were isolated from the spleens of 3–5 healthy wild-type 8-week-old BALB/CJ mice by filtration and purification using LS MACS columns and a CD8a^+^ T cell isolation kit (Miltenyi Biotec, 130-104-075). Prior to plating, T cells were stained with CellTrace Blue or Violet according to the manufacturer’s suggestions (Fisher Scientific, C34574). Then, various concentrations of T cells were added to differentiated BMDM with CD3/CD28 dynabeads used as recommended by the manufacturer (Fisher Scientific, 11456D) and 7.5 ng IL-2 (Shenandoah Biotechnology, 200-17). At this time 10 μg/mL of an IgG control or αPD-L1 antibody was also added (BioXCell, clone LTF-2 and 10 F.9G2). Three days after plating T cells, co-cultures were treated with 0.25 mg brefeldin A (Thermofisher, J62340.MA) for 4–6 h prior to harvesting. Non-attached T cells were collected by pipetting for flow cytometry analysis. For co-culture of tumor-associated macrophages and splenocytes, macrophages were isolated from digested 66Cl-4 mammary tumors using a monocyte isolation kit (Miltenyi Biotech, 130-100-629) followed by a F4/80 isolation kit (Miltenyi 130-110-443). Splenocytes were isolated from the spleens of the same mice. Macrophages and splenocytes from mice receiving the same treatments (either SnMP or vehicle control) were pooled, then plated at varying ratios as described above.

### Flow cytometry

T cells were collected and resuspended in 1x PBS containing Zombie UV (Biolegend, 1:1500). After incubation at room temperature for 20 min, cells were washed twice with FACS buffer (1% Sodium Azide from 10% stock, 1% HEPES, 2% heat inactivated FBS in 1x PBS), then incubated with primary antibodies to cell surface markers (CD8a – Biolegend, 100714, 0.3 μL/well or isotype control—Biolegend 400523, 0.3 μl/well, CD5 – Biolegend, 100625, 0.625 μL/well or CD3 – Biolegend, 100235, 0.625 μL/well) for 30 min in the dark on ice. Afterwards cells were washed with FACS buffer then fixed and permeabilized following the manufacturer’s protocol for the BD cytofix/cytoperm kit (BDB554714). Cells were then incubated for 30 min on ice in the dark with intracellular markers (Perforin – Biolegend, 154305, 0.375 μL/well; Granzyme-B – Biolegend, 515403, 0.75 μL/well). Stained cells were analyzed on the Penteon NovoCyte flow cytometer after conducting an AutoCompensation adjustment for spectral spillover on single stain control samples. Fluorescence minus one (FMO) controls were used to assist with the gating strategy. A similar approach was used to assess BMDM and macrophages isolated from mammary tumors using Zombie Yellow as a live/dead stain and the following panel of cell surface (CD11b – Thermofisher, 364-0112-80, 0.625 μL/well; F4/80 – Biolegend, 123137, 0.375 μL/well; PD-L1—Biolegend, 124307, 0.25 μL/well; MerTK – Fisher Scientific, FAB5912U, 0.625 μL/well) and intracellular (Arg1—Thermofisher, 53-3697-80, 0.3 μL/well; CD206—Biolegend, 141727, 0.45 μL/well; iNOS—Thermofisher, 17-5920-80, 0.09 μL/well) markers. For human MDM, Zombie UV was used as a live/dead stain and then cells were stained with the following panel of cell surface (CD45—Biolegend, 304023, 0.3 μL/well; CD14 – Biolegend, 301817, 0.75 μL/well; CD11b – Biolegend, 301345, 0.75 μL/well; HLA-DR – Biolegend, 980406, 0.75 uL/well; PD-L1 – Fisher Scientific, BDB568621, 0.75 μL/well;) and intracellular (CD206 – Biolegend, 321105, 0.75 μL/well) markers.

### Statistics and reproducibility

Sample size calculations for all mouse experiments were conducted under the supervision of biostatistician Dr. Kathleen Torkko using published metastasis number data.^12^ A total sample size of 8 mice per group was calculated to achieve 80% power to detect differences among the means versus the alternative of equal means using an F test with a 0.05 significance level. All data were presented as the mean ± standard error of the mean (SEM) or standard deviation (SD) using GraphPad Prism software (version 10.2.3 for Windows, GraphPad Software, Boston, Massachusetts, USA). All data points were shown in column graphs except when the sample size exceeded 50 and a violin plot was used. Significance was determined to be *P* < 0.05 using an unpaired two-tailed T cells, or when appropriate a paired two-tailed T test, when assessing two groups. A one-way ANOVA with Tukey’s or Dunnett’s multiple comparison test was used when there were three or more groups. Values below or above two standard deviations from the mean were excluded as outliers. When possible, animal studies were repeated twice and tumors were excluded from all analyses if they did not take, which only occurred for one tumor in the data presented in Fig. 6C from an animal treated with vehicle control and IgG. Shown is a representative of two or more separate experiments.

### Reporting summary

Further information on research design is available in the Nature Portfolio Reporting Summary linked to this article.

## Supplementary information

Transparent Peer Review file

Supplementary Information

Reporting Summary

## Data Availability

All raw and source data including source data for animal experiments are available on FigShare (doi.org/10.6084/m9.figshare.31014661 and doi.org/10.6084/m9.figshare.31012045).^90^ Raw and processed RNA-seq data were deposited in the Gene Expression Omnibus (GSE277310)^91^ and metabolomics data were deposited in MetaboLights (MTBLS14278).^92^ Unedited and uncropped western blot images can be seen in Supplementary Fig. 16.
