# Supplementary material for: Aggressive breast cancers secrete heme metabolites to alter macrophage immune suppression and function
Source: Commun Biol. 2026 May 9;9:977. doi: 10.1038/s42003-026-10212-0 (PMC13376764; doi:10.1038/s42003-026-10212-0)
Supplement: Supplementary file 2 — Supplementary Information [file 42003_2026_10212_MOESM2_ESM.pdf]

SUPPLEMENTAL INFORMATION

METHODS

Analysis of cell proliferation, soft agar growth, and migration. For proliferation assays, 2,000 66Cl-4 shCnt, shHO.1, shHO.2, or shHO.3 cells were plated (N = 6-10 wells/group/experiment) in normal growth media in a 96-well plate (Falcon). 24 hours later, cell growth was monitored every four hours using the whole-well image setting on the IncuCyte Zoom Imaging System and the IncuCyte software was used to quantify relative percent confluence (relative to the first scan). To assess growth in soft agar, 1.0% agar was mixed 1:1 with 2x 66Cl-4 media (DMEM-high glucose with 20% FCS, 2% NEAA, pen/strep, and L-glutamine). Once hardened, 7,500 66Cl-4 shCnt, shHO1.1, shHO1.2, or shHO1.3 cells per well (N = 3/group/experiment) were plated in 2x 66Cl-4 media diluted 1:1 with 0.6% agar. After hardening, soft agar was overlain with normal growth media that was replaced every 7 days. At 14 days growth, colonies were fixed overnight in the incubator using 1 mg/mL NBT (Sigma, N5514). After fixing, colonies were imaged using the Gel Count imaging system (Scintica) and both the number of colonies and average colony size were quantified using ImageJ software.

Conditioned Medium Experiments. 200,000 tumor cells (66Cl-4 for mouse and BT549 for human) were plated per well in a 6-well dish. The following day, cells were treated with the appropriate amount of SnMP (10 μM for mouse and 40 μM for human). 72 hours post-treatment, conditioned medium was collected from each well and centrifuged to remove any cell debris. 1 mL conditioned medium and 1 mL fresh macrophage media were added to macrophages that were plated and/or differentiated the prior day. At the same time, cells were treated with 0 – 100 μM bilirubin. After 48 hours treatment, mRNA from macrophages was harvested using TriZOL and mRNA expression of target genes was determined using qRT-PCR.

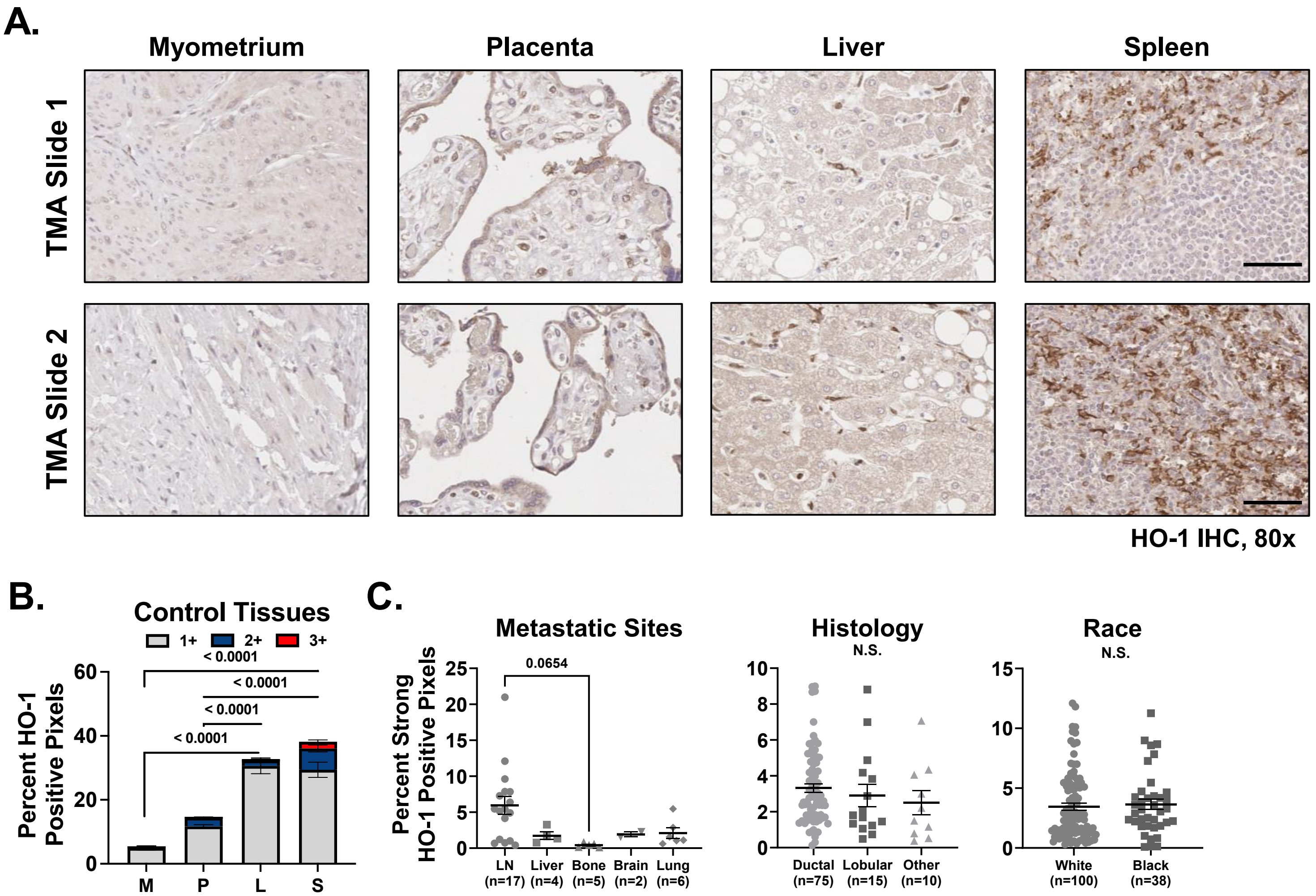

Supplementary Figure 1. Expression of HO-1 in control tissues and at various breast cancer metastatic sites. HO-1 protein expression determined via IHC in the CHTN_BrCaStg1 tissue microarray. **A-B.** Shown are representative images of control tissues from each TMA slide (A) and quantification of the percent 1^+, 2^+, and 3^+ positive staining for each tissue type (B, mean ± SEM, two-way ANOVA with Tukey's multiple comparison test). **C.** The percent 2^+ and 3^+ positive pixels for HO-1 in metastases is depicted after stratification by site and for all specimens stratified by tumor histology and patient race (mean ± SEM, one-way ANOVA with Tukey's multiple comparison test or unpaired two-tailed T test).

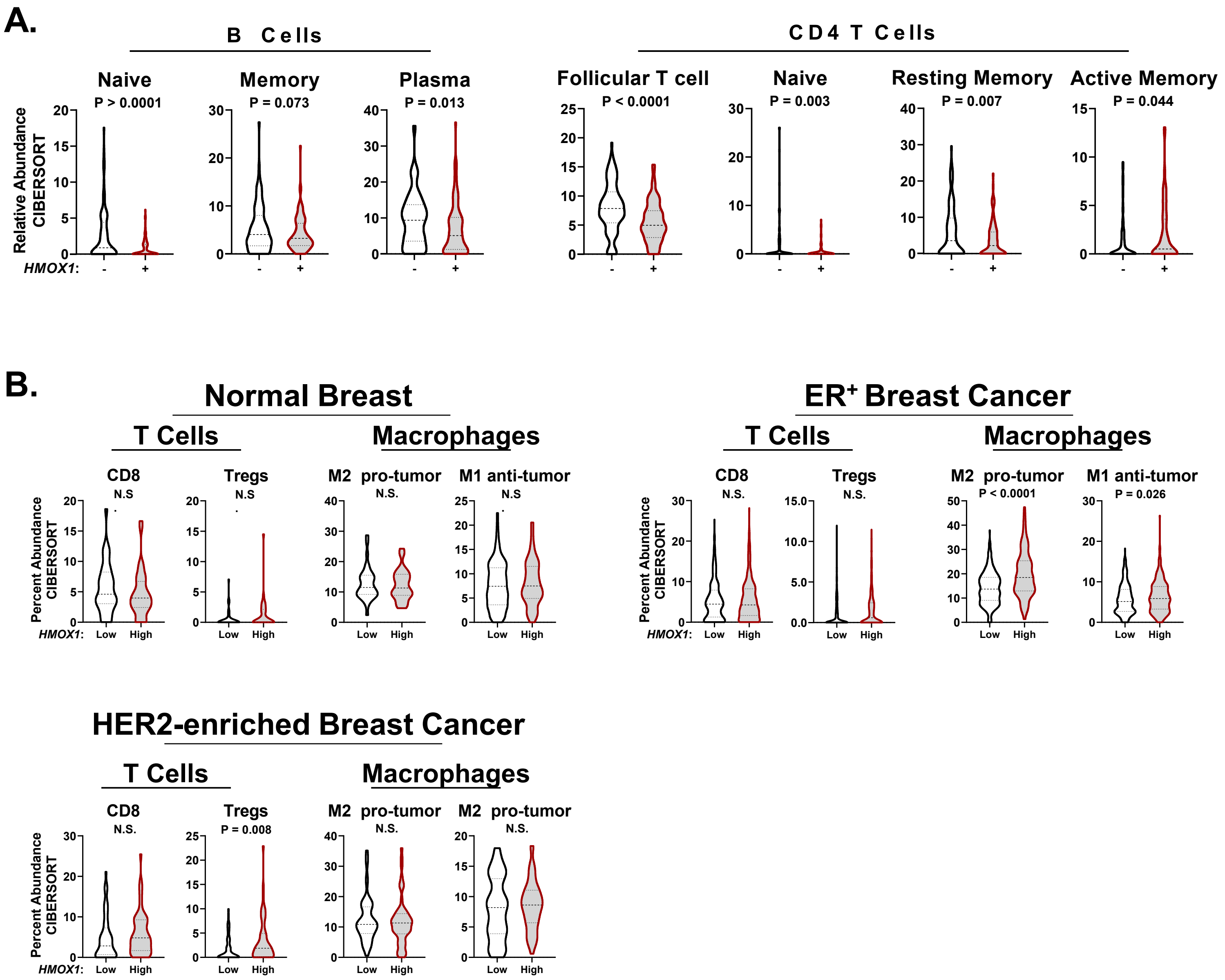

Supplementary Figure 2. *HMOX1* predicts altered immune cell abundance in breast tumor specimens but not normal breast tissues. Human breast cancer and normal breast specimens accessed using CBioPortal were analyzed using CIBERSORT. Specimens were stratified by *HMOX1* expression (low signifies *HMOX1* in the bottom quartile and high signifies *HMOX1* in the upper quartile) **A.** Shown is a violin plot for the predicted relative abundance of B cell and CD4 T cell populations in TNBC specimens (n = 101 per group, unpaired two-tailed T test). **B.** Violin plot of predicted abundance of cytotoxic and suppressive T cell and macrophage populations for normal breast samples (n = 36 per group) and ER+ (n = 285 per group) and HER2-enriched (n = 55 per group) breast cancer specimens (unpaired two-tailed T test).

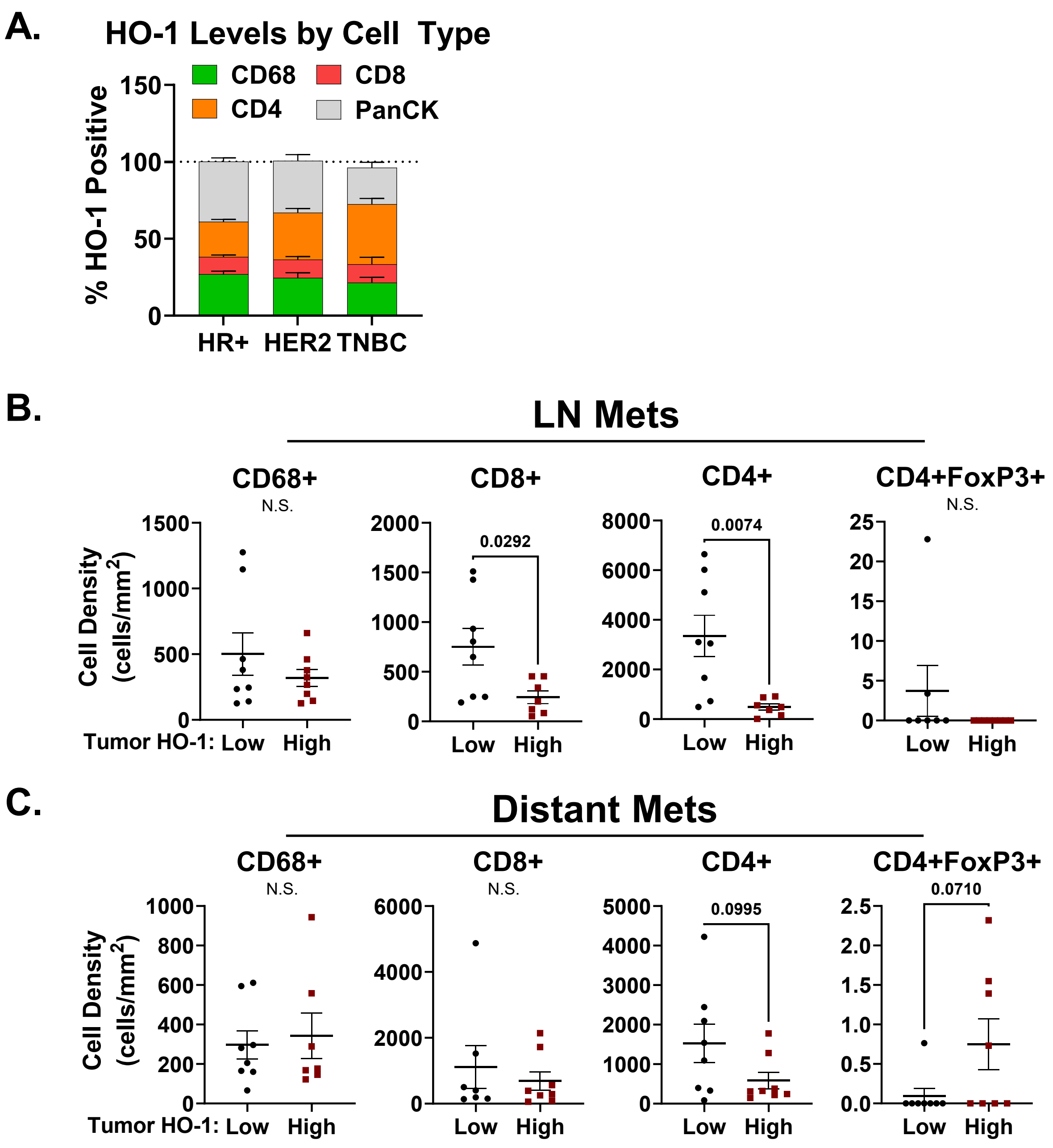

Supplementary Figure 3. Tumor-HO-1 levels correlate with CD68⁺ immune infiltrates across disease sites. HO-1 and immune cell markers were assessed in the CHTN_BrCaStg1 tissue microarray. **A.** Percent tumor cells (PanCK) and immune cells (CD4, CD8, or CD68) that were positive for HO-1 after stratification by subtype (mean ± SEM). **B-D.** Specimens were stratified by median expression of HO-1 in tumor cells. Presented is the density of immune cell populations in HO-1 low and high groups for lymph node metastasis (B, n = 7-8 per group), and distant metastasis specimens (D, n = 7-8 per group). For all graphs, shown is the mean ± SEM, unpaired two-tailed T test.

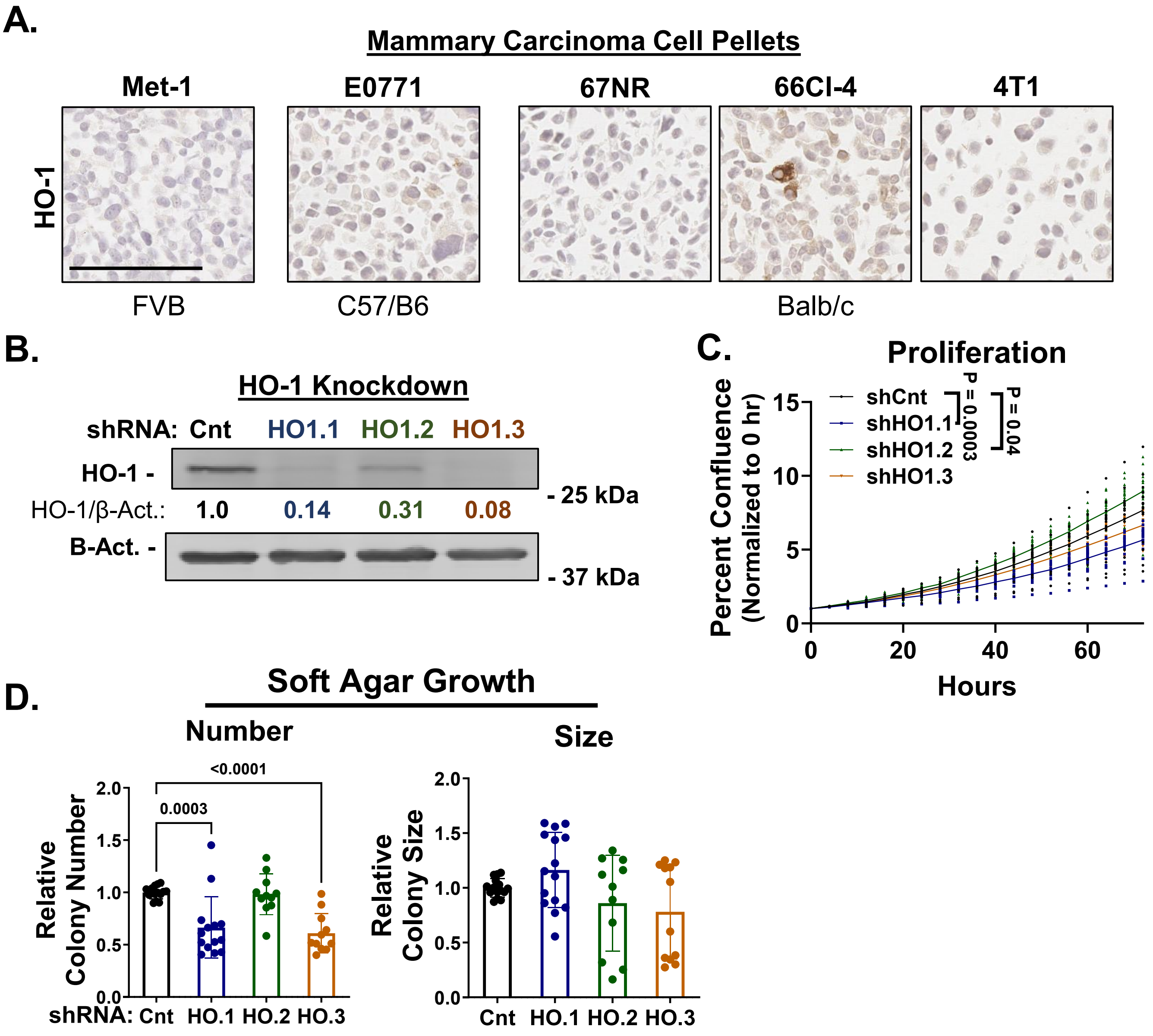

Supplementary Figure 4. Knockdown of *Hmox1* has a modest impact on tumor cell proliferation and colony growth. A. HO-1 expression via IHC in cell pellets from mouse mammary carcinoma cell lines (scale bar = 100 microns) **B.** *Hmox1* was depleted in 66Cl-4 mammary carcinoma cells using three different shRNA sequences (shHO.1, shHO.2, or shHO.3). Shown is a representative western blot demonstrating decreased expression of HO-1 in knockdown cells compared to those harboring shCnt. Quantification for relative protein abundance after normalizing to the loading control β-actin was conducted using ImageJ software. **C.** Percent confluence of 66Cl-4 shCnt, shHO.1, shHO.2, and shHO.3 cells observed every four hours using the IncuCyte Zoom imaging system. Data was normalized to 0 hours of growth (n = 18 per group, one-way ANOVA with Tukey's multiplex comparison test on area under the curve for each replicate). **D.** Relative number and size of colonies for 66Cl-4 shCnt, shHO.1, shHO.2, and shHO.3 cells grown in soft agar for 14 days as quantified by ImageJ Software (n = 12-15 per group, mean ± SEM, one-way ANOVA with Tukey's multiplex comparison test).

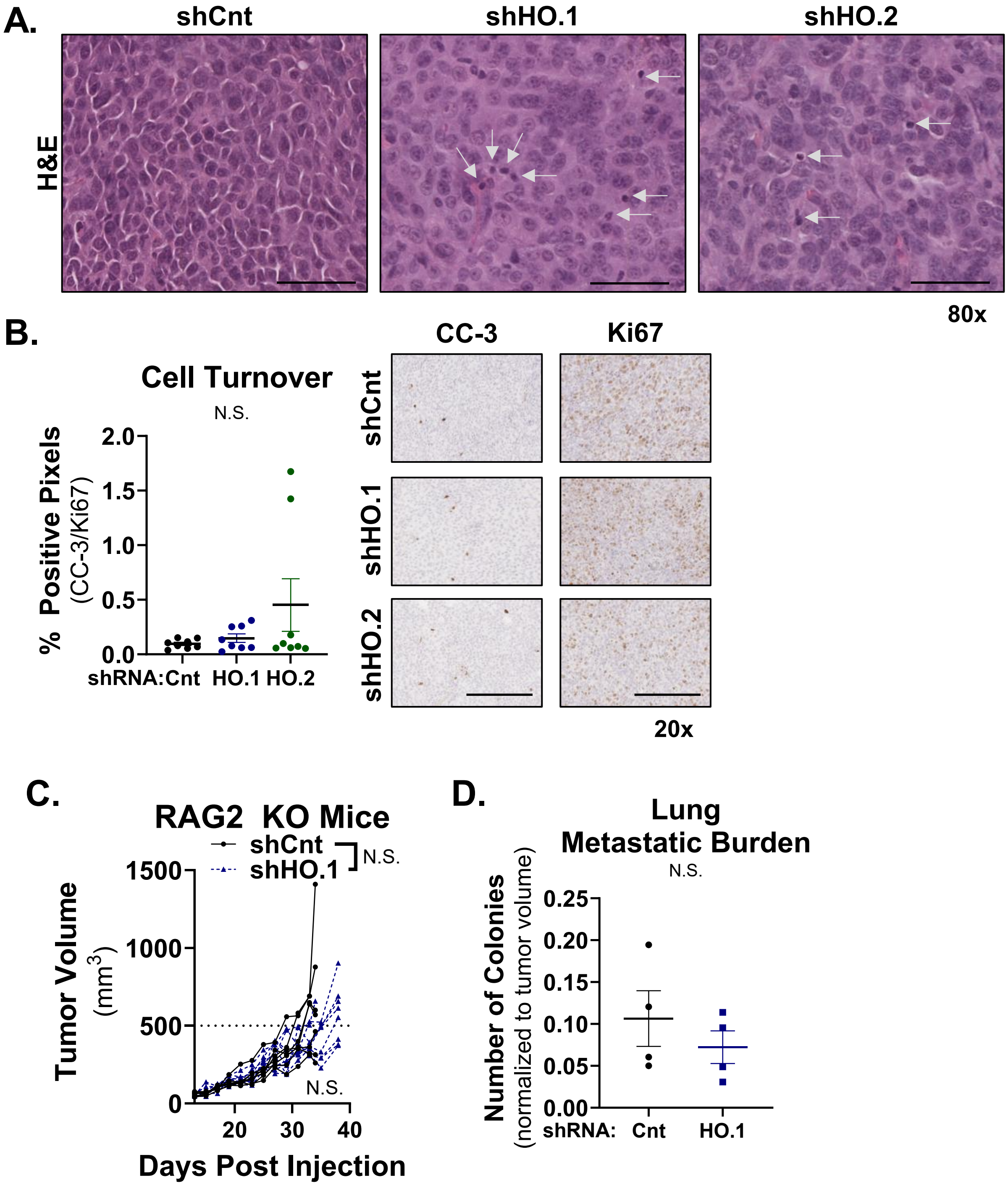

Supplementary Figure 5. Tumor-HO-1 inhibition alters tumor morphology in WT mice but does not impact tumor growth in immune compromised animals. A. Hematoxylin and eosin (H&E) staining conducted to observe histological changes in 66Cl-4 shCnt versus *Hmox1* knockdown tumors (scale bar = 50 microns, arrows point to small round cells that may be immune cells). **B.** Left: Growth index (percent positive pixels for cleaved caspase-3 divided by the percent positive pixels for Ki67) was quantified for each tumor using ImageScope Software (mean ± SEM, one-way ANOVA with Dunnett's multiplex comparison test). Right: Representative images (scale bar = 200 microns). **C-D.** 66Cl-4 shCnt or shHO.1 mammary tumor growth after bilateral injection in BALB/cJ RAG2 KO/IL2Rgc KO/SIRPa(NOD) mice (C, n = 8 per group, total mice n = 8, mean ± SEM, one-way ANOVA with Dunnett's multiple comparison test on area under the curve for each tumor). Lung metastatic burden was also determined by plating whole lung digests and observing colony outgrowth of tumor cells after antibiotic selection (D, n = 4 per group, mean ± SEM, unpaired two-tailed T test).

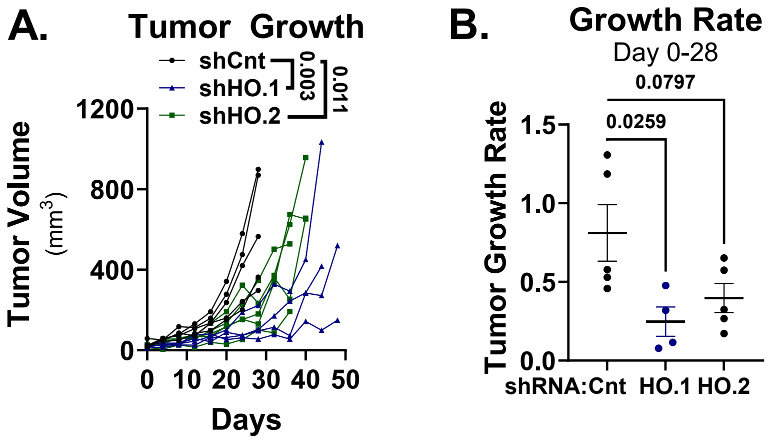

Supplementary Figure 6. Tumor-HO-1 inhibition decreases the tumor growth rate. A-B. Tumor volume over time (A, n = 4-5 per group, total mice n = 14, one-way Brown-Forsythe and Welch ANOVA with Dunnett's multiple comparison test of the average area under the curve of all tumors in each group) and tumor growth rate (B, mean ± SEM, one-way ANOVA with Dunnett's multiple comparison test) of unilaterally injected 66Cl-4 shCnt, shHO.1, and shHO.2 mammary tumors that were utilized for TIF isolation.

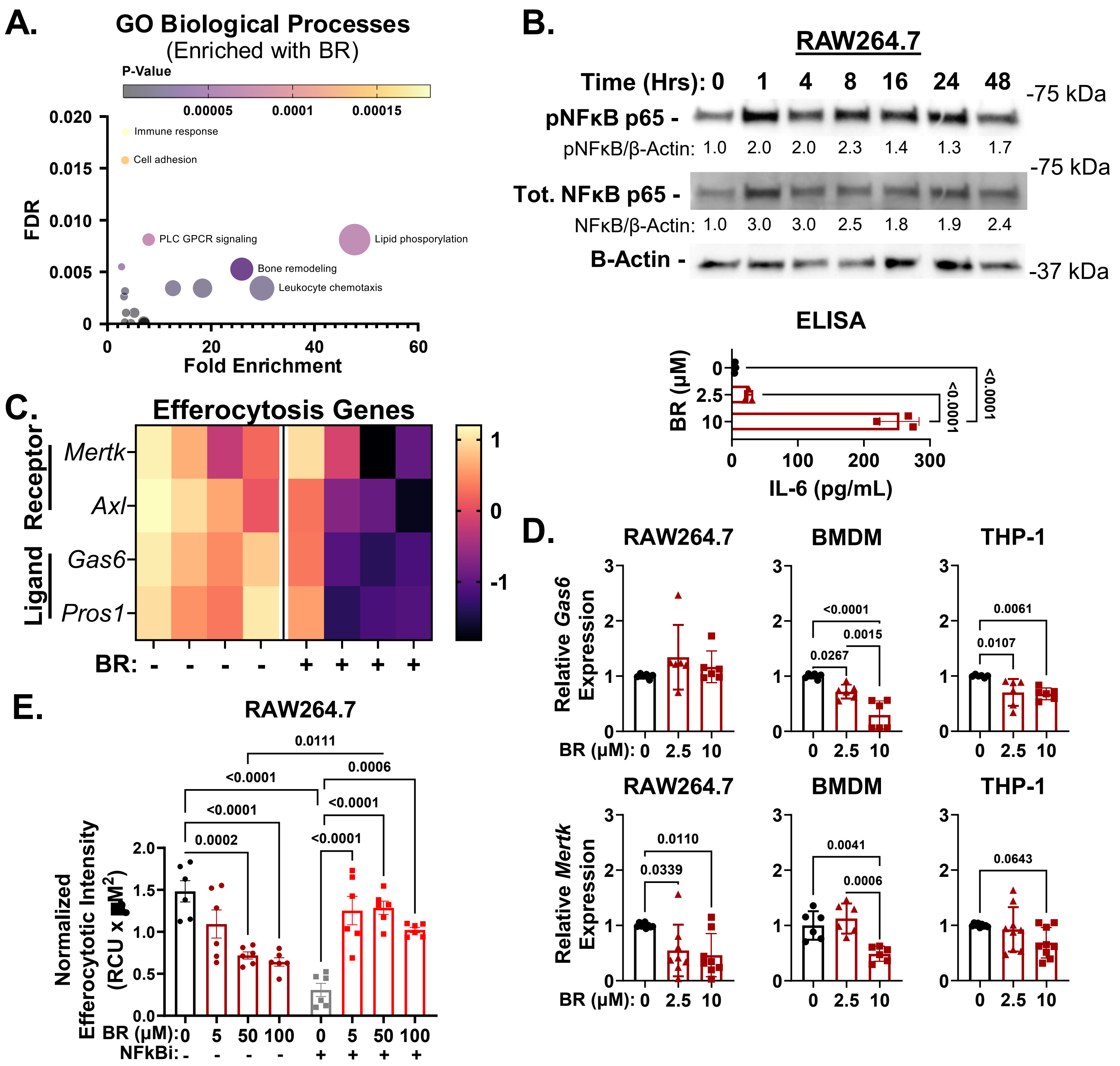

Supplementary Figure 7. Bilirubin alters genes and signaling pathways involved in normal macrophage function. A. Gene Set Enrichment Analysis was used to analyze top genes altered by 48 hours bilirubin (BR) treatment in bone marrow-derived macrophages (BMDM). Shown is the normalized enrichment score for the top altered pathways with a p-value < 0.05 (orange denotes pathways involved in immune modulation). **B.** Top: RAW264.7 cells were treated with 10 μM bilirubin for 0-48 hours and levels of phosphorylated NFκB p65 and total NFκB p65 were evaluated via western blot analysis. Expression of each was normalized to total gel protein in each well. Bottom: RAW264.7 cells were treated with 0-10 μM bilirubin and IL-6 was measured in the conditioned medium via ELISA (n = 3 per group, mean ± SD, one-way ANOVA with Tukey's multiplex comparison test). **C.** Heatmap for z-score normalized gene expression of efferocytosis receptors and ligands altered by bilirubin treatment in BMDM. Data was presented for each donor separately to visualize the baseline differences in gene expression between donors. **D.** RAW264.7, BMDM, or THP-1 cells were treated with 0-10 μM bilirubin for 48 hours and qRT-PCR was used to assess mRNA expression (n = 6-9 per group, mean ± SD, one-way ANOVA with Tukey's multiplex comparison test). **E.** RAW264.7 macrophages were cultured with pHrodo dyed dead tumor cells (66Cl-4) and treated with 0-100 μM BR and/or the NFκBi inhibitor (BAY 11-7082, 1.5 μM). Efferocytic intensity (red signal per μM^2) was observed at 24 hours using the IncuCyte Zoom imaging system (n = 6, mean ± SEM, one-way ANOVA with Tukey's multiplex comparison test).

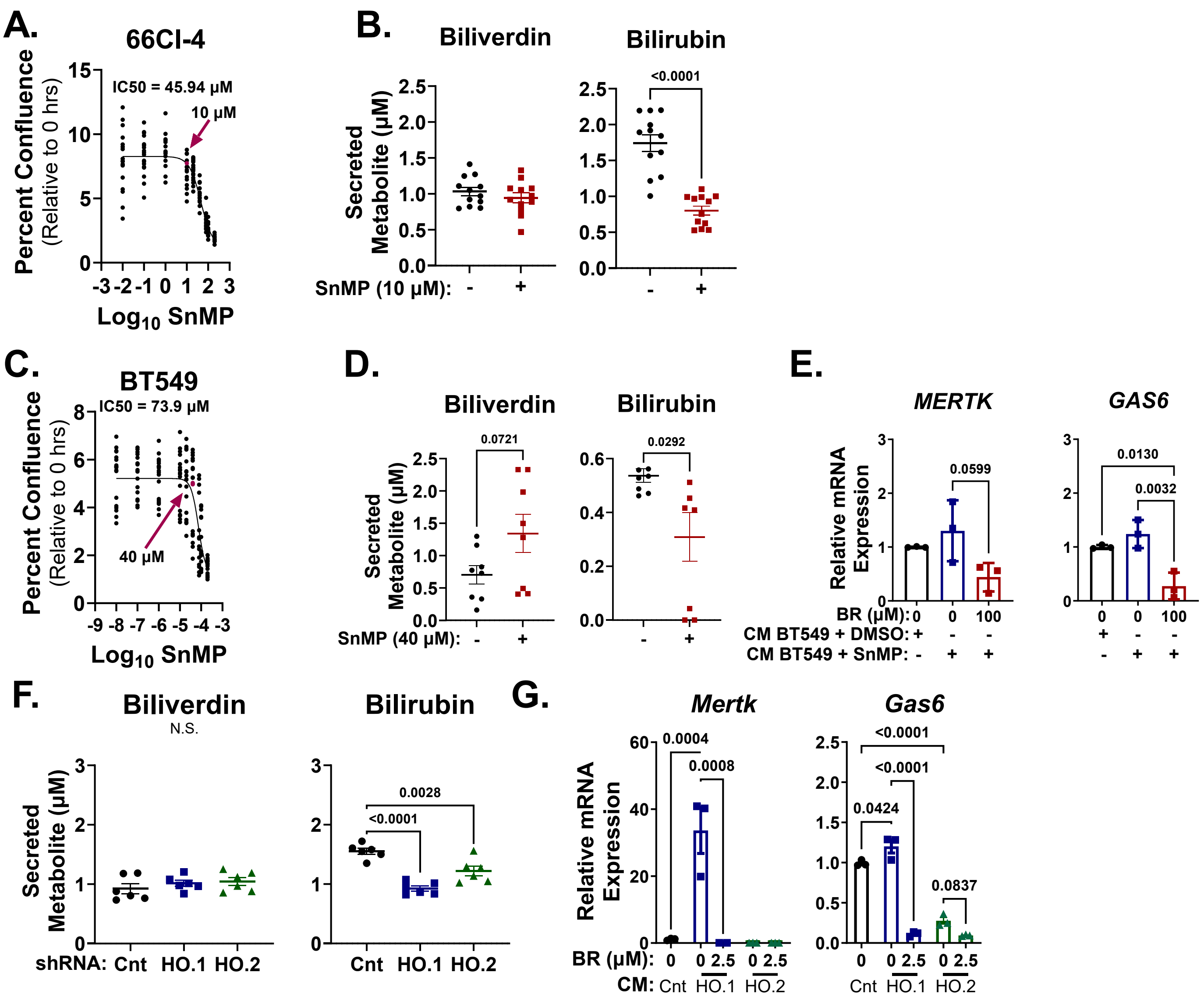

Supplementary Figure 8. Tumor produced bilirubin alters the expression of efferocytosis genes. **A.** 66Cl-4 cell were treated with increasing concentration of SnMP and percent confluence was determined at 72 hours treatment using the IncuCyte Zoom Imaging System and Software (A, n = 18 per group). **B.** Concentration of bilirubin and biliverdin in the conditioned medium of 66Cl-4 cells treated with 10 µM for 72 hours (n = 3 per group, mean ± SD, unpaired two-tailed T test). **C-D.** Experiments conducted in A-B were repeated in BT549 cells. **E.** THP-1 cells were differentiated into macrophages via PMA treatment, then cultured with conditioned medium from control (black) or SnMP-treated (blue) BT549 cells. Bilirubin was restored to conditioned medium from SnMP treated cells prior to placing on macrophages (red). After 48 hours treatment, mRNA expression of efferocytosis genes were determined via qRT-PCR (n = 3-6 per group, mean ± SEM, one-way ANOVA with Tukey's multiplex comparison test). **F.** Concentration of biliverdin and bilirubin in conditioned medium of 66Cl-4 shCnt or *Hmox1* knockdown cell lines after 72 hours growth (n = 6 per group, mean ± SEM, one-way ANOVA with Dunnett's multiplex comparison test). **G.** A similar experiment to E was conducted with conditioned medium from 66Cl-4 with genetic HO-1 depletion (n = 3 per group, mean ± SEM, one-way ANOVA with Tukey's multiplex comparison test).

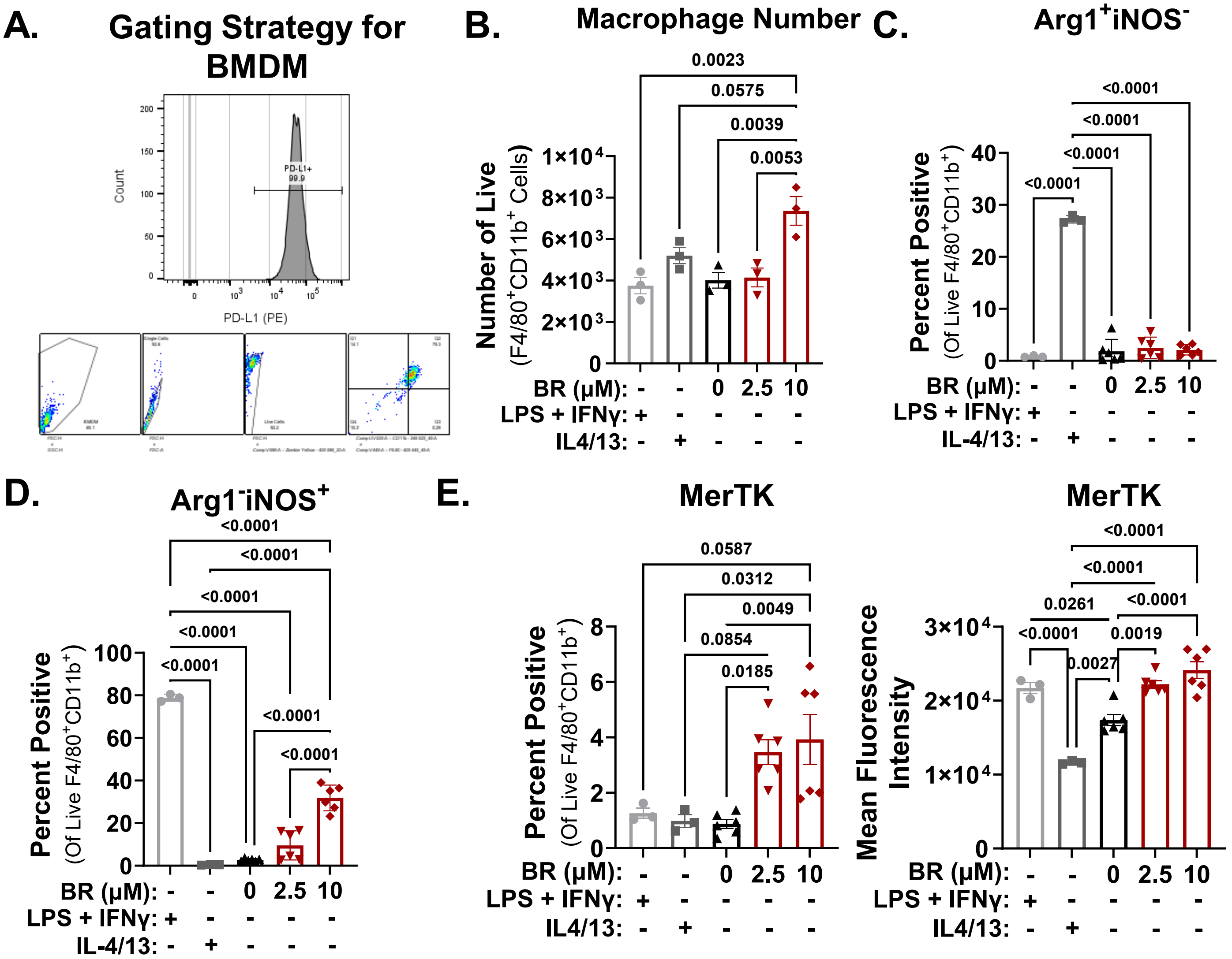

Supplementary Figure 9. Bilirubin treatment alters BMDM number and expression of inflammatory genes. A. Gating strategy for mouse macrophage flow cytometry panel (CD11b = BUV496; F4/80 = BV421; Arg1 = A488; iNOS = APC; CD206 = BV711; PD-L1 = PE; MerTK = A350; live/dead = Zombie Yellow). **B-E.** Bone marrow-derived macrophages treated with controls for pro-tumor (20 ng/mL IL-4 and IL-13) and anti-tumor (20 ng/mL IFNγ and 100 ng/mL LPS) polarization or 0-10 μM bilirubin for 48 hours. Shown is the number of live macrophages (cells positive for CD11b and F4/80) (B). Also shown are the percent Arg1^{+}iNOS^{-} positive, percent Arg1^{-}iNOS^{+} positive, and the percent positive and mean fluorescence intensity of MerTK in live macrophages (E) (for all n = 3-6 per group, mean ± SEM, one-way ANOVA with Tukey's multiplex comparison test).

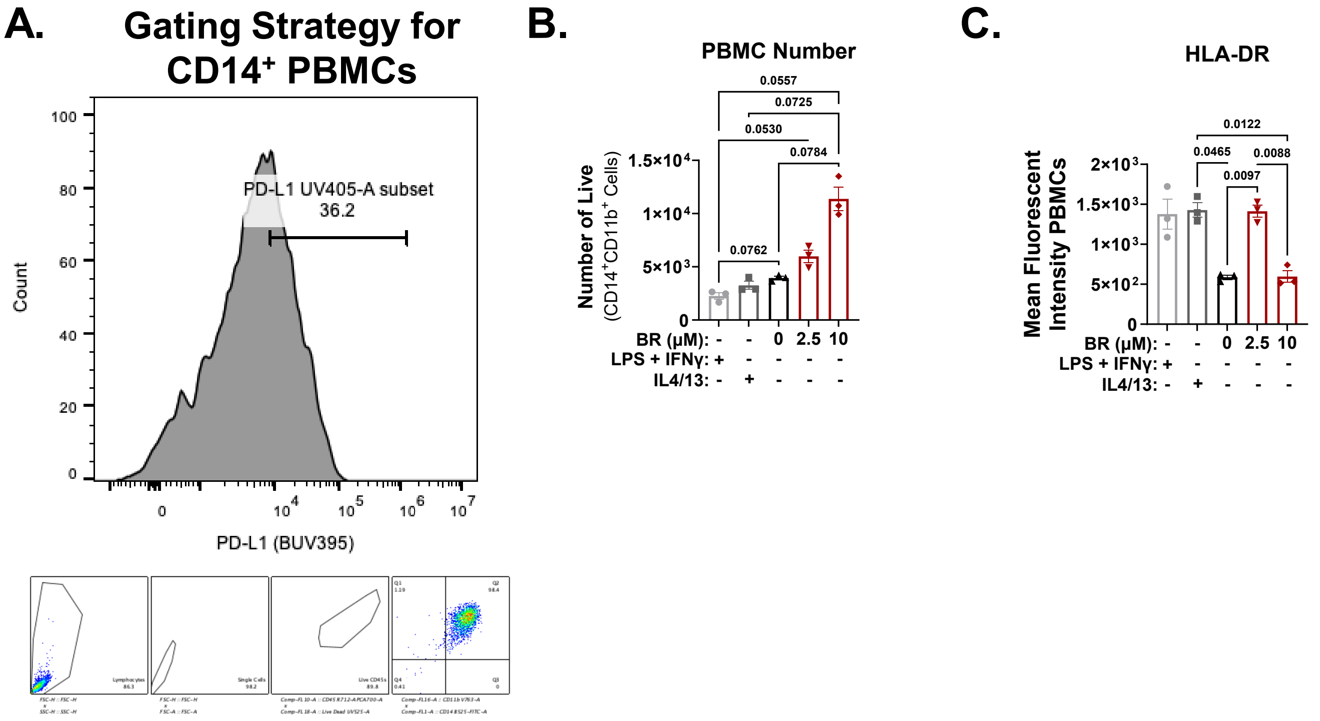

Supplementary Figure 10. Bilirubin treatment alters human MDM number and expression of inflammatory genes. A. Gating strategy for human macrophage flow cytometry panel (CD45 = AF700, CD14 = AF488, CD11b = BV785; CD206 = PE; PD-L1 = BUV395; HLA-DR = APC; live/dead = Zombie UV). **B-C.** Human CD14⁺ PMBCs treated with controls for pro-tumor (20 ng/mL IL-4 and IL-13) and anti-tumor (20 ng/mL IFNγ and 100 ng/mL LPS) polarization or 0-10 μM bilirubin for 48 hours. Shown is the number of live macrophages (cells positive for CD45, CD14, and CD11b) (B). Also shown is the mean fluorescence intensity of HLA-DR on live macrophages (C) (for all n = 3 per group, mean ± SEM, one-way ANOVA with Tukey's multiplex comparison test).

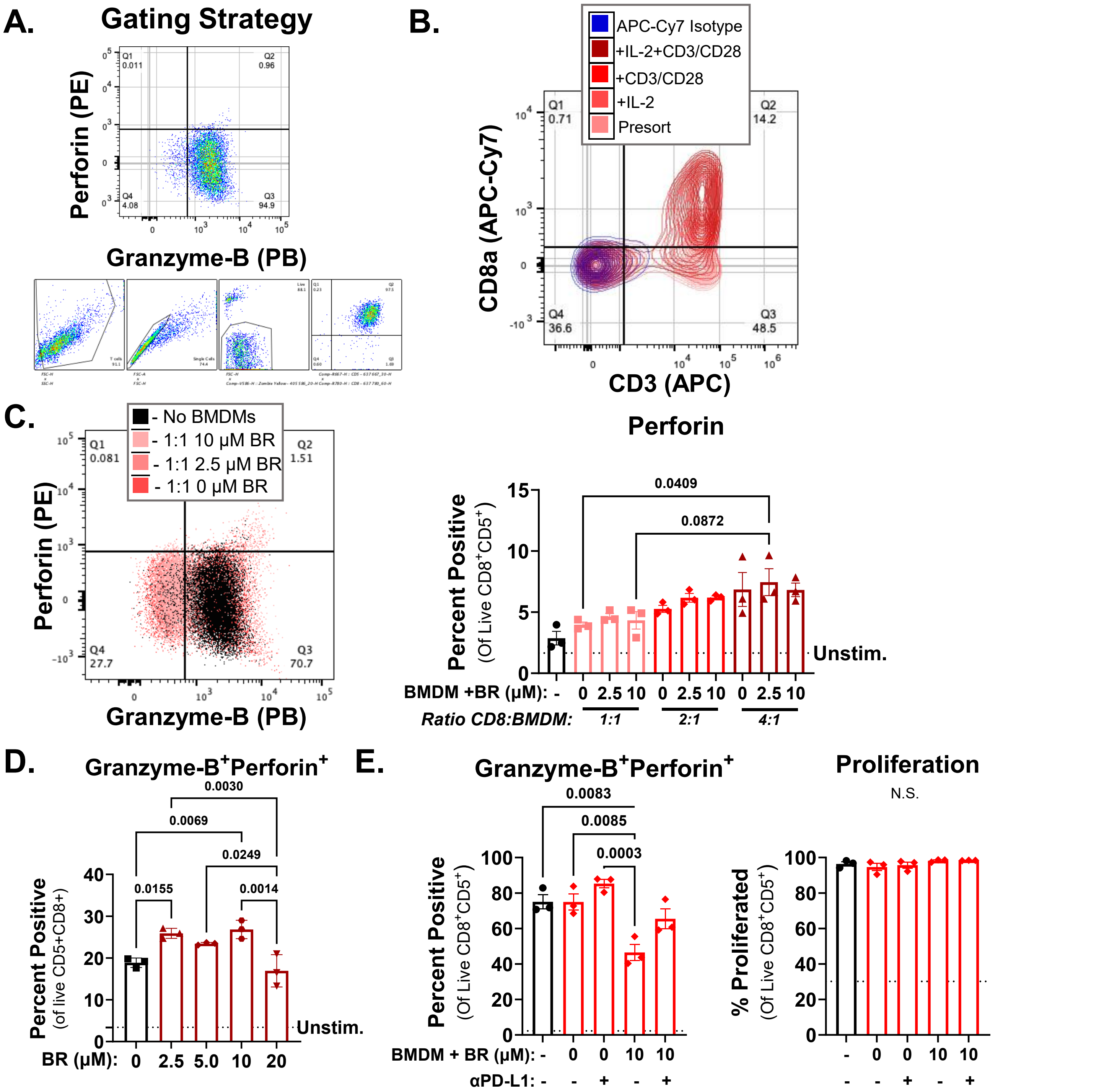

Supplementary Figure 11. Macrophage immune suppression is altered by bilirubin. A. Gating strategy for T cell flow cytometry panel (CD5 = APC; CD8 = APC-Cy7; Perforin = PE; Granzyme-B = FITC; live/dead = Zombie UV). **B.** CD8$^+$ splenocytes stained with CellTrace Blue and treated with T cell stimulating factors (CD28/CD3 beads and IL-2). After 72 hours co-culture, expression of CD8α (APC-Cy7) was compared between splenocytes stained with an APC-Cy7 isotype control antibody (blue) versus the full T cell activation panel (red). Shown are the percent live CD8$^+$CD5$^+$ splenocytes positive for **C.** Perforin after co-culture with bilirubin treated bone marrow-derive macrophages for 72 hours (n = 3 per group, mean ± SEM, one-way ANOVA with Tukey's multiplex comparison test). **D.** In a similar experiment splenocytes cultured alone were treated with 0-20 μM bilirubin in the presence of stimulating CD3/CD28 and IL-2 for 72 hours. Shown is the percent live CD8$^+$CD5$^+$ splenocytes positive for Perforin and Granzyme-B (n = 3 per group, mean ± SEM, one-way ANOVA with Tukey's multiplex comparison test). **E.** BMDM were cultured with 0-10 μM BR for 48 hours after which they were co-cultured at varying ratios with CD8$^+$ splenocytes stained with CellTrace Blue and treated with T cell stimulating factors (CD28/CD3 dynabeads and IL-2) and IgG control or αPD-L1. After 72 hours co-culture, splenocytes were harvested and expression of cytotoxic molecules (left) and CellTrace Blue (right) were observed via flow in CD8$^+$CD5$^+$ cells (n = 3, one-way ANOVA with Tukey's multiple comparison test).

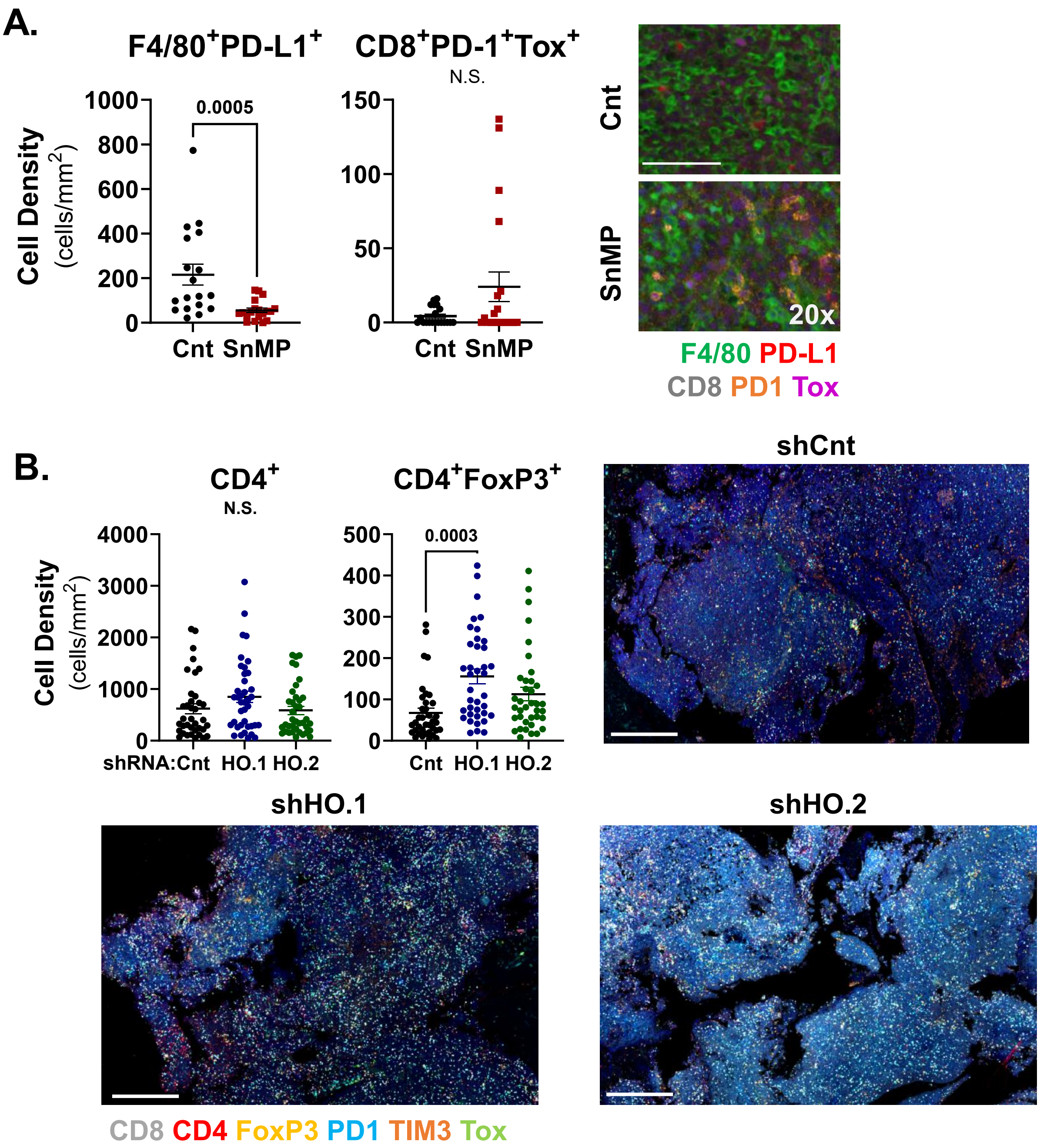

Supplementary Figure 12. HO-1 inhibition alters T cell and macrophage populations. A. Primary 66Cl-4 tumors from mice treated with SnMP (25 mg/kg daily for 14 days, n = 5 per group) were observed by multiplex IF. Shown is the cell density of suppressive macrophages (F4/80⁺PD-L1⁺) and exhausted T cells (CD8⁺PD-1⁺Tox/Tox2⁺) for 5 fields of view per tumor (mean ± SEM, unpaired two-tailed T test) and representative images (F4/80 = green, PD-L1 = yellow, CD8 = grey, PD-1 = orange, Tox/Tox2 = magenta, scale bar = 100 microns). **B.** Suppressive T cells were observed in 66Cl-4 shCnt, shHO.1, and shHO.2 mammary tumors using a custom multispectral fluorescence panel (T cell: CD8/grey, CD4/red, FoxP3/yellow, PD-1/cyan, TIM3/orange, TOX1/2/green). Shown is the quantification for the density of regulatory T cells (for all mean ± SEM, one-way ANOVA with Tukey's multiple comparison test). Zoomed out representative images are also shown, scale bar = 1 mm.

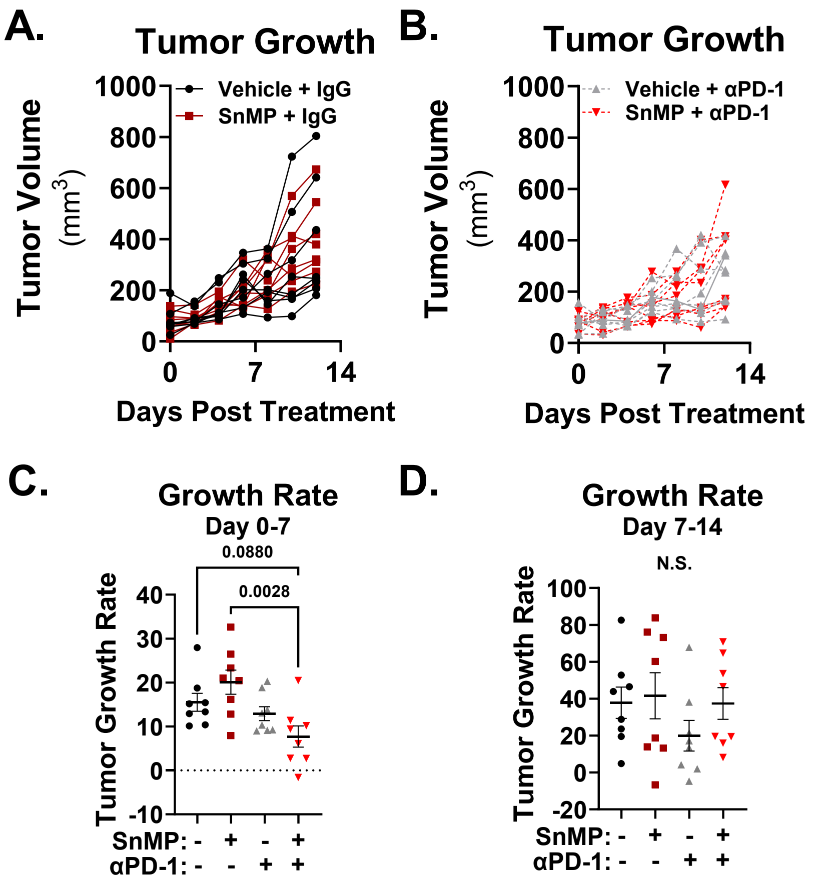

Supplementary Figure 13. HO-1 pharmacologic inhibition temporarily decreases tumor growth rate in combination with immunotherapy. A-D. Individual tumor growth curves (A-B) and tumor growth rate (from day 0-7 (C) and 7-14 (D)) calculated for bilaterally injected 66Cl-4 mammary tumors treated with SnMP or αPD-1 (C, n = 8 per group, total mice n = 16, mean ± SEM, one-way ANOVA with Tukey's multiplex comparison test).

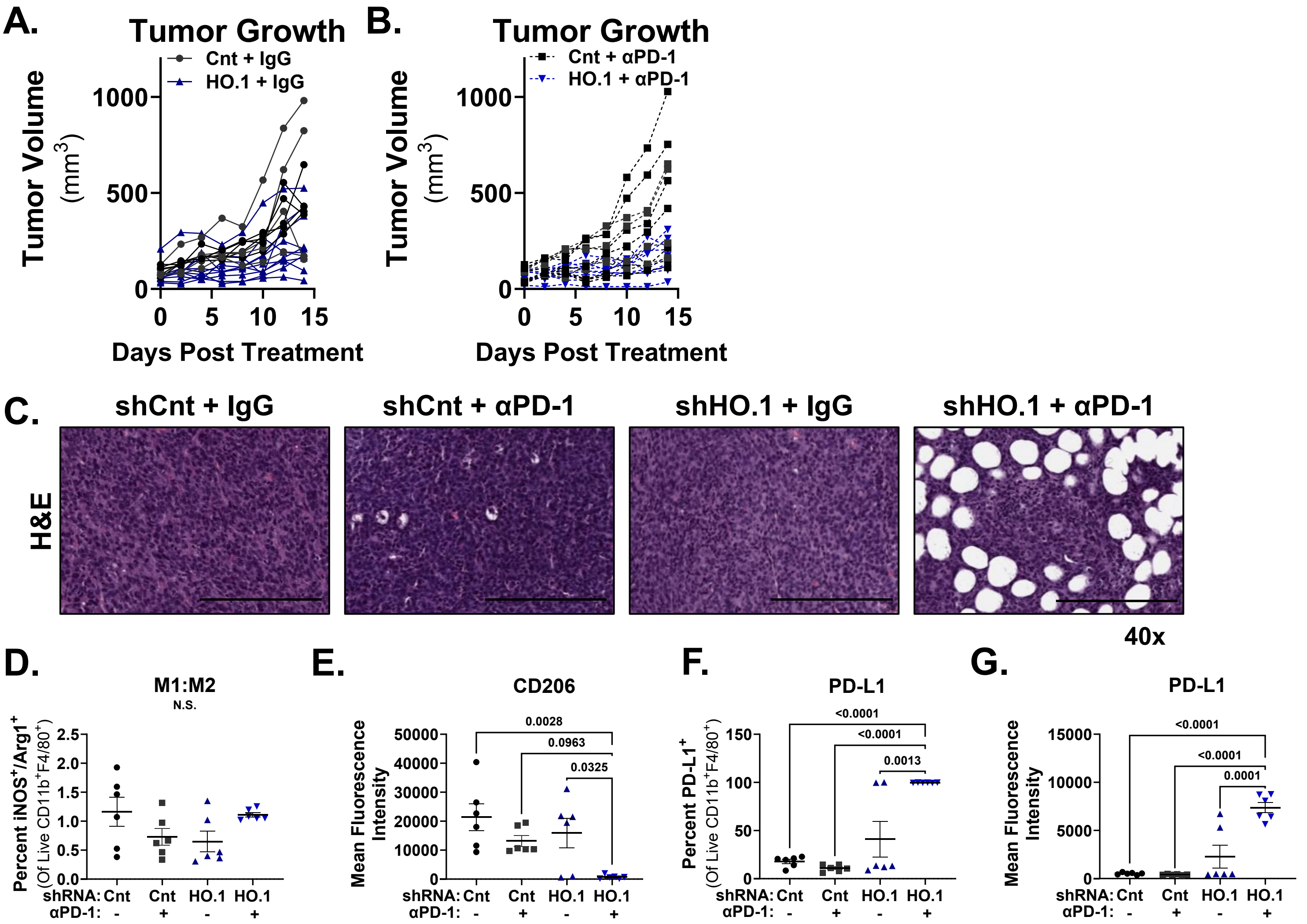

Supplementary Figure 14. HO-1 inhibition alters tumor growth rate and macrophage suppressive markers in combination with immunotherapy. A-B. Individual tumor growth curves of 66Cl-4 shCnt (A) and shHO.1 (B) tumors from mice treated with IgG control or αPD-1. **C.** Representative H&E images of 66Cl-4 shCnt and shHO.1 tumors from mice treated with and without αPD-1 (scale bar = 200 microns). **D-G**. Flow cytometry for macrophage markers (CD11b = BUV496; F4/80 = BV421; Arg1 = A488; iNOS = APC; CD206 = BV711; PD-L1 = PE; live/dead = Zombie Yellow) was conducted on 66Cl-4 shCnt or shHO.1 mammary tumors treated with αPD-1 or IgG control for 14 days. Shown are the percent macrophages (live $CD11b^+F4/80^+$ cells) positive for iNOS divided by those positive for Arg1 to generate an M1:M2 macrophage score, the mean fluorescence intensity for CD206 on macrophages (D) and the percent macrophages and mean fluorescence intensity for PD-L1 (E-G, n = 6 per group, mean ± SEM, one-way ANOVA with Tukey's multiplex comparison test).

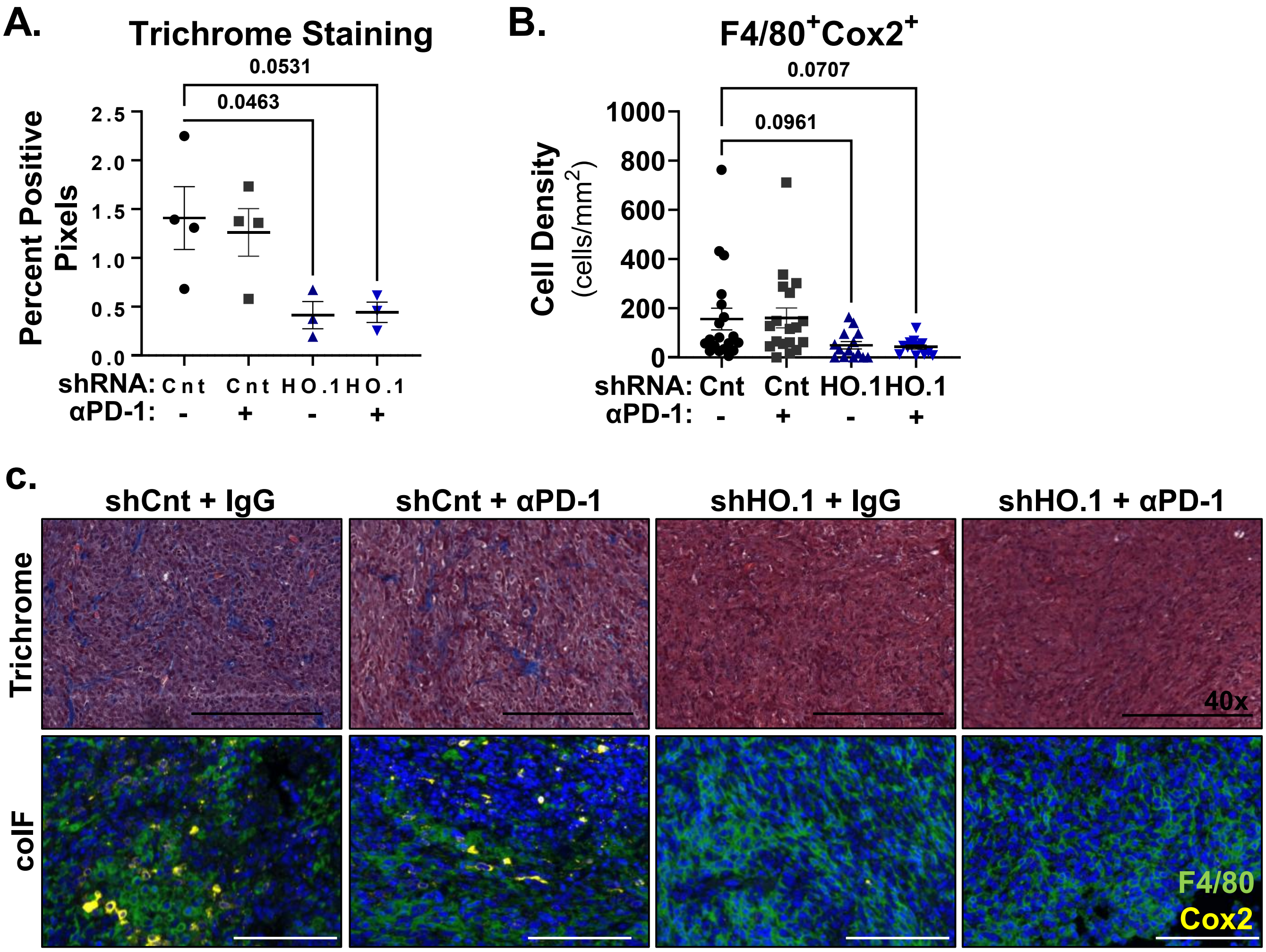

Supplementary Figure 15. HO-1 inhibition alters tumor extracellular matrix composition. A. Masson's trichrome staining was conducted in tumors from Figure 6C. The percent blue positive pixels (collagen) were quantified and shown (mean ± SEM, one-way ANOVA with Dunnett's multiple comparison test). **B.** Co-immunofluorescence for F4/80 and Cox2 was conducted in the same tissues. Shown is the density of dual positive cells (mean ± SEM, one-way ANOVA with Dunnett's multiple comparison test). **C.** Representative images for trichrome staining and coIF are shown (scale bar = 200 microns for trichrome and 100 microns for coIF, green = F4/80, yellow = COX2).

A.

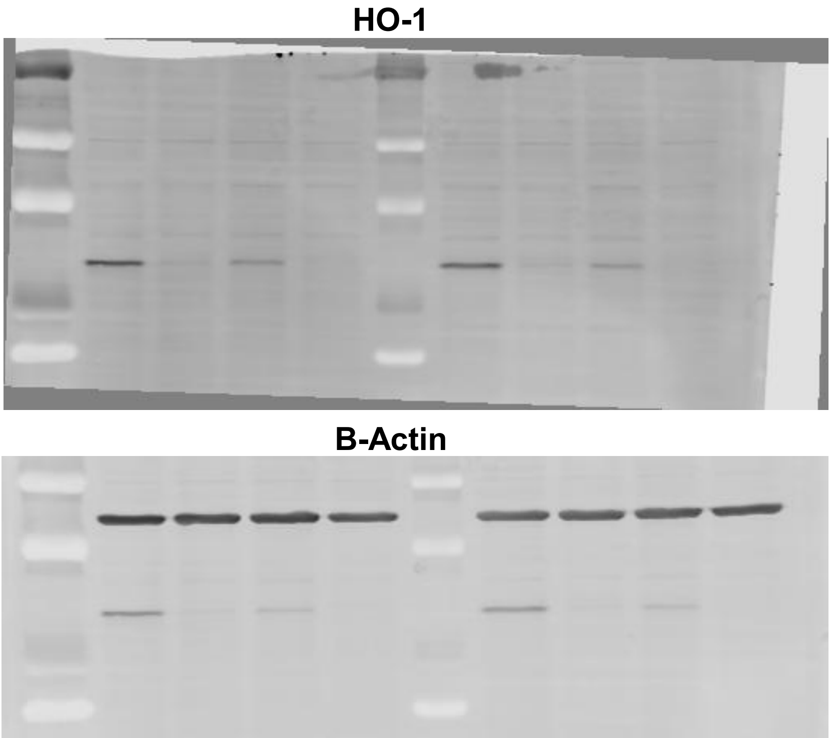

B.

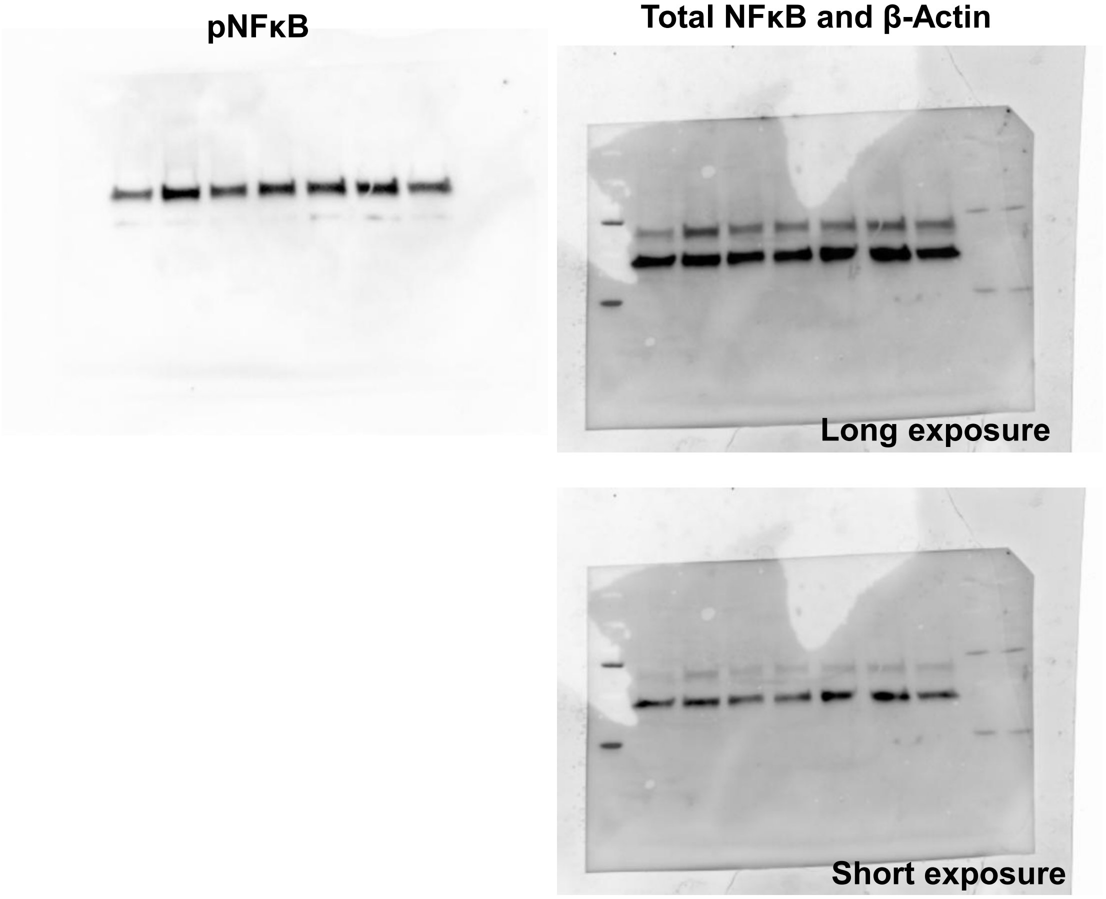

Supplementary Figure 16. A. Uncropped and unedited western blot gels for Supplementary Figure 4B. **B.** Uncropped and unedited western blot gels for Supplementary Figure 7B.

Supplementary Table 1. Genes altered in bone marrow-derived macrophages treated with bilirubin for 48 hours. Shown are the top 50 upregulated and downregulated genes.

	Z-Score Normalized Gene Expression							
**Gene**	**BMDM_1_2.5 uM BR**	**BMDM_3_2.5 uM BR**	**BMDM_4_2.5 uM BR**	**BMDM_2_2.5 uM BR**	**BMDM_1_0 uM BR**	**BMDM_4_0 uM BR**	**BMDM_3_0 uM BR**	**BMDM_2_0 uM BR**
Cfb	0.81	1.07	0.89	0.72	-0.64	-0.35	-0.92	-1.59
Mmp9	0.94	1.20	0.99	0.51	-0.64	-1.11	-0.97	-0.92
Cxcl3	0.22	1.36	1.10	0.78	-0.85	-0.63	-1.31	-0.67
Saa3	0.82	1.17	0.95	0.77	-0.96	-0.86	-0.97	-0.93
Lcn2	0.86	1.16	1.00	0.65	-0.71	-0.93	-0.84	-1.20
Hp	0.86	1.03	1.04	0.80	-0.91	-0.98	-0.96	-0.88
Marco	1.19	1.10	1.00	0.30	-0.79	-0.89	-0.96	-0.96
Il1a	0.91	1.19	0.94	0.48	-0.88	-0.39	-1.42	-0.82
Cxcl1	0.59	1.29	1.07	0.68	-1.07	-0.67	-0.86	-1.02
Ccl5	0.74	1.17	0.92	0.89	-0.90	-0.97	-0.93	-0.91
Acod1	0.82	1.22	1.02	0.62	-0.89	-0.89	-0.90	-1.00
Fpr1	0.92	1.26	0.81	0.60	-0.52	-0.83	-1.34	-0.89
Clmp	1.04	1.24	1.04	0.15	-1.16	-0.51	-1.00	-0.81
C1s1	0.89	0.99	1.00	0.82	-1.04	-1.14	-0.85	-0.66
C3	1.11	1.17	0.85	0.53	-0.88	-0.88	-0.90	-1.02
Il1b	0.69	1.51	0.98	0.22	-1.00	-0.36	-0.88	-1.16
Fpr2	0.97	1.27	1.02	0.26	-0.47	-0.98	-1.11	-0.96
Il1f9	0.15	1.19	0.85	1.25	-0.79	-1.35	-0.59	-0.70
Gm5424	0.80	1.12	1.05	0.48	-0.51	-1.61	-0.77	-0.57
Slpi	0.95	1.16	0.93	0.64	-0.69	-0.91	-1.07	-1.02
Pilrb1	0.85	1.07	1.03	0.25	-1.13	0.15	-0.78	-1.44
Slc39a4	1.05	1.15	0.83	0.67	-0.95	-0.77	-0.91	-1.06
Mmp14	0.99	1.29	1.00	0.31	-0.83	-0.86	-0.87	-1.03
Cxcl2	0.40	1.34	1.07	0.67	-0.63	-0.77	-0.67	-1.41
Cdc42ep2	0.68	1.28	1.22	0.32	-0.70	-0.78	-0.72	-1.29
Vcan	0.81	1.22	0.83	0.73	-1.29	-0.97	-0.92	-0.43
H2-M2	0.28	1.32	0.94	0.85	-1.20	-1.31	-0.54	-0.35
Flrt3	0.33	1.06	1.17	0.68	-1.76	-0.76	-0.25	-0.46
Ass1	0.87	1.12	1.01	0.72	-0.88	-0.93	-1.00	-0.91
Aoah	1.28	1.14	0.77	0.43	-0.96	-0.74	-1.00	-0.91
Gpr68	0.51	1.04	0.80	1.10	-1.14	-1.19	-1.05	-0.06
Slc6a12	0.63	1.30	0.86	0.79	-1.17	-1.20	-0.60	-0.62
Clec4e	0.74	1.33	1.09	0.42	-0.90	-0.69	-0.87	-1.12
Serinc2	0.54	1.30	0.73	1.00	-1.11	-0.97	-1.08	-0.40
Adh7	0.26	1.05	1.07	1.20	-0.83	-1.05	-0.94	-0.76
Calr3	0.51	0.97	1.13	1.00	-1.32	-0.88	-0.79	-0.62
Gm34643	0.91	1.02	0.87	0.73	-1.62	-0.63	-0.64	-0.64
Dcstamp	0.23	1.03	0.73	0.99	-2.01	-0.28	-0.36	-0.33
Traf1	1.10	1.27	0.99	0.16	-0.75	-1.02	-0.78	-0.98
Pilrb2	1.32	0.91	1.04	0.28	-0.99	-0.68	-0.84	-1.05
Arg2	1.40	0.81	0.54	0.60	-0.87	-1.42	-0.97	-0.10
Uchl1	0.78	1.38	1.04	0.37	-1.02	-0.63	-1.00	-0.91
Fgr	1.04	1.15	1.08	0.27	-0.83	-0.78	-0.67	-1.27
Arl5c	1.14	1.17	0.79	0.06	-1.67	-0.17	-0.53	-0.79
Mefv	0.63	1.19	1.25	0.53	-0.90	-0.72	-0.78	-1.20
Ptges	0.40	1.60	1.06	0.38	-1.03	-0.87	-0.69	-0.84
Sdc1	0.15	1.44	1.26	0.46	-1.16	-0.83	-0.34	-0.98
Tarm1	0.07	1.33	1.07	0.95	-0.99	-1.06	-0.95	-0.41
Ednrb	0.66	0.90	0.85	1.19	-0.75	-1.24	-1.10	-0.51
Slc40a1	-0.89	-1.31	-1.29	0.38	0.99	0.52	0.54	1.05
Enpp1	-0.22	-1.17	-1.02	-1.12	0.85	1.17	0.59	0.92
Ctla2b	-0.18	-1.49	-0.91	-0.89	1.04	0.81	0.61	1.01
Galnt3	-0.34	-0.44	-1.51	-0.92	1.65	0.52	0.66	0.37
Thsd1	-0.11	-1.13	-0.88	-1.30	0.96	1.19	0.85	0.43
Epas1	0.36	-1.38	-1.20	-0.94	1.06	0.74	0.86	0.48
Hdac9	-0.43	-0.86	-0.47	-1.65	0.93	1.08	0.94	0.45
Zfp618	-0.97	-0.82	-1.36	-0.40	1.08	1.16	0.56	0.75
Gm42793	-0.79	-0.61	-1.81	0.08	0.90	1.11	0.40	0.72
Arap2	-0.82	-1.26	-1.06	-0.39	1.29	0.88	0.87	0.50
Folr1	-0.98	-1.37	0.11	-1.13	0.86	0.64	1.00	0.86
Fzd7	-0.73	-1.34	-1.22	-0.24	0.84	0.93	0.93	0.83
Gm38248	-0.02	-0.93	-1.10	-1.20	1.59	0.72	0.47	0.45
Zbtb16	-0.24	-1.12	-1.28	-0.78	0.22	1.10	1.05	1.04
Pmepa1	-1.27	-0.69	-1.34	-0.03	0.91	0.33	1.03	1.06
Etv4	-0.84	-1.32	-0.32	-1.08	1.11	0.97	0.78	0.70
Jag2	-0.13	-0.95	-1.21	-0.84	1.49	1.04	0.76	-0.17
Plau	-1.80	-0.80	-0.53	-0.14	0.58	0.91	1.06	0.72
Flt1	-1.32	-0.79	-0.45	-0.99	0.55	0.89	1.31	0.79
Ndrg2	-0.78	-0.88	-0.84	-0.98	1.54	0.62	1.06	0.26
Disc1	0.15	-0.98	-0.84	-1.50	1.40	0.42	0.63	0.73
Rnase4	0.38	-1.35	-0.99	-1.19	0.86	1.02	0.71	0.57
Qpct	-0.32	-0.96	-0.98	-1.29	1.19	0.91	0.90	0.54
Ccl7	-1.71	-0.17	-0.24	-0.89	0.12	1.42	0.96	0.51
Gm1673	0.47	-0.81	-1.22	-1.44	1.08	0.91	0.34	0.67
Apoe	-0.13	-1.28	-1.08	-1.00	1.15	0.92	0.78	0.63
Smagp	0.38	-1.59	-0.77	-0.88	1.21	1.14	0.45	0.07
F13a1	0.26	-0.68	-1.55	-1.00	0.61	0.98	0.05	1.32
Sox4	-1.27	-0.97	-1.11	-0.12	0.60	0.94	1.20	0.72
Cx3cr1	-0.52	-1.18	-0.98	-0.94	1.22	1.01	0.87	0.51
Gas6	0.34	-1.36	-1.12	-1.04	1.09	0.87	0.46	0.76
Cd28	-0.18	-0.74	-1.81	-0.39	1.18	0.58	0.28	1.07
Rasgrp3	-0.61	-1.21	-1.17	-0.63	0.95	0.74	1.16	0.77
Gdf15	-0.66	-1.34	-1.34	0.03	0.83	0.41	1.04	1.03
Ang	-0.63	-0.54	-1.58	-0.70	1.15	1.13	0.73	0.45
Gm33370	-1.06	-0.59	-0.56	-1.23	1.52	0.95	0.41	0.55
Ighm	0.16	-1.36	-1.27	-0.80	1.14	0.75	0.52	0.86
Pde1c	-0.44	-1.31	-1.15	-0.64	1.21	0.98	0.58	0.77
Cd244a	-0.79	-0.41	-0.44	-1.59	1.57	0.57	0.30	0.79
Thbs1	-0.75	-0.96	-1.41	-0.39	0.79	0.47	1.03	1.22
Adgrg3	-0.77	-1.12	-1.25	-0.32	0.82	0.49	0.74	1.42
Ctla2a	-0.52	-1.97	-0.64	0.04	0.68	0.63	0.79	0.98
Chn2	0.10	-1.88	-0.09	-0.98	1.10	1.00	0.46	0.29
Serpine2	-1.22	-0.41	-0.68	-1.24	1.08	0.96	1.01	0.50
Tpbgl	-1.03	-1.61	-0.60	-0.15	0.76	0.83	0.74	1.07
Ch25h	-0.21	-0.76	-0.90	-1.51	1.14	1.17	0.66	0.41
Ccnd2	-0.57	-0.58	-1.81	-0.34	1.03	0.43	1.04	0.80
Fcrls	0.90	-1.38	-0.98	-1.17	0.96	0.70	0.29	0.68
Fosb	-0.55	-1.16	-0.78	-1.15	0.70	1.14	0.81	0.98
Gm49391	0.22	-0.53	-0.99	-1.79	0.78	0.91	0.54	0.86

Supplementary Table 2. Genes altered in bone marrow-derived macrophages tr
for 48 hours. Shown are genes altered with an adjusted P-value of less than 0.05
expression was determined using the mouse identifier as a covariate to normalize
differences between BMDM isolated from different animals.

Z-Score Normalized Gene Expression						
**Gene**	**logFC**	**AveExpr**	**t**	**P.Value**	**adj.P.Val**	**B**
Cfb	-6.038887	0.3256374	-6.864993	2.687E-05	0.0019551	1.9342837
Mmp9	-5.701255	0.8516281	-5.703471	0.0001366	0.0059058	1.0498775
Cxcl3	-5.442187	-1.36863	-4.600024	0.0007615	0.0196537	-0.774655
Saa3	-5.296933	4.321236	-11.54423	1.702E-07	7.804E-05	7.6789899
Lcn2	-5.131495	-1.030731	-9.373252	1.388E-06	0.0003189	2.8018421
Hp	-4.160576	1.7184697	-15.71043	6.847E-09	2.006E-05	8.3855656
Marco	-3.636901	4.6725342	-7.223659	1.685E-05	0.0014303	3.3089775
Il1a	-3.62633	-0.544818	-7.40108	1.345E-05	0.001203	2.3532081
Cxcl1	-3.478301	0.9744399	-7.726853	8.983E-06	0.0009397	3.4544991
Ccl5	-3.382054	1.8405612	-12.95723	5.171E-08	4.039E-05	7.6479986
Acod1	-3.377675	5.4752688	-13.73453	2.817E-08	3.885E-05	9.6694362
Fpr1	-3.359793	1.2974026	-8.240832	4.867E-06	0.0006555	4.0769074
Clmp	-3.25217	-0.876363	-5.91255	0.0001006	0.0048185	0.9482485
C1s1	-3.089164	0.0298517	-13.52195	3.316E-08	3.885E-05	6.0248893
C3	-3.029261	5.4819189	-13.00219	4.988E-08	4.039E-05	9.1054319
Il1b	-2.79061	0.9983161	-5.155572	0.0003138	0.0101803	0.5778207
Fpr2	-2.75397	2.2349886	-6.286382	5.904E-05	0.0033748	2.1893282
Il1f9	-2.689229	0.7652352	-5.565409	0.0001678	0.0067551	1.0909576
Gm5424	-2.635428	-0.037659	-6.944372	2.42E-05	0.0018173	2.3600438
Slpi	-2.58316	4.0942363	-13.97002	2.358E-08	3.885E-05	9.7088969
Pilrb1	-2.555318	-0.016043	-5.358851	0.0002293	0.0084498	0.6763872
Slc39a4	-2.544429	2.7249375	-13.13169	4.499E-08	4.039E-05	8.5921688
Mmp14	-2.458664	5.5494897	-9.486086	1.233E-06	0.0003124	5.8230835
Cxcl2	-2.412981	2.5741269	-6.37879	5.191E-05	0.0031517	2.3202983
Cdc42ep2	-2.339047	1.356742	-6.435805	4.798E-05	0.0029473	2.2896299
Vcan	-2.293936	1.0811844	-9.550118	1.153E-06	0.0003124	5.1821694
H2-M2	-2.263884	-0.025817	-5.908183	0.0001012	0.0048185	1.3313855
Flrt3	-2.258072	0.2884686	-5.298435	0.0002516	0.008923	0.6809306
Ass1	-2.246744	5.4651865	-21.70557	2.158E-10	2.529E-06	14.310755
Aoah	-2.224852	3.2286014	-8.756397	2.707E-06	0.0004557	5.1678354
Gpr68	-2.143927	0.6183053	-7.123941	1.915E-05	0.00157	2.8551671
Slc6a12	-2.130059	1.6956073	-8.751873	2.721E-06	0.0004557	4.8398754
Clec4e	-2.094215	6.1809824	-10.23279	5.788E-07	0.0002055	6.5398319

Serinc2	-2.06939	-0.242901	-7.275118	1.577E-05	0.0013793	2.6081805
Adh7	-2.063364	3.3270907	-7.477435	1.222E-05	0.0011276	3.7035215
Calr3	-2.039214	0.4115836	-8.328287	4.398E-06	0.0006348	3.8571276
Gm34643	-2.021885	0.5747581	-8.247165	4.831E-06	0.0006555	3.8822796
Dcstamp	-1.97079	-0.182671	-4.229889	0.0014074	0.0306514	-0.813989
Traf1	-1.905311	1.8786635	-6.794078	2.952E-05	0.0020962	2.8168248
Pilrb2	-1.851497	0.5737161	-7.253323	1.622E-05	0.0013871	2.9837873
Arg2	-1.774621	0.4567476	-6.03898	8.381E-05	0.0043286	1.6398581
Uchl1	-1.762186	2.1406473	-7.694628	9.343E-06	0.0009522	3.9048622
Fgr	-1.710327	3.3789643	-8.152271	5.398E-06	0.0006858	4.5023855
Arl5c	-1.679333	2.3119331	-4.995458	0.0004033	0.0123306	0.3408637
Mefv	-1.665135	4.394827	-9.447149	1.284E-06	0.0003124	5.8725292
Ptges	-1.650936	3.1374654	-5.85853	0.0001088	0.0050578	1.5366405
Sdc1	-1.619766	5.0914557	-5.639284	0.0001502	0.0063096	0.848134
Tarm1	-1.607425	2.4252251	-5.928649	9.824E-05	0.0047765	1.7041925
Ednrb	-1.538019	2.9148764	-10.1932	6.018E-07	0.0002074	6.5571471
Gbp5	-1.536776	0.6943431	-4.626147	0.0007297	0.0192139	-0.174964
Gbgt1	-1.531404	2.7763857	-6.930396	2.464E-05	0.0018392	3.0332111
Htra1	-1.528438	-0.191177	-5.378456	0.0002226	0.0082526	0.6965997
Cav1	-1.515047	7.236193	-8.31954	4.442E-06	0.0006348	4.3259212
Pilra	-1.493566	3.4600141	-5.161796	0.0003108	0.0101803	0.4184096
Gm10134	-1.476416	3.3977698	-9.534135	1.173E-06	0.0003124	5.9904878
Ceacam19	-1.460358	2.1805128	-7.837427	7.854E-06	0.0008681	4.077329
Ctsk	-1.411589	5.3920516	-9.30016	1.499E-06	0.0003355	5.6034989
F10	-1.411438	3.4554333	-8.329376	4.392E-06	0.0006348	4.7003255
Cmklr1	-1.404569	3.9662226	-6.588447	3.893E-05	0.0025491	2.4412042
Src	-1.395582	3.8547826	-9.94555	7.7E-07	0.0002539	6.4084511
Il2rg	-1.391567	6.147073	-14.14749	2.066E-08	3.885E-05	9.9582528
Spic	-1.384822	1.1728384	-7.727124	8.98E-06	0.0009397	3.7060496
Gpr84	-1.364474	5.481174	-8.787581	2.615E-06	0.0004557	5.0145178
Socs3	-1.35703	2.395958	-6.728386	3.223E-05	0.0022086	2.7767068
2310001H17Rik	-1.337837	0.53141	-6.434807	4.804E-05	0.0029473	2.1294326
Ralgds	-1.29765	5.1766231	-13.37619	3.713E-08	3.955E-05	9.3954694
Acp5	-1.283867	3.3995879	-7.927197	7.05E-06	0.000802	4.2334334
Clec2d	-1.27754	1.8279064	-7.630697	1.011E-05	0.0009868	3.7903433
Bcam	-1.266455	-0.057388	-5.906342	0.0001015	0.0048185	1.3599395
Pde4b	-1.26345	3.0544808	-8.475886	3.712E-06	0.0005723	4.8673238
Tlr2	-1.24549	6.342033	-11.21388	2.29E-07	9.254E-05	7.473217
Slc6a13	-1.237947	0.3023085	-4.34092	0.0011684	0.0269486	-0.609727
Gfra2	-1.235798	3.386428	-7.080506	2.026E-05	0.0016206	3.1806184
2500002B13Rik	-1.232893	2.2823692	-4.998229	0.0004016	0.0123175	0.340843

Abca1	-1.202214	4.3986229	-5.781132	0.0001218	0.0054899	1.1782802
Tnip3	-1.200892	6.0302821	-7.566644	1.094E-05	0.001042	3.4516564
Nfkbie	-1.175815	4.8251775	-11.33166	2.059E-07	8.615E-05	7.6860137
Slamf8	-1.162248	1.0043064	-4.936514	0.0004428	0.0132355	0.2901726
Ifitm6	-1.148154	0.0952361	-3.89478	0.0024911	0.0447664	-1.278183
Ly75	-1.147546	1.8523405	-5.422626	0.0002081	0.0078284	1.0018606
Clec4a1	-1.14492	4.002493	-7.548268	1.119E-05	0.001049	3.7019266
Pcx	-1.143476	3.8394135	-11.85442	1.297E-07	7.101E-05	8.1424562
Trim29	-1.116144	1.0434196	-4.53995	0.0008403	0.0209932	-0.293183
Sod2	-1.110155	6.9389742	-8.757786	2.703E-06	0.0004557	4.8552262
Myrf	-1.088121	0.4948945	-4.033429	0.0019637	0.0380318	-1.062375
Atrnl1	-1.08447	1.5129851	-4.728237	0.0006182	0.0170818	-0.017617
S100a8	-1.076407	4.0506192	-6.728261	3.223E-05	0.0022086	2.6088529
Ceacam10	-1.010841	1.0480349	-7.145295	1.863E-05	0.001548	3.0726463
Relb	-1.004061	4.4629403	-8.804734	2.565E-06	0.0004557	5.1465888
Nqo1	-0.987986	5.0238747	-7.415583	1.321E-05	0.0011996	3.3766448
Treml4	-0.98492	4.6420963	-6.528486	4.224E-05	0.0026797	2.2260976
Sirpb1b	-0.97562	1.6664259	-3.958175	0.0022337	0.0419436	-1.251584
Ier3	-0.970342	4.0857948	-7.70588	9.216E-06	0.0009522	3.8843379
Gas7	-0.96939	6.4935022	-6.038563	8.386E-05	0.0043286	1.2603294
Hspa1b	-0.965634	1.1966857	-4.094998	0.0017682	0.0357821	-0.987422
Mcemp1	-0.958553	2.1491453	-4.613836	0.0007445	0.0194725	-0.249244
Acy1	-0.950605	0.9539775	-4.19439	0.0014942	0.0318316	-0.820527
Cxcl16	-0.948448	6.600032	-11.95749	1.186E-07	6.95E-05	8.1395281
Pstpip2	-0.94703	4.8451132	-9.719688	9.683E-07	0.0002986	6.1041669
Slc7a11	-0.942702	6.6344973	-3.795605	0.0029569	0.049074	-2.465965
Nfkbiz	-0.928516	3.0018653	-6.473286	4.556E-05	0.0028248	2.4069738
Gsap	-0.919127	5.6867521	-9.467886	1.257E-06	0.0003124	5.7481039
Zmynd15	-0.918248	1.6726061	-5.2097	0.0002885	0.0097195	0.6958483
Myo1b	-0.915954	1.8356465	-4.490933	0.0009109	0.0221896	-0.410132
Pdpn	-0.914157	6.3384537	-4.88081	0.0004839	0.0142446	-0.564699
Gbp2b	-0.906725	2.1586449	-5.550905	0.0001715	0.0068565	1.1747439
Rnd3	-0.896539	3.1264425	-7.583683	1.071E-05	0.0010286	3.8316755
Cybb	-0.89284	9.5889901	-13.59516	3.134E-08	3.885E-05	9.4972754
Tjp2	-0.889837	3.0080853	-5.910135	0.0001009	0.0048185	1.6120724
Isg20	-0.876532	0.4907338	-4.240012	0.0013836	0.0302461	-0.749943
Cpne8	-0.864944	1.7355987	-4.641823	0.0007113	0.018899	-0.164586
Rassf4	-0.862245	7.9890366	-9.41149	1.333E-06	0.0003124	5.5756009
Cd302	-0.859809	5.7597384	-12.7917	5.91E-08	4.328E-05	8.8994971
Spaca6	-0.858895	2.3930442	-6.737816	3.182E-05	0.0022063	2.7893611
Rnd1	-0.841679	0.2154133	-3.962483	0.0022173	0.0417015	-1.173043

Gpr132	-0.83962	2.704356	-5.344916	0.0002343	0.0085778	0.8139126
Tubb4a	-0.830466	1.1770555	-3.796206	0.0029538	0.049074	-1.466056
Gbp7	-0.829329	1.7991654	-4.786761	0.0005625	0.0160374	0.0548923
Met	-0.818705	5.5363845	-4.964251	0.0004237	0.0128437	-0.327535
Ikbke	-0.818313	6.4504305	-6.960821	2.368E-05	0.0017899	2.5930365
Fas	-0.814929	2.0143774	-5.85536	0.0001093	0.0050581	1.6112033
Aif1	-0.808038	3.7109491	-3.851479	0.0026843	0.0466109	-1.857331
Ust	-0.804049	3.4870264	-5.228808	0.0002801	0.0095217	0.4942467
Dner	-0.803788	3.1952552	-4.589099	0.0007753	0.0198015	-0.47749
Trem3	-0.793101	1.4804597	-3.909072	0.0024305	0.044289	-1.312066
Tnf	-0.786191	5.6067149	-6.92351	2.487E-05	0.0018442	2.6306322
Zc3h12c	-0.778482	5.1644812	-9.201493	1.666E-06	0.0003614	5.5085494
Ugt1a6b	-0.778327	3.3917762	-3.837122	0.0027518	0.0473859	-1.80331
Arhgef37	-0.777516	2.4756912	-5.228148	0.0002804	0.0095217	0.665651
Nfkbia	-0.772696	6.8618255	-7.065019	2.067E-05	0.0016362	2.7096024
Sirpb1a	-0.768971	3.9924087	-6.353575	5.376E-05	0.003214	2.0849581
Icam1	-0.767858	6.0678429	-9.166793	1.729E-06	0.0003683	5.376447
Ppfibp2	-0.766192	5.0767461	-9.596929	1.099E-06	0.0003124	5.9483915
C1rl	-0.765675	2.9578618	-7.993046	6.516E-06	0.000756	4.322187
Rrad	-0.763645	1.8702292	-5.311918	0.0002464	0.0088839	0.8395844
Birc3	-0.75864	4.8827066	-12.28809	8.956E-08	5.725E-05	8.5192492
Pla2g7	-0.757387	8.9006778	-9.496601	1.219E-06	0.0003124	5.6708595
Igsf6	-0.757102	6.8001229	-8.751317	2.722E-06	0.0004557	4.8497607
Cd74	-0.755398	2.982634	-5.918414	9.971E-05	0.0048185	1.6259518
Slc11a2	-0.754949	5.8161739	-11.35473	2.016E-07	8.615E-05	7.6339687
Zfp462	-0.753509	3.4311413	-5.154049	0.0003145	0.0101803	0.3865516
Gm7204	-0.747318	1.2568844	-4.015223	0.0020258	0.0389119	-1.119859
Gbp3	-0.746601	2.2254739	-4.51509	0.0008754	0.0216386	-0.420029
Marcksl1	-0.732403	7.1268917	-4.4992	0.0008986	0.0219805	-1.264133
Unc5b	-0.728339	2.3562423	-4.300809	0.0012494	0.0281307	-0.789778
Ms4a7	-0.722244	7.3438229	-13.23406	4.15E-08	4.039E-05	9.2064215
Gm43197	-0.718086	2.5223785	-4.73067	0.0006158	0.0170578	-0.11859
Tex15	-0.713337	1.8036812	-4.033543	0.0019634	0.0380318	-1.14707
Cd207	-0.700897	2.9018225	-4.033933	0.0019621	0.0380318	-1.346869
C1ra	-0.689904	4.2026105	-4.65997	0.0006906	0.0185594	-0.589467
Gm5150	-0.68703	3.1685225	-5.313499	0.0002458	0.0088839	0.6870151
Rbpms	-0.670336	2.9121133	-4.733977	0.0006125	0.017007	-0.183378
Zc3h12a	-0.669646	4.4683341	-5.118724	0.0003323	0.0105815	0.1096274
Mcoln2	-0.668532	3.9395852	-6.348519	5.414E-05	0.0032202	2.0853409
Nadk	-0.664311	7.2564899	-9.026955	2.011E-06	0.0004207	5.1519554
Maoa	-0.663844	6.7217154	-4.650693	0.0007011	0.0187986	-0.985737

Sirpb1c	-0.655795	3.3114899	-3.876263	0.0025719	0.0457278	-1.718215
Gm15922	-0.652139	4.9811847	-5.438026	0.0002033	0.0077302	0.5262174
Itga1	-0.64603	3.2501223	-7.181947	1.777E-05	0.0014873	3.3177002
Ppp1r12b	-0.642047	6.73487	-7.638232	1.001E-05	0.0009859	3.4795269
Ahrr	-0.640601	3.7578247	-6.315854	5.666E-05	0.0032867	2.0706881
Gbp2	-0.639625	2.7257844	-4.052954	0.0018994	0.0372036	-1.276877
Trim13	-0.637015	3.4079096	-6.11281	7.543E-05	0.0040916	1.8399187
Csf2rb	-0.636039	8.0631091	-7.648568	9.887E-06	0.0009859	3.4610425
Airn	-0.634892	1.8455888	-5.013737	0.0003919	0.0120512	0.3971888
Slc2a6	-0.633347	4.3859387	-7.123458	1.916E-05	0.00157	3.080591
Lpcat2	-0.631608	4.9394526	-5.646875	0.0001486	0.0062838	0.859909
Nfkb2	-0.630828	5.7449995	-9.424297	1.315E-06	0.0003124	5.6909365
Serpinb8	-0.628126	5.7839669	-11.77152	1.394E-07	7.101E-05	8.0160104
Tm4sf19	-0.619331	2.4901689	-3.938047	0.0023123	0.0429371	-1.42326
F7	-0.618697	3.5830213	-4.572906	0.0007961	0.0200164	-0.596589
Gbe1	-0.618327	5.0681097	-3.782155	0.0030267	0.0498792	-2.283349
Tnfaip2	-0.617743	7.8859911	-11.5249	1.732E-07	7.804E-05	7.7166276
Sema4b	-0.616497	4.5011707	-10.27938	5.53E-07	0.0002025	6.7022323
Lilra6	-0.615269	4.2968004	-8.503084	3.599E-06	0.0005623	4.8136627
Prdx1	-0.609473	10.802726	-4.529746	0.0008545	0.0212576	-1.194614
Dennd2a	-0.608241	3.5162098	-4.30693	0.0012367	0.0280273	-1.028908
Gm20559	-0.606947	4.3520955	-6.331989	5.54E-05	0.0032621	1.9868906
Lipn	-0.604701	3.4544331	-3.785312	0.0030102	0.0497654	-1.913926
2210406H18Rik	-0.602154	1.5106106	-3.831739	0.0027775	0.0476491	-1.443304
Scn1b	-0.60172	2.5358834	-4.50059	0.0008965	0.0219761	-0.494125
Mt2	-0.600534	2.3732736	-5.268017	0.0002636	0.00924	0.7365865
Ms4a14	-0.598599	3.4490192	-5.586091	0.0001627	0.0066404	1.0512235
Cd82	-0.597151	5.6354895	-8.921047	2.257E-06	0.0004327	5.1382159
Slfn2	-0.59483	7.1946502	-8.558566	3.38E-06	0.0005491	4.605908
Bcl3	-0.58385	4.1421085	-6.148459	7.17E-05	0.0039817	1.7577076
Grk3	-0.583573	4.1628425	-5.017129	0.0003898	0.0120185	0.0058012
Ptpn22	-0.582264	3.8657209	-5.038377	0.000377	0.0116845	0.1039037
Casp4	-0.58055	3.940911	-8.038287	6.175E-06	0.0007442	4.3053085
Slc16a3	-0.573345	6.8999321	-4.79699	0.0005534	0.0158895	-0.750068
Slc4a7	-0.572632	6.3396153	-8.783391	2.627E-06	0.0004557	4.9138663
Agpat4	-0.568855	5.144112	-5.161148	0.0003111	0.0101803	0.0544423
Glul	-0.568519	8.6536843	-5.817935	0.0001154	0.0052623	0.8683526
Mturn	-0.562969	2.9367452	-4.408365	0.0010443	0.024719	-0.725227
Cd52	-0.561398	5.6350392	-6.806491	2.903E-05	0.0020744	2.4611886
Bst1	-0.558112	6.4536649	-7.484772	1.211E-05	0.0011262	3.2966856
Rgl1	-0.553435	5.0931847	-5.561132	0.0001688	0.0067752	0.6997225

Slc31a2	-0.551234	5.6406051	-7.106034	1.96E-05	0.0015836	2.8728612
Trex1	-0.550421	4.6250445	-4.72351	0.000623	0.0171349	-0.573038
Gsta3	-0.546891	6.5051076	-4.12566	0.0016785	0.0346244	-1.876954
Pid1	-0.543175	8.2604128	-6.518396	4.283E-05	0.0026917	1.9120913
Ccrl2	-0.541357	2.8485063	-5.182651	0.0003008	0.0099865	0.5391631
Pde2a	-0.539993	6.0405128	-6.97942	2.311E-05	0.0017582	2.6527317
Btg3	-0.539694	2.280449	-3.963957	0.0022117	0.041663	-1.339551
Mocs1	-0.539247	5.0217011	-7.912155	7.178E-06	0.0008087	4.0062761
Mink1	-0.536721	7.3662317	-12.39339	8.2E-08	5.652E-05	8.499796
Klf7	-0.536223	4.5762313	-3.994429	0.0020992	0.0401237	-1.814162
Tmco4	-0.531295	3.6429054	-7.025783	2.175E-05	0.0016766	3.0644525
Sgms2	-0.531042	4.6059144	-8.924148	2.249E-06	0.0004327	5.2592162
Il1rn	-0.529921	5.3515314	-4.058262	0.0018823	0.0369731	-1.846981
Lmo4	-0.526619	5.4868766	-7.694409	9.346E-06	0.0009522	3.6689109
Acsl1	-0.525504	6.4243411	-6.559712	4.048E-05	0.0026352	2.0271056
Tnip1	-0.523695	6.7085132	-7.112917	1.942E-05	0.0015805	2.7815116
Col18a1	-0.522783	8.436904	-3.824381	0.0028131	0.0479089	-2.46411
Klra2	-0.52164	3.6672737	-4.672962	0.0006762	0.0183398	-0.451204
Csf2rb2	-0.521307	7.1026558	-5.062457	0.000363	0.01142	-0.318493
Mfsd7a	-0.518553	2.5003615	-3.835787	0.0027581	0.0473859	-1.600276
Hck	-0.517147	6.4741864	-4.662291	0.000688	0.018532	-0.948807
Myo10	-0.514917	6.2813985	-6.90022	2.564E-05	0.0018897	2.5198518
Ebi3	-0.511089	5.0018409	-7.812175	8.097E-06	0.0008867	3.8832817
Irak3	-0.509876	5.1642249	-7.077661	2.033E-05	0.0016206	2.9000179
Col4a2	-0.507135	2.5230685	-3.851293	0.0026852	0.0466109	-1.57888
Spint2	-0.505569	2.4457696	-4.017162	0.0020191	0.0388515	-1.281686
Ampd3	-0.500587	6.2237279	-8.008625	6.397E-06	0.0007507	3.9866438
Gpr141	-0.49984	4.8144582	-5.865162	0.0001077	0.0050291	1.2141451
Rnf149	-0.496727	8.1277636	-6.299552	5.797E-05	0.0033333	1.5931904
Ehd1	-0.494362	7.4528554	-5.664481	0.0001447	0.0061661	0.6367549
Gm10499	-0.491515	2.0570957	-4.122405	0.0016878	0.034673	-1.038043
Dst	-0.481033	6.8147045	-5.600954	0.0001591	0.0065401	0.5600344
Lyz1	-0.478512	3.8570148	-4.875208	0.0004882	0.0143365	-0.161446
Cfp	-0.477855	7.5813452	-4.45834	0.0009613	0.0231758	-1.347361
Rras	-0.476892	4.8818933	-8.04531	6.124E-06	0.0007442	4.1894935
Gm15931	-0.476194	4.7027137	-5.299812	0.000251	0.008923	0.3536432
H2-K2	-0.4653	4.3026371	-5.953502	9.478E-05	0.004674	1.4381233
Sdc4	-0.460304	6.4851221	-5.470509	0.0001935	0.007513	0.3765466
Atp6v0c-ps2	-0.457752	6.897881	-11.79685	1.363E-07	7.101E-05	7.9811042
Zmiz2	-0.456312	7.3599353	-8.99695	2.077E-06	0.000427	5.1144697
Ube2e2	-0.455066	3.0984551	-4.052173	0.0019019	0.0372036	-1.36526

Clec4a2	-0.453376	5.9754586	-7.584612	1.07E-05	0.0010286	3.4689224
Naaa	-0.452536	4.6318062	-5.575527	0.0001652	0.0066995	0.8007043
Prdx5	-0.452265	7.4812122	-8.907952	2.29E-06	0.0004327	5.0100496
Fos	-0.451978	6.8830246	-4.737422	0.0006091	0.0169932	-0.849996
Epn2	-0.445793	3.4363875	-4.777776	0.0005707	0.0160755	-0.226462
Slc16a10	-0.445367	7.7962832	-6.704249	3.329E-05	0.0022677	2.1807344
H2-K1	-0.438635	9.2115947	-5.205518	0.0002904	0.0097203	-0.094822
Rhoq	-0.436355	5.7051931	-5.254271	0.0002693	0.0093622	0.1156094
N4bp1	-0.436261	6.2456026	-7.263944	1.6E-05	0.0013871	3.0191378
Gmfg	-0.43443	5.4957105	-5.299032	0.0002513	0.008923	0.2187744
Nr1h3	-0.430217	3.9367938	-4.440975	0.0009893	0.0237538	-0.90394
Lrmda	-0.425019	3.1693856	-5.457412	0.0001974	0.0075781	0.9056512
S100a10	-0.423175	7.7306701	-5.468723	0.0001941	0.007513	0.3242315
Stac2	-0.418633	5.2601761	-3.943189	0.002292	0.0427519	-2.034327
Adgre1	-0.416805	9.2234508	-6.81089	2.886E-05	0.0020744	2.3356786
Lst1	-0.412268	3.9962671	-5.207417	0.0002895	0.0097195	0.3448893
Mcl1	-0.412098	9.2060211	-8.918114	2.264E-06	0.0004327	5.0219445
Traf3	-0.410371	5.8667518	-6.685527	3.414E-05	0.0023121	2.2604928
Gtf3c5	-0.410326	4.6277341	-6.335207	5.515E-05	0.0032621	1.941832
Tnfrsf1b	-0.40978	8.6929007	-7.0516	2.103E-05	0.0016538	2.6641327
Mtmr14	-0.409364	5.0898651	-4.633251	0.0007213	0.0190645	-0.81142
Atp6v0c	-0.406977	9.5265514	-9.510825	1.202E-06	0.0003124	5.6928671
Gm5537	-0.40674	3.4642224	-4.321043	0.0012078	0.0275868	-0.995431
Tmem119	-0.405594	3.379266	-4.25716	0.0013443	0.0295521	-1.083238
Adam17	-0.404993	8.3335829	-7.671325	9.614E-06	0.0009711	3.4894626
Slc25a37	-0.400653	6.3267541	-6.009681	8.741E-05	0.0043956	1.224502
Abcc1	-0.399288	8.3655578	-6.096672	7.718E-05	0.0041674	1.2908163
Eepd1	-0.393412	4.6780675	-4.916379	0.0004572	0.0135515	-0.264632
Malt1	-0.38973	5.596381	-5.072496	0.0003573	0.0113072	-0.16402
Mlkl	-0.387781	4.3334984	-5.643434	0.0001493	0.0062933	0.9607381
Orai2	-0.383426	5.3823114	-5.24142	0.0002747	0.0094655	0.1438103
Ncf4	-0.382102	7.6472849	-8.663124	3.004E-06	0.0004957	4.7215224
Psmd10	-0.381475	4.407503	-4.470022	0.0009429	0.0228738	-0.958846
Radx	-0.380045	4.3696525	-6.01291	8.7E-05	0.0043941	1.5135597
Acaa1a	-0.378404	6.6776185	-5.478872	0.0001911	0.0074887	0.3754877
Itgb2	-0.377633	9.9103892	-5.873003	0.0001065	0.0049919	0.9694173
Atox1	-0.373238	5.939129	-5.723788	0.0001326	0.005817	0.827021
Spata13	-0.372399	4.7838529	-5.759273	0.0001258	0.0056045	1.0568968
Prdm1	-0.371646	3.6972837	-3.895697	0.0024871	0.0447643	-1.786302
Ube2f	-0.370617	5.4014827	-7.742668	8.811E-06	0.0009397	3.7409866
Pirb	-0.369826	7.3072266	-4.582664	0.0007835	0.019956	-1.129384

Parp8	-0.366685	3.4063918	-4.288311	0.0012758	0.0285833	-1.037226
Tor3a	-0.366378	7.1497777	-6.065388	8.07E-05	0.0042524	1.2585014
Junb	-0.364707	6.9256836	-4.125762	0.0016782	0.0346244	-1.905862
Emb	-0.364024	8.3782077	-9.28879	1.518E-06	0.0003355	5.4372401
Gdpd1	-0.361867	5.031098	-6.003599	8.818E-05	0.0044152	1.3864384
Abcd4	-0.361609	3.7129257	-3.947112	0.0022766	0.0425433	-1.700649
Txn1	-0.359559	8.0303053	-4.091348	0.0017792	0.0358809	-1.990864
Mvp	-0.358164	7.320916	-8.288148	4.607E-06	0.0006426	4.2751897
Abcc5	-0.357408	7.3783633	-7.878592	7.474E-06	0.000834	3.763514
Acp2	-0.357345	7.2295724	-5.853077	0.0001096	0.0050581	0.9333507
P2rx4	-0.356553	7.0413513	-5.43618	0.0002039	0.0077302	0.2878721
Cers6	-0.355844	6.85576	-7.770087	8.522E-06	0.0009245	3.6419199
Baiap2	-0.354781	5.2828273	-3.821786	0.0028258	0.047997	-2.253431
Blvrb	-0.353496	7.5174597	-5.171931	0.0003059	0.0100962	-0.150549
Pde4dip	-0.350648	5.5795077	-5.426034	0.000207	0.0078248	0.4082197
Tgfbi	-0.349587	7.6094774	-6.055967	8.18E-05	0.0042596	1.2346443
Pgk1	-0.349527	9.1364275	-4.632314	0.0007224	0.0190645	-1.049035
Lyz2	-0.347455	14.399789	-8.387789	4.106E-06	0.0006107	4.4860856
Plxnd1	-0.346443	8.3011433	-5.421492	0.0002085	0.0078284	0.2452504
Tpm1	-0.345838	6.5930483	-4.959078	0.0004272	0.0128686	-0.461652
Neurl3	-0.345173	5.9356512	-5.892087	0.0001036	0.0048945	1.0859324
Plxna1	-0.344673	9.2690943	-7.034019	2.152E-05	0.0016697	2.6463377
Cav2	-0.343677	6.9463662	-4.066601	0.0018557	0.0366055	-2.010983
Rnf19b	-0.342711	6.8568331	-4.059454	0.0018785	0.0369731	-2.018744
Phlpp1	-0.341691	4.1514956	-4.14837	0.0016151	0.033914	-1.455771
Sema4a	-0.336386	5.2341442	-6.298729	5.803E-05	0.0033333	1.7918262
St18	-0.334701	4.0672251	-3.843307	0.0027225	0.0470496	-1.969024
Ninj1	-0.334598	7.5761194	-4.076594	0.0018244	0.03646	-2.011961
St3gal1	-0.334065	6.6281112	-6.320306	5.631E-05	0.0032826	1.6637385
Mllt6	-0.332257	5.5906318	-5.30807	0.0002479	0.0089091	0.2181793
Mapkapk2	-0.332152	7.6354797	-5.607773	0.0001575	0.0065196	0.544892
Ncoa4	-0.331939	6.9269527	-7.72994	8.949E-06	0.0009397	3.586958
B2m	-0.330719	9.7206765	-6.118386	7.483E-05	0.0040781	1.3380933
Vasp	-0.329727	7.1410715	-5.156557	0.0003133	0.0101803	-0.166748
Ptprj	-0.329119	8.2429111	-5.713075	0.0001347	0.0058658	0.7046249
Tapbp	-0.328636	8.1288011	-6.36214	5.313E-05	0.0031923	1.684978
Myadm	-0.328303	8.2628088	-8.007322	6.407E-06	0.0007507	3.9181159
Prkcb	-0.327827	5.9246814	-4.608758	0.0007507	0.019504	-0.983631
H2-Q4	-0.327607	7.3695807	-5.278798	0.0002593	0.0091507	0.0254885
Alas1	-0.327576	6.45173	-6.757404	3.1E-05	0.002162	2.3051843
Pik3r5	-0.326957	7.9576912	-6.611835	3.771E-05	0.0025107	2.0475658

Ahnak2	-0.325161	6.1663644	-5.747701	0.000128	0.0056792	0.8388188
Cd47	-0.322822	7.3317744	-8.055832	6.048E-06	0.0007442	3.9876676
Ephx1	-0.322464	3.9691	-3.916145	0.0024011	0.0438897	-1.81787
Akr1b8	-0.320821	6.5592024	-3.947455	0.0022752	0.0425433	-2.196578
Rilpl2	-0.320554	5.5526195	-6.12616	7.401E-05	0.0040526	1.4892439
Smurf1	-0.318805	5.7858309	-4.994247	0.0004041	0.0123306	-0.320395
Ugdh	-0.316839	6.8067856	-4.574597	0.0007939	0.0200164	-1.122297
Zfp608	-0.315293	3.9024893	-3.867564	0.0026108	0.0462241	-1.886869
Slc11a1	-0.314155	9.0749092	-4.605006	0.0007553	0.0195806	-1.096337
Samhd1	-0.309626	7.4531867	-5.047935	0.0003713	0.01156	-0.352627
Susd6	-0.308249	7.3365346	-6.402304	5.025E-05	0.0030666	1.7527465
Ctsz	-0.308159	10.083525	-6.243909	6.267E-05	0.0035289	1.5309139
Ifngr2	-0.307866	6.7042376	-8.547718	3.421E-06	0.0005491	4.6115175
Gm4735	-0.307183	7.5993059	-3.826449	0.0028031	0.0478072	-2.455185
Mcub	-0.305472	5.3670017	-4.284505	0.001284	0.0286567	-1.456306
Anxa1	-0.304306	9.3639801	-3.861971	0.0026361	0.0462241	-2.387358
Btg1	-0.304096	7.019119	-4.330183	0.0011895	0.0273285	-1.553917
Acsl4	-0.300415	6.9093242	-4.083427	0.0018033	0.036132	-1.979649
Igf2r	-0.300288	9.2326851	-8.136069	5.502E-06	0.0006858	4.085745
Rab11fip1	-0.298456	4.5143996	-5.266651	0.0002642	0.00924	0.3342762
Cstb	-0.297245	8.3137509	-4.929543	0.0004477	0.0133149	-0.556188
Ptpro	-0.293913	5.4916281	-3.809899	0.0028846	0.0484219	-2.310506
Lilrb4a	-0.293048	8.7506985	-5.297147	0.0002521	0.008923	0.0477959
Gm9844	-0.293028	3.7600062	-3.799214	0.0029385	0.0490066	-1.97171
Hvcn1	-0.292881	7.6702319	-5.118936	0.0003322	0.0105815	-0.239283
Mtpn	-0.291724	9.0791945	-4.793981	0.0005561	0.0158909	-0.77669
Tpm4	-0.290609	9.1520048	-4.367183	0.0011183	0.0261021	-1.503585
Sqor	-0.288963	5.2091497	-4.807265	0.0005443	0.0157863	-0.540191
Eno1	-0.288302	10.129845	-4.300126	0.0012508	0.0281307	-1.603966
Sh3bgrl2	-0.28767	5.9282988	-5.127726	0.0003277	0.0105196	-0.119866
Plscr1	-0.287449	3.6969104	-3.920957	0.0023813	0.043698	-1.742654
Myo1g	-0.287165	7.0356866	-5.709919	0.0001353	0.0058714	0.7187168
Zc3h12d	-0.282935	5.3152281	-4.418225	0.0010273	0.0244363	-1.216878
Pnrc1	-0.282323	4.8593712	-3.899816	0.0024695	0.0445161	-2.038974
Rbpj	-0.280455	7.9619234	-5.23459	0.0002776	0.0095217	-0.053829
Aldoa	-0.278061	10.892201	-4.633571	0.0007209	0.0190645	-1.015834
Tfec	-0.27797	4.3571642	-4.53387	0.0008487	0.0211589	-0.840584
Arap1	-0.277655	8.1190199	-6.24074	6.295E-05	0.0035289	1.5061477
H2-D1	-0.277491	9.9839345	-3.942252	0.0022957	0.0427519	-2.234675
Gm11427	-0.270322	4.3617995	-4.315664	0.0012187	0.027782	-1.21377
Vmp1	-0.268727	6.1076516	-6.645551	3.603E-05	0.0024124	2.17707

Sgk1	-0.268473	6.0575663	-5.505055	0.0001837	0.0072715	0.4709997
Lactb	-0.268191	5.1166963	-4.194413	0.0014941	0.0318316	-1.569502
Zfp263	-0.26714	5.9175383	-6.247151	6.238E-05	0.0035289	1.6204808
Lmna	-0.265004	9.5961843	-4.083216	0.001804	0.036132	-1.992455
Rbpj-ps3	-0.264916	6.2285296	-5.047961	0.0003713	0.01156	-0.283807
Pygl	-0.263423	6.7247573	-4.369425	0.0011141	0.0260567	-1.469853
Sema4d	-0.26281	8.6508936	-6.077928	7.927E-05	0.0042412	1.2637089
Mthfd1l	-0.262462	5.1264306	-3.996665	0.0020911	0.0400357	-1.917174
Aldh3b1	-0.262421	6.6933758	-5.182602	0.0003009	0.0099865	-0.101859
Gys1	-0.261788	5.030826	-4.211993	0.0014505	0.0312762	-1.523827
Itgam	-0.259831	9.8612669	-5.949409	9.534E-05	0.004674	1.0852245
Ugt1a7c	-0.253928	7.4710634	-5.158245	0.0003125	0.0101803	-0.172221
Ggh	-0.252664	5.2196605	-4.573962	0.0007947	0.0200164	-0.934817
G6pdx	-0.251739	7.8494118	-6.321103	5.625E-05	0.0032826	1.6266322
Arl6ip5	-0.247457	7.2800816	-6.248078	6.23E-05	0.0035289	1.5272009
S100a11	-0.245313	7.4587925	-4.960118	0.0004265	0.0128686	-0.49785
Pfkl	-0.245152	6.7570954	-4.132917	0.0016579	0.0344434	-1.883824
Agtrap	-0.244011	6.0620018	-4.269145	0.0013175	0.0291719	-1.584132
Tank	-0.242772	5.3994644	-4.211327	0.0014521	0.0312762	-1.589056
Ccdc50	-0.24223	7.2093149	-4.234586	0.0013963	0.0304667	-1.727464
Slc44a1	-0.2421	7.0685011	-5.447852	0.0002003	0.007644	0.3050247
Abcd1	-0.241673	6.5160859	-6.027764	8.517E-05	0.0043388	1.2357446
Ecm1	-0.241066	7.5365962	-3.813989	0.0028642	0.0482167	-2.476454
Snx10	-0.238825	6.3500279	-4.679219	0.0006693	0.0181965	-0.910681
Pabpc1	-0.238776	10.049901	-6.085127	7.846E-05	0.0042171	1.2935391
Vim	-0.237104	11.756883	-4.266529	0.0013233	0.0292003	-1.62796
P4hb	-0.236508	9.413047	-4.915279	0.000458	0.0135515	-0.569191
Anxa4	-0.236449	8.5720206	-5.046868	0.0003719	0.01156	-0.361499
Pstpip1	-0.236114	6.7536081	-4.101741	0.001748	0.0355586	-1.938337
Sestd1	-0.235179	6.0515835	-5.26463	0.000265	0.0092413	0.0877168
Diaph2	-0.234528	6.0981285	-3.903594	0.0024535	0.0444865	-2.230185
Crybg1	-0.233542	7.0349993	-4.738646	0.0006079	0.0169932	-0.856076
Gm7336	-0.230619	6.850047	-5.13588	0.0003236	0.0104151	-0.187461
Notch2	-0.228443	7.6124035	-3.92653	0.0023585	0.0435199	-2.277643
Gpx1	-0.225174	9.5964821	-3.922604	0.0023745	0.0436771	-2.276102
Sh3tc1	-0.223329	5.8526494	-4.170844	0.0015548	0.0329436	-1.729451
Camkk2	-0.223174	6.4448412	-3.929472	0.0023466	0.0433685	-2.219128
Large1	-0.222097	5.3985802	-3.865706	0.0026192	0.0462241	-2.196016
Irf2bp2	-0.217978	7.6002465	-4.11719	0.0017028	0.0347607	-1.941027
C9orf72	-0.216672	5.6539986	-4.13933	0.00164	0.0342532	-1.756029
Syk	-0.216045	8.5722911	-4.257644	0.0013432	0.0295521	-1.700268

Pofut2	-0.215456	6.3059669	-3.863764	0.002628	0.0462241	-2.322969
Nampt	-0.214675	7.5301296	-4.857031	0.0005026	0.0146488	-0.670564
Tmsb10	-0.213619	9.0425091	-4.253224	0.0013532	0.0296925	-1.703
Swap70	-0.210693	6.4711703	-4.354743	0.0011417	0.0264907	-1.476497
Anxa5	-0.206139	9.1713797	-3.967101	0.0021998	0.0415057	-2.20377
Ahnak	-0.205431	11.276781	-4.932154	0.0004459	0.0132935	-0.506228
Cpne2	-0.204115	7.0712143	-4.417734	0.0010282	0.0244363	-1.404987
Bub1	-0.200974	6.2742289	-4.029012	0.0019786	0.0381942	-2.027468
Ptprs	-0.198898	6.6783827	-3.9796	0.0021532	0.0409555	-2.148604
Myl12b	-0.198661	7.1067009	-4.159327	0.0015854	0.0334104	-1.855601
Arhgef10l	-0.198349	5.9240423	-4.38256	0.00109	0.0256273	-1.371203
Cyp4v3	-0.197212	6.1128195	-3.8027	0.0029208	0.0488198	-2.411111
Cyba	-0.196624	8.6552672	-4.088447	0.001788	0.0359347	-1.995873
Csf2ra	-0.196042	8.047557	-4.588745	0.0007757	0.0198015	-1.129088
Nab1	-0.195739	6.7641682	-3.932264	0.0023354	0.0432974	-2.238015
Gm6166	-0.195389	7.650363	-4.14167	0.0016335	0.0341786	-1.898753
Gpi1	-0.192314	8.8722486	-4.518306	0.0008707	0.0216156	-1.247024
Rab10	-0.191819	7.414108	-4.146078	0.0016214	0.033985	-1.887311
Nfe2l2	-0.188904	7.5086099	-4.286927	0.0012788	0.028595	-1.642993
Gsr	-0.188005	7.9583564	-3.861432	0.0026386	0.0462241	-2.397005
Gapdh	-0.187902	11.072303	-4.309399	0.0012316	0.028006	-1.568751
Surf4	-0.186523	8.1067636	-4.473192	0.000938	0.0228015	-1.327238
Dock7	-0.18493	6.4897006	-4.868977	0.0004931	0.0144076	-0.603845
Snd1	-0.18362	7.2032253	-5.161143	0.0003111	0.0101803	-0.161504
Slc31a1	-0.182367	7.0987272	-4.116179	0.0017057	0.0347607	-1.930953
Rac2	-0.181571	8.1099402	-5.243669	0.0002737	0.0094606	-0.040103
Cs	-0.180385	7.9572541	-4.782516	0.0005664	0.0160755	-0.800359
Fbxo33	-0.180205	5.6069275	-3.860151	0.0026444	0.0462241	-2.240477
Sppl2a	-0.180177	7.0449048	-4.941385	0.0004394	0.0131671	-0.517369
Capza2	-0.179487	8.1228402	-4.643081	0.0007099	0.018899	-1.037051
Ifnar1	-0.178504	7.5556333	-4.206502	0.001464	0.0314739	-1.783927
Tra2a	-0.175892	5.4669778	-3.920504	0.0023831	0.043698	-2.110691
Slc43a2	-0.175657	9.5396126	-4.227945	0.001412	0.0306948	-1.739776
Itpr2	-0.172534	6.3453351	-4.135293	0.0016513	0.0343659	-1.847583
Slc25a24	-0.171871	6.9487591	-4.803322	0.0005478	0.0158474	-0.743353
Ensa	-0.165426	6.7157311	-3.887963	0.0025205	0.045157	-2.313386
Hal	-0.164206	10.036224	-4.151523	0.0016065	0.0337939	-1.865
Sh3bgrl3	-0.160558	8.5500016	-4.072905	0.0018359	0.036522	-2.023947
Psmd8	-0.160233	7.5697381	-4.07432	0.0018315	0.0364961	-2.015968
Ptprc	-0.153669	8.3928187	-4.268535	0.0013189	0.0291719	-1.681946
Arpc1b	-0.15265	9.7277708	-4.165363	0.0015693	0.0331903	-1.846134

Tubgcp4	-0.149523	6.384595	-3.797116	0.0029492	0.049074	-2.448887
Stom	-0.149431	8.6324245	-4.100596	0.0017514	0.0355661	-1.974711
Mdc1	-0.148246	6.3782638	-3.984234	0.0021361	0.0406977	-2.11642
Serp1	-0.147735	7.2600997	-3.851851	0.0026826	0.0466109	-2.403404
Ncf2	-0.136784	7.8730076	-3.843848	0.00272	0.0470496	-2.427508
Itgb1	0.1287781	9.4848078	3.902101	0.0024598	0.0444865	-2.314187
Arhgef7	0.140889	7.7913173	3.7988543	0.0029403	0.0490066	-2.506579
Mafb	0.1498136	8.6162848	4.2212907	0.0014279	0.0308756	-1.763057
Pip4k2a	0.149974	6.7283911	4.1161254	0.0017058	0.0347607	-1.910505
Parvb	0.1501941	7.4691479	4.5168795	0.0008728	0.0216205	-1.244959
Wdr26	0.1517413	8.4144426	3.9029847	0.0024561	0.0444865	-2.324465
Ncor2	0.1520893	8.3075221	4.0674596	0.001853	0.0366055	-2.033744
Tesk1	0.1552496	7.2456828	4.0453253	0.0019243	0.0375155	-2.059597
Hipk3	0.1594101	7.4595265	3.865813	0.0026187	0.0462241	-2.382572
Tmem9b	0.1641408	5.8544124	3.9565763	0.0022399	0.0419915	-2.104242
Gng5	0.1654629	6.6032185	4.4150522	0.0010328	0.0244956	-1.381417
Bin2	0.1759858	6.7851196	3.9300422	0.0023443	0.0433685	-2.242099
Furin	0.177788	7.7442827	3.7913746	0.0029787	0.0493654	-2.519315
Rc3h2	0.1834019	5.8119731	3.8084311	0.0028919	0.0484757	-2.360712
Cap1	0.1848149	10.3979	5.0258354	0.0003845	0.011886	-0.369193
Sh2b3	0.185251	7.0574383	4.4021541	0.0010551	0.0248748	-1.429928
Milr1	0.1923975	6.391133	4.5122411	0.0008795	0.021649	-1.196881
Nt5c2	0.1942039	5.206923	3.836361	0.0027554	0.0473859	-2.212805
Tbxas1	0.1943142	6.9491731	4.0983177	0.0017582	0.0356423	-1.954211
Plxdc1	0.1945494	6.5974612	4.7782252	0.0005703	0.0160755	-0.762567
Abhd5	0.1960872	6.0633977	3.9126938	0.0024154	0.0440823	-2.208182
Cd300lb	0.1983599	8.0846566	4.6112673	0.0007477	0.019504	-1.090593
Nfya	0.2002731	7.5950342	5.7355144	0.0001303	0.0057387	0.7450741
Wipf1	0.2003808	7.8719122	4.1809513	0.0015285	0.0324443	-1.832136
Pithd1	0.2008192	4.5273923	3.9414859	0.0022987	0.0427519	-1.896419
Tlr13	0.2016117	8.9813671	4.0577814	0.0018838	0.0369731	-2.046126
Cux1	0.2034811	8.2182228	4.7955547	0.0005546	0.0158895	-0.779497
Paqr7	0.2086851	5.5588767	4.5741686	0.0007944	0.0200164	-0.988321
Edem3	0.2088259	6.5732469	4.8690786	0.000493	0.0144076	-0.608543
Commd9	0.2094042	4.8049594	3.8791428	0.0025591	0.0456863	-2.062301
1700017B05Rik	0.2094571	7.1359707	3.8216439	0.0028265	0.047997	-2.451796
Tnfsf12	0.2101105	6.3056595	3.8155302	0.0028566	0.0481588	-2.406718
Extl3	0.2101708	7.0925743	4.0290016	0.0019787	0.0381942	-2.082568
Cd300a	0.2122762	7.399439	4.1951446	0.0014923	0.0318316	-1.80031
Atxn10	0.2138489	6.693245	4.0759005	0.0018266	0.03646	-1.978292
Smg6	0.2176225	4.7785103	3.8917944	0.0025039	0.0449284	-2.034728

Tmem185a	0.2177347	4.6267969	4.051178	0.0019052	0.0372047	-1.724455
Nab2	0.2226914	5.0655817	4.3013112	0.0012484	0.0281307	-1.372991
Ldlrap1	0.2232339	7.3715061	4.9635562	0.0004242	0.0128437	-0.489647
H2-DMa	0.2254755	4.9613049	3.815729	0.0028556	0.0481588	-2.204259
Soga1	0.2282956	6.0217422	4.3701215	0.0011129	0.0260567	-1.402251
Cyb5r1	0.229046	5.9938907	3.9167486	0.0023986	0.0438897	-2.192092
Isyna1	0.2309989	6.0461912	4.2236521	0.0014223	0.0308603	-1.659347
Adipor1	0.2314094	7.7386686	5.3057602	0.0002488	0.0089134	0.0636163
Fhod1	0.2325521	5.9725718	5.1246201	0.0003293	0.0105418	-0.127927
Retreg1	0.2326122	5.6271534	3.901991	0.0024603	0.0444865	-2.16649
Zfp715	0.2326401	4.9142927	4.7034768	0.0006435	0.0175598	-0.660321
Ptpra	0.2327129	8.946025	4.2775227	0.0012991	0.0289391	-1.661725
Slc39a8	0.2331101	4.3502603	4.3818112	0.0010914	0.0256273	-1.094321
Tnfsfm13	0.2352054	5.1695233	4.3279166	0.001194	0.0273785	-1.345381
Dgkz	0.2365804	8.6273786	5.6052403	0.0001581	0.0065212	0.5374397
Rgs10	0.2391455	6.2815907	5.4598294	0.0001967	0.0075781	0.3783726
Kif13b	0.2418549	6.4309623	3.9039831	0.0024519	0.0444865	-2.261139
Gnptab	0.2427856	8.081289	4.4021798	0.0010551	0.0248748	-1.449011
Lrp12	0.2435245	6.7931677	6.6501696	3.581E-05	0.0024111	2.1320903
Usp2	0.2481084	4.8694043	3.9756184	0.0021679	0.0411691	-1.90423
Galns	0.2490029	7.2326634	5.0711639	0.000358	0.0113072	-0.308094
Ahcyl2	0.2492728	6.2788818	4.702609	0.0006444	0.0175598	-0.862151
Mgat5	0.2496126	7.3666552	6.5162452	4.296E-05	0.0026917	1.9182936
Itpr3	0.2498414	7.7649156	4.5761811	0.0007918	0.0200164	-1.14765
Gask1b	0.2498435	4.4730546	4.2211634	0.0014282	0.0308756	-1.396427
Rhob	0.2506938	7.0902891	4.1217281	0.0016897	0.034673	-1.918957
Lemd3	0.2521031	4.5336821	4.1633157	0.0015747	0.0332455	-1.509311
Gorasp1	0.252547	4.560028	4.3085407	0.0012333	0.028006	-1.26364
Fam107b	0.2540675	5.1983921	3.8131778	0.0028682	0.0482167	-2.251848
Arhgap25	0.2542753	6.4124683	4.7715194	0.0005765	0.0161993	-0.758395
Hacd2	0.2550211	5.0050522	4.5733887	0.0007954	0.0200164	-0.895923
Piezo1	0.2556909	8.9592031	7.0410005	2.132E-05	0.0016656	2.6522945
Tmx4	0.2631049	6.901592	5.5305647	0.0001768	0.0070452	0.4456988
Numb	0.2667486	6.7878568	6.2032312	6.636E-05	0.0037026	1.4823773
Lyrm9	0.2683321	3.6855418	3.8164335	0.0028521	0.0481588	-1.917736
Luzp1	0.2697909	6.6435671	4.2977702	0.0012558	0.0281878	-1.586591
Man2b1	0.270611	9.1043711	3.8409601	0.0027336	0.0471714	-2.428356
Ppt1	0.2706382	7.1247585	5.8469457	0.0001106	0.0050835	0.9287867
Nt5dc2	0.2734671	7.5398192	5.9771849	9.159E-05	0.0045668	1.1169451
Lrp1	0.2752807	11.841217	5.1470565	0.000318	0.0102634	-0.140051
Apbb1ip	0.2756387	7.8357466	6.5273528	4.231E-05	0.0026797	1.9275684

Tpd52	0.2757144	7.4052566	6.0725638	7.988E-05	0.0042524	1.2636517
Dgkd	0.2766118	7.2819342	7.5533198	1.112E-05	0.001049	3.3470482
Cnr2	0.2777359	5.1381517	3.8590025	0.0026497	0.0462241	-2.159657
R3hdm1	0.277946	7.4079344	5.9736134	9.207E-05	0.0045709	1.1140598
Cttnbp2nl	0.2780289	7.2772521	6.5397121	4.16E-05	0.0026773	1.9546423
Smim13	0.278561	4.9810196	3.8292728	0.0027894	0.0477829	-2.183323
Glb1	0.2814317	6.918708	4.4558829	0.0009652	0.0232224	-1.330025
Ctsh	0.2821804	6.2627977	4.0061089	0.0020576	0.0394584	-2.064115
Abhd12	0.2833727	8.0108822	6.1297613	7.363E-05	0.0040526	1.3419352
Irf2bp1	0.2854052	4.6872252	4.7151447	0.0006315	0.0173277	-0.59736
Oip5os1	0.2883388	6.3658283	4.4959023	0.0009035	0.0220543	-1.221513
Etv5	0.2935631	6.7971725	5.3126814	0.0002461	0.0088839	0.1041135
Fam98c	0.2964363	3.6048308	4.1990822	0.0014824	0.0317669	-1.233202
Traf6	0.296526	5.6567267	5.5799608	0.0001642	0.0066783	0.6429809
Vipas39	0.2981737	6.8162332	4.3262507	0.0011974	0.0274012	-1.548265
Ltbp3	0.2995848	5.6490574	4.1305902	0.0016645	0.0345184	-1.766568
Atp6v0a1	0.2996272	8.301459	4.726883	0.0006196	0.0170818	-0.895038
Hip1	0.302495	8.3367301	8.3892064	4.1E-06	0.0006107	4.389921
Hexb	0.305784	7.6439537	6.7686815	3.054E-05	0.0021424	2.273921
Cpeb2	0.3087572	5.8187209	6.1260889	7.402E-05	0.0040526	1.4565026
Mfsd6	0.3088853	6.8646275	3.8598088	0.002646	0.0462241	-2.370334
Nek6	0.3101963	6.389723	5.7168598	0.0001339	0.0058549	0.7728118
Pgm2l1	0.3109063	6.964039	5.1806317	0.0003018	0.0099888	-0.11819
Mef2c	0.3134383	5.7855526	5.7382816	0.0001297	0.0057369	0.8720244
Mpzl1	0.3154405	3.7759888	4.4384733	0.0009934	0.0238036	-0.865988
Cdkn1a	0.3184193	7.668594	7.2780136	1.572E-05	0.0013793	2.974887
Snta1	0.3184599	4.2657715	3.8785516	0.0025617	0.0456863	-1.94775
Lrp5	0.3264529	6.3821113	5.2491715	0.0002714	0.0094083	0.0327175
Bmp2k	0.3321801	7.0457889	5.0614113	0.0003635	0.01142	-0.316611
Rasal3	0.3330876	5.160486	4.3650446	0.0011223	0.0261432	-1.278729
Cdk19	0.3341218	6.312552	6.9878061	2.286E-05	0.0017504	2.6400899
AU020206	0.3341993	6.7575736	4.6094988	0.0007498	0.019504	-1.057596
Zmat3	0.335376	5.1135655	4.545872	0.0008322	0.0208345	-0.960899
Zfpm1	0.3381892	4.7722698	5.7837165	0.0001214	0.0054899	1.1000592
Scoc	0.3396278	5.0398948	4.6468585	0.0007055	0.0188731	-0.776587
Tgfbr1	0.3401859	7.2893335	5.8216617	0.0001148	0.0052542	0.8851613
Pros1	0.3420324	5.0517936	3.9687447	0.0021936	0.0414556	-1.949186
Nipal3	0.343388	5.0584731	3.993264	0.0021033	0.0401383	-1.907298
Rgs2	0.3449543	6.756963	4.3629811	0.0011262	0.0261812	-1.48043
Fzd5	0.3468399	4.11547	5.9501727	9.523E-05	0.004674	1.4722548
Mical1	0.3468738	5.8237711	4.5894157	0.0007748	0.0198015	-0.999114

Inppl1	0.3493134	8.4538294	7.9826486	6.598E-06	0.0007579	3.8878642
Ctsd	0.3517628	13.582615	5.3953804	0.0002169	0.0081121	0.3016912
Thbd	0.3534967	4.119959	4.1194028	0.0016964	0.0347492	-1.492462
Arhgap24	0.3545477	4.816228	3.7822252	0.0030264	0.0498792	-2.23366
Xylt1	0.3549682	6.9743237	4.2405102	0.0013825	0.0302461	-1.705146
Cd93	0.3554641	9.237067	4.3448489	0.0011607	0.0268782	-1.540876
Chst10	0.3600955	5.4256497	4.1354141	0.0016509	0.0343659	-1.721832
Dhrs3	0.3604024	7.1317642	4.9518287	0.0004322	0.012984	-0.500717
Plxnc1	0.3638558	6.7284778	3.8172929	0.0028479	0.0481588	-2.437013
Dcakd	0.3673674	4.7486719	4.60257	0.0007584	0.0196153	-0.797235
Pcgf2	0.373068	3.4711872	4.0170913	0.0020193	0.0388515	-1.512062
Plk3	0.3763055	4.8322038	5.2336225	0.000278	0.0095217	0.2278947
Arhgap22	0.3773868	5.6084989	4.615746	0.0007422	0.0194725	-0.922793
Srgap3	0.3788028	3.186433	4.0898759	0.0017837	0.0359091	-1.316956
Dgka	0.3811129	3.2183317	3.8586947	0.0026511	0.0462241	-1.723352
Nrp1	0.386994	7.9501705	8.5324791	3.481E-06	0.0005512	4.563938
Syngr1	0.3981129	6.6493454	7.2571044	1.614E-05	0.0013871	2.9822607
Fam217b	0.3991929	3.503902	4.4636195	0.0009529	0.0230693	-0.757502
Mef2a	0.4026858	8.4744362	5.1832897	0.0003005	0.0099865	-0.137506
Pkib	0.4037121	4.2421574	4.1834911	0.0015219	0.032364	-1.408156
Cd200r4	0.4055023	4.633488	4.6709394	0.0006784	0.0183577	-0.658934
S1pr1	0.4068146	6.7983233	3.885387	0.0025317	0.0452887	-2.319957
Cebpa	0.4072587	7.6384086	9.9326115	7.801E-07	0.0002539	6.1419269
Enc1	0.4082572	4.9920854	4.5939335	0.0007691	0.0197633	-0.85646
Pald1	0.4104709	5.1901146	3.9694926	0.0021908	0.0414556	-1.972076
Atp2b4	0.4182814	5.7019483	5.6979909	0.0001377	0.005932	0.822738
Hpgds	0.422489	6.8496674	4.1233845	0.001685	0.034673	-1.902743
Atp2a3	0.4236789	6.0726094	5.4939946	0.0001868	0.0073442	0.4565458
Bcl2l1	0.4291194	6.8379856	10.325267	5.287E-07	0.0001998	6.5697843
Noct	0.4303262	4.8971003	5.2298848	0.0002796	0.0095217	0.2114001
Dennd2c	0.4316326	4.0135538	7.4062373	1.336E-05	0.001203	3.5112152
Sulf2	0.4376354	5.0591056	5.9445768	9.601E-05	0.0046871	1.2975086
Maml3	0.4395749	3.6720235	5.2177444	0.0002849	0.0096482	0.4377288
Hpse	0.4406532	6.9562965	6.6012089	3.826E-05	0.0025328	2.0558994
Mthfs	0.440901	4.2425456	5.0548262	0.0003673	0.0115079	0.0553539
Tmem132a	0.4464576	3.9344227	4.7406296	0.000606	0.0169862	-0.393213
Cd72	0.4544796	5.9276421	4.4273644	0.0010119	0.0241471	-1.288241
Tmem273	0.4555091	4.9957737	4.9014265	0.0004682	0.0138187	-0.341684
Tcf7l2	0.456388	4.9134838	5.7714849	0.0001236	0.0055469	1.0591158
Fam117a	0.4714872	2.5661264	3.8776509	0.0025657	0.0456878	-1.538223
Pcp4l1	0.4832858	4.487948	3.8170742	0.0028489	0.0481588	-2.101258

6330403L08Rik	0.4846959	3.9542546	5.8043213	0.0001177	0.0053475	1.2880185
Pram1	0.4872017	3.6471554	4.7078622	0.000639	0.0174923	-0.381039
Pcyt1b	0.4928363	2.4808292	4.5096735	0.0008832	0.0216951	-0.46673
Itgb5	0.4946165	8.1639058	6.0347807	8.432E-05	0.0043331	1.1989592
Zc3hav1l	0.4959403	2.9115848	5.905645	0.0001016	0.0048185	1.6182451
Lpcat4	0.4960045	4.2573005	5.5974396	0.0001599	0.0065515	0.912752
Wwp1	0.4967666	6.9634467	4.4287522	0.0010096	0.0241408	-1.376717
Aplp1	0.4971838	2.3657898	3.8643315	0.0026254	0.0462241	-1.519548
Fcgrt	0.5010087	4.785301	4.6150119	0.0007431	0.0194725	-0.780272
Tcn2	0.5024303	6.5573114	4.98905	0.0004074	0.0124	-0.404929
Runx2	0.5089712	3.4137648	6.5359689	4.182E-05	0.0026773	2.4404071
Rap2a	0.5090893	7.6504623	12.245473	9.283E-08	5.725E-05	8.3684836
Trf	0.5106994	5.1245594	7.3759841	1.388E-05	0.0012322	3.3082681
Plekhg5	0.5125206	2.2314217	4.4598122	0.000959	0.0231671	-0.50846
Bmyc	0.5179565	3.2668962	6.7894046	2.97E-05	0.0020966	2.8044068
Loxl3	0.5274285	2.9947303	4.0713594	0.0018408	0.0365562	-1.300511
Chst3	0.5285344	3.8001806	5.4679402	0.0001943	0.007513	0.8058392
Pf4	0.5335207	5.5415429	6.5086965	4.34E-05	0.0027051	2.0572175
Il17ra	0.5354575	6.8710243	9.8712129	8.299E-07	0.0002628	6.0967205
Ccnd1	0.5441173	8.9985284	9.6694144	1.02E-06	0.0003063	5.8602132
Mrgpre	0.5486993	3.9988419	4.5944474	0.0007685	0.0197633	-0.648559
Arsb	0.5516904	7.9364362	7.6416751	9.971E-06	0.0009859	3.4534903
Axl	0.5549793	4.3240022	4.0684511	0.0018499	0.0366055	-1.62202
Gpr157	0.5605564	4.8050571	4.1988371	0.001483	0.0317669	-1.495242
Reps2	0.5655234	4.8525309	4.7798606	0.0005688	0.0160755	-0.515038
Plekhh2	0.5749352	4.2516203	6.5881882	3.894E-05	0.0025491	2.3759444
Lsp1	0.579549	5.7731993	5.6872692	0.0001399	0.0060048	0.7995115
Slc24a3	0.5815717	2.8788932	5.317447	0.0002443	0.0088839	0.7478783
Bcl2a1b	0.5817784	3.9098215	5.656871	0.0001464	0.0062136	1.076
Slc9a7	0.5840555	3.8544515	3.9702461	0.002188	0.0414556	-1.681789
Itgav	0.5878968	8.4462959	9.4526533	1.277E-06	0.0003124	5.6206498
Tnni2	0.5882376	2.0659213	4.6632121	0.000687	0.018532	-0.161306
Plaat3	0.5928575	3.6931373	6.8833758	2.622E-05	0.0019201	2.8740808
Cmtm8	0.594255	0.8978111	3.9244457	0.002367	0.0436074	-1.241086
Sesn3	0.5966875	2.8931876	4.0670479	0.0018543	0.0366055	-1.28359
Spp1	0.5990469	11.567805	5.7672946	0.0001243	0.0055599	0.8391753
Slc7a4	0.602182	3.0724854	5.5701817	0.0001666	0.0067301	1.1011221
Sema4c	0.6032733	3.3387121	5.3183486	0.000244	0.0088839	0.6681173
Icosl	0.6036986	3.8718121	5.632131	0.0001519	0.0063544	1.0460969
Smad7	0.6078385	5.1565565	8.1740902	5.262E-06	0.0006858	4.3168807
Ccl4	0.6134311	4.0128891	6.8133815	2.877E-05	0.0020744	2.7294389

Id1	0.6609055	6.4208698	13.845963	2.589E-08	3.885E-05	9.7158465
Fads6	0.6694618	1.6731734	4.3382923	0.0011735	0.0270138	-0.636459
Folr2	0.672209	5.598291	5.5255299	0.0001781	0.0070747	0.5731969
Apoc2	0.6790805	3.1013895	6.5404301	4.156E-05	0.0026773	2.4894216
Cd83	0.6830227	3.7421183	4.7809332	0.0005678	0.0160755	-0.275568
Myl6b	0.6871235	0.4341952	3.866815	0.0026142	0.0462241	-1.317684
Cd300ld3	0.6896377	5.5458248	5.2086327	0.000289	0.0097195	0.0755769
Dgkg	0.6900328	2.0679631	4.3023768	0.0012461	0.0281307	-0.739033
Stk26	0.6956544	2.5427559	4.5123129	0.0008794	0.021649	-0.469305
Gpr176	0.7094551	3.2337146	6.0227747	8.578E-05	0.0043511	1.7464335
Epha2	0.7145953	2.1426089	6.0294697	8.496E-05	0.0043388	1.8502576
Zranb3	0.7180504	6.7257514	17.813325	1.801E-09	7.818E-06	12.401315
Cxcl14	0.7184162	7.4698511	17.637602	2.002E-09	7.818E-06	12.294662
Slc40a1	0.7203218	6.3222998	4.7341106	0.0006124	0.017007	-0.805618
Enpp1	0.7221898	4.8648308	8.9521507	2.181E-06	0.0004327	5.2683665
Ctla2b	0.72887	5.9056806	8.0315911	6.225E-06	0.0007442	4.0544584
Galnt3	0.7363091	1.0074797	3.8265955	0.0028024	0.0478072	-1.402468
Thsd1	0.7410997	1.8461944	5.3917993	0.0002181	0.0081121	0.9573634
Epas1	0.7505307	4.137544	5.5017417	0.0001846	0.0072833	0.7960003
Hdac9	0.7631166	2.1121948	5.6185928	0.0001549	0.0064379	1.2755649
Zfp618	0.780652	3.1047483	7.1984554	1.74E-05	0.0014666	3.3557607
Gm42793	0.7913901	1.9007338	4.3432975	0.0011637	0.0268948	-0.650574
Arap2	0.7980741	4.4829194	8.1673152	5.304E-06	0.0006858	4.4018654
Folr1	0.8014715	0.4558366	4.1268957	0.001675	0.0346244	-0.921101
Fzd7	0.8042662	5.661756	8.8147774	2.537E-06	0.0004557	5.0220202
Gm38248	0.8045898	2.4361826	4.7968163	0.0005535	0.0158895	0.0062446
Zbtb16	0.8048636	-0.002931	3.9002633	0.0024676	0.0445161	-1.273031
Pmepa1	0.8171101	3.0661609	5.4693338	0.0001939	0.007513	0.9548173
Etv4	0.8530468	0.1018386	4.5630416	0.0008091	0.0202991	-0.308863
Jag2	0.8702198	0.823285	3.8278677	0.0027962	0.0478072	-1.389245
Plau	0.8800947	10.000834	5.348576	0.000233	0.0085567	0.1491814
Flt1	0.8805328	3.6208414	7.4658633	1.24E-05	0.0011351	3.6488115
Ndrg2	0.8807255	1.8072892	5.369795	0.0002255	0.008336	0.926639
Disc1	0.8875551	0.0047671	3.7850943	0.0030113	0.0497654	-1.445204
Rnase4	0.9020623	6.0951553	5.672153	0.0001431	0.0061185	0.7441951
Qpct	0.9353243	3.0822255	8.2979206	4.555E-06	0.0006426	4.6700593
Ccl7	0.9435931	4.5153422	4.0937227	0.001772	0.035798	-1.603073
Gm1673	0.9454576	1.4741674	4.2705491	0.0013144	0.0291719	-0.725522
Apoe	0.9544818	11.083291	8.3854337	4.117E-06	0.0006107	4.4200484
Smagp	0.9555856	2.2209193	3.8500192	0.0026911	0.0466445	-1.508518
F13a1	1.0051536	2.4975846	4.0347825	0.0019592	0.0380318	-1.24467

Sox4	1.0079346	1.8055062	6.0602771	8.129E-05	0.0042524	1.884657
Cx3cr1	1.017138	7.043542	11.715191	1.464E-07	7.148E-05	7.9096593
Gas6	1.0706733	7.6118843	5.3920798	0.000218	0.0081121	0.2090304
Cd28	1.0840602	3.0240337	4.8544591	0.0005047	0.0146727	0.0091612
Rasgrp3	1.085883	4.2355233	11.059702	2.637E-07	0.000103	7.462165
Gdf15	1.0942419	1.4863668	5.2688251	0.0002633	0.00924	0.7811048
Ang	1.105213	1.418795	6.0620034	8.109E-05	0.0042524	1.8549339
Gm33370	1.1175908	0.1628815	4.7998877	0.0005508	0.0158895	0.0216119
Ighm	1.1845132	0.6352248	5.1168167	0.0003333	0.0105843	0.5103483
Pde1c	1.2050596	2.925275	8.2680491	4.716E-06	0.00065	4.6301429
Cd244a	1.2308655	0.2471889	4.268232	0.0013195	0.0291719	-0.721238
Thbs1	1.2868357	4.7170972	8.1574532	5.365E-06	0.0006858	4.3728816
Adgrg3	1.2987897	1.3450022	6.0619827	8.11E-05	0.0042524	1.8425556
Ctla2a	1.3164968	0.9639594	4.6441488	0.0007086	0.018899	-0.139368
Chn2	1.3948213	0.5694791	3.807577	0.0028962	0.0484781	-1.410252
Serpine2	1.5098628	2.0826594	8.1371854	5.495E-06	0.0006858	4.3662432
Tpbgl	1.5377324	0.2154516	5.8807934	0.0001053	0.0049555	1.3859392
Ch25h	1.5672116	1.8857663	6.373941	5.226E-05	0.0031566	2.2935433
Ccnd2	1.7739726	0.5818454	5.6202038	0.0001546	0.0064379	1.1471431
Fcrls	1.8604079	2.0390524	3.8641119	0.0026264	0.0462241	-1.43088
Fosb	1.9438751	-0.307782	8.167381	5.303E-06	0.0006858	3.2547252
Gm49391	1.9561915	1.2129236	5.4557248	0.0001979	0.0075781	1.0172316

eated with bilirubin
after differential
e for baseline

Supplementary Table
48 hours as determine
adjusted p-value < 0.0

Category
GOTERM_BP_DIRECT
GOTERM_BP_DIRECT
GOTERM_BP_DIRECT
GOTERM_BP_DIRECT
GOTERM_BP_DIRECT
GOTERM_BP_DIRECT
GOTERM_BP_DIRECT
GOTERM_BP_DIRECT
GOTERM_BP_DIRECT
GOTERM_BP_DIRECT
GOTERM_BP_DIRECT
GOTERM_BP_DIRECT
GOTERM_BP_DIRECT
GOTERM_BP_DIRECT
GOTERM_BP_DIRECT
GOTERM_BP_DIRECT
GOTERM_BP_DIRECT
GOTERM_BP_DIRECT
GOTERM_BP_DIRECT
GOTERM_BP_DIRECT
GOTERM_BP_DIRECT
GOTERM_BP_DIRECT
GOTERM_BP_DIRECT
GOTERM_BP_DIRECT
GOTERM_BP_DIRECT
GOTERM_BP_DIRECT
GOTERM_BP_DIRECT
GOTERM_BP_DIRECT
GOTERM_BP_DIRECT
GOTERM_BP_DIRECT
GOTERM_BP_DIRECT
GOTERM_BP_DIRECT
GOTERM_BP_DIRECT
GOTERM_BP_DIRECT

GOTERM_BP_DIRECT
GOTERM_BP_DIRECT
GOTERM_BP_DIRECT
GOTERM_BP_DIRECT
GOTERM_BP_DIRECT
GOTERM_BP_DIRECT
GOTERM_BP_DIRECT
GOTERM_BP_DIRECT
GOTERM_BP_DIRECT
GOTERM_BP_DIRECT
GOTERM_BP_DIRECT
GOTERM_BP_DIRECT
GOTERM_BP_DIRECT
GOTERM_BP_DIRECT
GOTERM_BP_DIRECT
GOTERM_BP_DIRECT
GOTERM_BP_DIRECT
GOTERM_BP_DIRECT
GOTERM_BP_DIRECT
GOTERM_BP_DIRECT
GOTERM_BP_DIRECT
GOTERM_BP_DIRECT
GOTERM_BP_DIRECT
GOTERM_BP_DIRECT
GOTERM_BP_DIRECT
GOTERM_BP_DIRECT
GOTERM_BP_DIRECT
GOTERM_BP_DIRECT
GOTERM_BP_DIRECT
GOTERM_BP_DIRECT
GOTERM_BP_DIRECT
GOTERM_BP_DIRECT
GOTERM_BP_DIRECT
GOTERM_BP_DIRECT
GOTERM_BP_DIRECT
GOTERM_BP_DIRECT
GOTERM_BP_DIRECT
GOTERM_BP_DIRECT
GOTERM_BP_DIRECT
GOTERM_BP_DIRECT
GOTERM_BP_DIRECT

GOTERM_BP_DIRECT
GOTERM_BP_DIRECT
GOTERM_BP_DIRECT
GOTERM_BP_DIRECT

e 4. Gene Ontology Biological Processes altered in bone marrow-derived macro
ed by NIH DAVID. Assessed were differentially expressed genes decreased with
05 after normalization to each mouse as a covariate. Shown are the pathways w

Term
GO:0007165~signal transduction
GO:0007229~integrin-mediated signaling pathway
GO:0030335~positive regulation of cell migration
GO:0030595~leukocyte chemotaxis
GO:0007155~cell adhesion
GO:0001525~angiogenesis
GO:0006897~endocytosis
GO:0046718~viral entry into host cell
GO:0051897~positive regulation of protein kinase B signaling
GO:0043547~positive regulation of GTPase activity
GO:0045766~positive regulation of angiogenesis
GO:0030154~cell differentiation
GO:0006954~inflammatory response
GO:0007179~transforming growth factor beta receptor signaling pathway
GO:0040008~regulation of growth
GO:0046834~lipid phosphorylation
GO:0043524~negative regulation of neuron apoptotic process
GO:0030168~platelet activation
GO:0006909~phagocytosis
GO:0045780~positive regulation of bone resorption
GO:0006013~mannose metabolic process
GO:0043491~protein kinase B signaling
GO:0031589~cell-substrate adhesion
GO:0006935~chemotaxis
GO:0060412~ventricular septum morphogenesis
GO:2001237~negative regulation of extrinsic apoptotic signaling pathway
GO:0006654~phosphatidic acid biosynthetic process
GO:0032092~positive regulation of protein binding
GO:0042327~positive regulation of phosphorylation
GO:0006898~receptor-mediated endocytosis
GO:0034446~substrate adhesion-dependent cell spreading
GO:0001701~in utero embryonic development
GO:0000122~negative regulation of transcription from RNA polymerase II promoter

GO:0048844~artery morphogenesis
GO:0046486~glycerolipid metabolic process
GO:0007399~nervous system development
GO:1900026~positive regulation of substrate adhesion-dependent cell spreading
GO:0033690~positive regulation of osteoblast proliferation
GO:0071466~cellular response to xenobiotic stimulus
GO:0051651~maintenance of location in cell
GO:0030195~negative regulation of blood coagulation
GO:0046339~diacylglycerol metabolic process
GO:0005975~carbohydrate metabolic process
GO:0006915~apoptotic process
GO:0010763~positive regulation of fibroblast migration
GO:0010862~positive regulation of pathway-restricted SMAD protein phosphorylation
GO:0001958~endochondral ossification
GO:0048545~response to steroid hormone
GO:0030308~negative regulation of cell growth
GO:0045944~positive regulation of transcription from RNA polymerase II promoter
GO:0045669~positive regulation of osteoblast differentiation
GO:0060326~cell chemotaxis
GO:0007040~lysosome organization
GO:0071222~cellular response to lipopolysaccharide
GO:0043542~endothelial cell migration
GO:0002063~chondrocyte development
GO:0051044~positive regulation of membrane protein ectodomain proteolysis
GO:2000503~positive regulation of natural killer cell chemotaxis
GO:0048661~positive regulation of smooth muscle cell proliferation
GO:0007160~cell-matrix adhesion
GO:0006796~phosphate-containing compound metabolic process
GO:0048469~cell maturation
GO:0030155~regulation of cell adhesion
GO:0030182~neuron differentiation
GO:0043277~apoptotic cell clearance
GO:0033627~cell adhesion mediated by integrin
GO:0016477~cell migration
GO:1903077~negative regulation of protein localization to plasma membrane
GO:0045807~positive regulation of endocytosis
GO:0048260~positive regulation of receptor-mediated endocytosis
GO:0007596~blood coagulation
GO:0051056~regulation of small GTPase mediated signal transduction
GO:0030316~osteoclast differentiation
GO:0030217~T cell differentiation

GO:0006517~protein deglycosylation
GO:0048512~circadian behavior
GO:1900121~negative regulation of receptor binding
GO:0010757~negative regulation of plasminogen activation

phages treated with bilirubin for
bilirubin treatment with an
ith a p-value < 0.01.

Count	%	PValue
56	13.493976	3.98E-08
14	3.373494	2.85E-07
19	4.5783133	4.82E-06
6	1.4457831	4.97E-06
29	6.9879518	7.56E-06
18	4.3373494	1.31E-05
16	3.8554217	2.62E-05
11	2.6506024	3.30E-05
12	2.8915663	4.96E-05
14	3.373494	9.88E-05
13	3.1325301	1.01E-04
30	7.2289157	1.11E-04
22	5.3012048	1.26E-04
10	2.4096386	1.71E-04
8	1.9277108	1.89E-04
5	1.2048193	2.23E-04
12	2.8915663	2.42E-04
8	1.9277108	3.73E-04
8	1.9277108	4.47E-04
5	1.2048193	4.53E-04
4	0.9638554	5.81E-04
7	1.686747	5.87E-04
5	1.2048193	6.77E-04
10	2.4096386	7.38E-04
6	1.4457831	7.46E-04
6	1.4457831	7.46E-04
6	1.4457831	7.46E-04
8	1.9277108	7.97E-04
6	1.4457831	8.45E-04
8	1.9277108	8.61E-04
7	1.686747	9.53E-04
13	3.1325301	0.0011363
35	8.4337349	0.001144

5	1.2048193	0.0013455
4	0.9638554	0.0014571
19	4.5783133	0.0016822
6	1.4457831	0.0018388
4	0.9638554	0.0018667
7	1.686747	0.0020269
3	0.7228916	0.002257
4	0.9638554	0.0023413
4	0.9638554	0.0023413
11	2.6506024	0.0025056
24	5.7831325	0.0026465
4	0.9638554	0.0028843
6	1.4457831	0.0029437
5	1.2048193	0.0030502
4	0.9638554	0.0034985
9	2.1686747	0.0034997
39	9.3975904	0.0035359
7	1.686747	0.0038322
7	1.686747	0.0038322
6	1.4457831	0.0044625
11	2.6506024	0.0045
5	1.2048193	0.0047723
4	0.9638554	0.0049515
4	0.9638554	0.0049515
3	0.7228916	0.0054968
6	1.4457831	0.0056037
8	1.9277108	0.0057921
4	0.9638554	0.0057951
5	1.2048193	0.0064246
6	1.4457831	0.00647
10	2.4096386	0.0066305
4	0.9638554	0.0067194
5	1.2048193	0.0084178
13	3.1325301	0.008577
4	0.9638554	0.0088177
4	0.9638554	0.0088177
4	0.9638554	0.0088177
7	1.686747	0.0091521
8	1.9277108	0.009229
5	1.2048193	0.0099488
5	1.2048193	0.0099488

3	0.7228916	0.0099965
3	0.7228916	0.0099965
3	0.7228916	0.0099965
3	0.7228916	0.0099965

Genes
5140, 6351, 5141, 7046, 9547, 6354, 9467, 2034, 23048, 10019, 399, 2787, 2621, 83478, 5137
8751, 3690, 5341, 3693, 7006, 101, 81794, 84106, 3909, 3685, 5786, 3655, 3688, 8829
3082, 7046, 7057, 8650, 6354, 7852, 10855, 1601, 1512, 79567, 2321, 3685, 54910, 3655, 368
1524, 5196, 1269, 1880, 1130, 1901
6351, 9486, 6696, 9404, 2350, 971, 333, 29780, 56114, 976, 10154, 6347, 3655, 3636, 7057, 3
5140, 8751, 154796, 8742, 2034, 3397, 283, 101, 388, 58504, 57125, 2321, 83478, 3685, 7855
4041, 3092, 9711, 5167, 4035, 29967, 23048, 257364, 6272, 9185, 54453, 333, 598, 8527, 846
3690, 950, 2621, 3693, 7852, 3685, 558, 3688, 1969, 11059, 8829
10855, 3092, 1524, 7057, 101, 7046, 2621, 940, 558, 9518, 3688, 6934
6351, 6001, 6354, 8874, 81704, 1124, 25780, 116984, 55357, 6347, 5996, 3655, 3688, 1901
3082, 3690, 7057, 7048, 8742, 10855, 1524, 1512, 388, 2321, 3688, 947, 8829
7048, 6256, 4092, 2768, 57447, 51460, 2321, 83478, 54910, 4205, 4208, 3714, 6925, 3092, 63
6351, 5196, 4064, 7057, 6696, 9734, 6354, 7852, 3340, 2350, 23765, 10855, 1524, 101, 51284
7046, 4054, 6498, 5045, 7048, 9404, 3693, 3397, 9518, 4092
166, 598, 7048, 2621, 51542, 6414, 29967, 64393
64781, 8527, 1608, 8525, 1606
595, 54463, 101, 598, 84678, 348, 558, 6347, 3572, 5538, 8829, 4208
5196, 3690, 558, 8527, 1608, 8525, 1606, 5270
5031, 2621, 9711, 4035, 22918, 558, 1130, 3688
101, 3690, 4054, 6696, 169200
4122, 4124, 4125, 23324
83667, 2621, 940, 558, 6347, 1263, 1969
3690, 2621, 3685, 3655, 3688
1524, 154796, 6354, 9547, 4046, 7852, 6347, 152189, 5328, 1901
7046, 7048, 659, 161882, 6659, 4092
7057, 7046, 3685, 3655, 6934, 8829
51099, 254531, 8527, 1608, 8525, 1606
4035, 340061, 54972, 8874, 10211, 3077, 6934, 3955
7057, 2321, 8324, 3655, 283, 8829
1601, 950, 7048, 4035, 348, 26119, 5538, 10487
3690, 29780, 9404, 8324, 3685, 558, 8829
8178, 7046, 7189, 7048, 154796, 4124, 2768, 598, 51542, 54855, 7703, 3688, 3714
8553, 7189, 6498, 6256, 1263, 23466, 4092, 81628, 57600, 595, 9935, 79618, 4205, 4208, 259

7046, 7048, 348, 4092, 8829
8527, 1608, 8525, 1606
25814, 7046, 8874, 64207, 382, 81544, 57447, 333, 51232, 1200, 56114, 558, 54910, 4205, 55
10855, 1601, 3690, 7016, 8874, 8829
10855, 4041, 3690, 3685
5141, 3690, 2621, 7852, 3297, 5911, 4208
348, 3074, 3073
348, 947, 5270, 5627
8527, 1608, 8525, 1606
80267, 2720, 9469, 57134, 4122, 4124, 2683, 4125, 2591, 3074, 3073
3092, 7046, 7057, 25959, 7048, 8742, 7852, 1263, 30061, 26999, 1601, 1512, 388, 333, 25548
7057, 3690, 8874, 3688
1601, 7046, 7048, 9518, 659, 3077
860, 54928, 4665, 4208, 3636
1601, 6696, 7048, 164091
1601, 83667, 8751, 1026, 5167, 659, 5538, 5270, 51094
3082, 7189, 6256, 2034, 29109, 30061, 51284, 9935, 7855, 659, 3655, 4205, 4800, 4208, 6925
3082, 4041, 860, 659, 3572, 1050, 4208
3082, 1524, 9547, 9732, 7852, 51411, 1969
79158, 411, 1200, 1130, 3074, 3073
1524, 5196, 4064, 7189, 22904, 23097, 558, 6347, 3297, 3654, 4208
7046, 57449, 9404, 8742, 8829
860, 54928, 659, 55959
101, 5045, 348, 257364
6351, 6354, 9547
7057, 3690, 7048, 3398, 3654, 1901
10855, 8751, 3690, 3693, 3685, 3655, 3688, 947
64077, 5167, 5789, 3635
860, 2034, 558, 7855, 1050
9267, 3909, 7852, 5328, 1901, 3714
595, 860, 9734, 8324, 7855, 3397, 5789, 1969, 4208, 1901
3690, 2621, 4035, 3685
3690, 3693, 3685, 3655, 3688
3690, 7057, 3693, 283, 388, 3909, 2321, 3685, 558, 8525, 3688, 1969, 1901
1601, 8650, 598, 6248
1601, 4035, 348, 10211
3092, 3077, 26119, 5538
2768, 3690, 2621, 5328, 2162, 5270, 5627
58504, 57449, 154796, 1124, 116984, 8874, 9901, 81704
7189, 22904, 1880, 1969, 169200
860, 399, 6659, 3955, 3714

4122, 4124, 4125
80045, 11245, 9099
8751, 489, 3077
7057, 5328, 5270

List Total	Pop Hits	Pop Total	Fold Enrichment	Bonferroni	Benjamini	FDR
383	1283	19414	2.212471179	1.03E-04	1.03E-04	1.02E-04
383	111	19414	6.393244419	7.40E-04	3.70E-04	3.65E-04
383	262	19414	3.675941243	0.0124353	0.0032279	0.0031807
383	14	19414	21.72398359	0.0128286	0.0032279	0.0031807
383	563	19414	2.610993883	0.0194501	0.0039283	0.0038709
383	255	19414	3.578067885	0.0334172	0.0056647	0.0055818
383	216	19414	3.754762595	0.0656984	0.0097079	0.0095659
383	102	19414	5.466492602	0.0821532	0.0107154	0.0105587
383	129	19414	4.715283259	0.1208188	0.0143068	0.0140975
383	189	19414	3.754762595	0.2263402	0.0239463	0.023596
383	164	19414	4.018053875	0.2315828	0.0239463	0.023596
383	690	19414	2.203882393	0.2499371	0.0239652	0.0236147
383	428	19414	2.605524511	0.279494	0.0252139	0.0248451
383	101	19414	5.018742083	0.3583066	0.0316862	0.0312227
383	61	19414	6.647776399	0.3880853	0.0327411	0.0322622
383	16	19414	15.8404047	0.4392547	0.0361515	0.0356227
383	154	19414	3.949815198	0.466585	0.0369635	0.0364229
383	68	19414	5.963446475	0.6210648	0.0539005	0.0531121
383	70	19414	5.79306229	0.686633	0.0587814	0.0579216
383	19	19414	13.33928817	0.6914568	0.0587814	0.0579216
383	9	19414	22.52857557	0.7792542	0.0693117	0.0682979
383	53	19414	6.694812552	0.7824453	0.0693117	0.0682979
383	21	19414	12.06887977	0.8280115	0.0717773	0.0707274
383	123	19414	4.121080897	0.8529508	0.0717773	0.0707274
383	37	19414	8.219885682	0.8561106	0.0717773	0.0707274
383	37	19414	8.219885682	0.8561106	0.0717773	0.0707274
383	37	19414	8.219885682	0.8561106	0.0717773	0.0707274
383	77	19414	5.266420264	0.8738732	0.0739158	0.0728347
383	38	19414	8.003572901	0.8888584	0.0745316	0.0734414
383	78	19414	5.198902055	0.8932121	0.0745316	0.0734414
383	58	19414	6.117673539	0.9160145	0.0798687	0.0787005
383	214	19414	3.079256241	0.9478575	0.0900611	0.0887438
383	988	19414	1.795673407	0.948887	0.0900611	0.0887438

383	25	19414	10.13785901	0.9697402	0.1028112	0.1013075
383	12	19414	16.89643168	0.977364	0.1081558	0.1065738
383	418	19414	2.304058865	0.9873997	0.1213988	0.1196231
383	45	19414	6.758572672	0.9916179	0.1276224	0.1257557
383	13	19414	15.59670617	0.9922043	0.1276224	0.1257557
383	67	19414	5.295896497	0.9948626	0.135022	0.1330471
383	4	19414	38.01697128	0.997178	0.1448278	0.1427095
383	14	19414	14.48265573	0.9977344	0.1448278	0.1427095
383	14	19414	14.48265573	0.9977344	0.1448278	0.1427095
383	175	19414	3.18618426	0.9985228	0.1513827	0.1491685
383	616	19414	1.974907599	0.9989768	0.1562666	0.1539809
383	15	19414	13.51714534	0.9994492	0.1662541	0.1638223
383	50	19414	6.082715405	0.9995282	0.1662541	0.1638223
383	31	19414	8.175692748	0.9996425	0.1686034	0.1661373
383	16	19414	12.67232376	0.9998889	0.1837238	0.1810365
383	126	19414	3.620663931	0.9998892	0.1837238	0.1810365
383	1222	19414	1.617743459	0.9998992	0.1837238	0.1810365
383	76	19414	4.668750859	0.9999535	0.1914608	0.1886603
383	76	19414	4.668750859	0.9999535	0.1914608	0.1886603
383	55	19414	5.529741277	0.999991	0.2165016	0.2133349
383	190	19414	2.934643397	0.9999919	0.2165016	0.2133349
383	35	19414	7.241327863	0.999996	0.2254265	0.2221293
383	18	19414	11.26428779	0.9999975	0.2256842	0.2223832
383	18	19414	11.26428779	0.9999975	0.2256842	0.2223832
383	6	19414	25.34464752	0.9999994	0.2462174	0.2426161
383	58	19414	5.243720176	0.9999995	0.2467509	0.2431417
383	109	19414	3.720315232	0.9999997	0.2468126	0.2432026
383	19	19414	10.67143053	0.9999997	0.2468126	0.2432026
383	38	19414	6.669644084	0.9999999	0.2668104	0.2629078
383	60	19414	5.068929504	1	0.2668104	0.2629078
383	170	19414	2.981723238	1	0.2685709	0.2646426
383	20	19414	10.13785901	1	0.2685709	0.2646426
383	41	19414	6.181621346	1	0.3272636	0.3224768
383	275	19414	2.39622122	1	0.3272636	0.3224768
383	22	19414	9.216235462	1	0.3272636	0.3224768
383	22	19414	9.216235462	1	0.3272636	0.3224768
383	22	19414	9.216235462	1	0.3272636	0.3224768
383	91	19414	3.899176541	1	0.3324912	0.3276279
383	119	19414	3.4076837	1	0.3324912	0.3276279
383	43	19414	5.894104074	1	0.3324912	0.3276279
383	43	19414	5.894104074	1	0.3324912	0.3276279

383	8	19414	19.00848564	1	0.3324912	0.3276279
383	8	19414	19.00848564	1	0.3324912	0.3276279
383	8	19414	19.00848564	1	0.3324912	0.3276279
383	8	19414	19.00848564	1	0.3324912	0.3276279

SupplementalryTable
48 hours as determine
adjusted p-value < 0.0

Category
GOTERM_BP_DIRECT
GOTERM_BP_DIRECT
GOTERM_BP_DIRECT
GOTERM_BP_DIRECT
GOTERM_BP_DIRECT
GOTERM_BP_DIRECT
GOTERM_BP_DIRECT
GOTERM_BP_DIRECT
GOTERM_BP_DIRECT
GOTERM_BP_DIRECT
GOTERM_BP_DIRECT
GOTERM_BP_DIRECT
GOTERM_BP_DIRECT
GOTERM_BP_DIRECT
GOTERM_BP_DIRECT
GOTERM_BP_DIRECT
GOTERM_BP_DIRECT
GOTERM_BP_DIRECT
GOTERM_BP_DIRECT
GOTERM_BP_DIRECT
GOTERM_BP_DIRECT
GOTERM_BP_DIRECT
GOTERM_BP_DIRECT
GOTERM_BP_DIRECT
GOTERM_BP_DIRECT
GOTERM_BP_DIRECT
GOTERM_BP_DIRECT
GOTERM_BP_DIRECT
GOTERM_BP_DIRECT
GOTERM_BP_DIRECT
GOTERM_BP_DIRECT
GOTERM_BP_DIRECT
GOTERM_BP_DIRECT
GOTERM_BP_DIRECT

GOTERM_BP_DIRECT
GOTERM_BP_DIRECT
GOTERM_BP_DIRECT
GOTERM_BP_DIRECT
GOTERM_BP_DIRECT
GOTERM_BP_DIRECT
GOTERM_BP_DIRECT
GOTERM_BP_DIRECT
GOTERM_BP_DIRECT
GOTERM_BP_DIRECT
GOTERM_BP_DIRECT
GOTERM_BP_DIRECT
GOTERM_BP_DIRECT
GOTERM_BP_DIRECT
GOTERM_BP_DIRECT
GOTERM_BP_DIRECT
GOTERM_BP_DIRECT
GOTERM_BP_DIRECT
GOTERM_BP_DIRECT
GOTERM_BP_DIRECT
GOTERM_BP_DIRECT
GOTERM_BP_DIRECT
GOTERM_BP_DIRECT
GOTERM_BP_DIRECT
GOTERM_BP_DIRECT
GOTERM_BP_DIRECT
GOTERM_BP_DIRECT
GOTERM_BP_DIRECT
GOTERM_BP_DIRECT
GOTERM_BP_DIRECT
GOTERM_BP_DIRECT
GOTERM_BP_DIRECT
GOTERM_BP_DIRECT
GOTERM_BP_DIRECT
GOTERM_BP_DIRECT
GOTERM_BP_DIRECT
GOTERM_BP_DIRECT
GOTERM_BP_DIRECT
GOTERM_BP_DIRECT
GOTERM_BP_DIRECT
GOTERM_BP_DIRECT

GOTERM_BP_DIRECT
GOTERM_BP_DIRECT
GOTERM_BP_DIRECT
GOTERM_BP_DIRECT
GOTERM_BP_DIRECT
GOTERM_BP_DIRECT
GOTERM_BP_DIRECT
GOTERM_BP_DIRECT
GOTERM_BP_DIRECT
GOTERM_BP_DIRECT
GOTERM_BP_DIRECT
GOTERM_BP_DIRECT
GOTERM_BP_DIRECT

e 3. Gene Ontology Biological Processes altered in bone marrow-derived macro
ed by NIH DAVID. Assessed were differentially expressed genes increased with
)5 after normalization to each mouse as a covariate. Shown are the pathways w

Term
GO:0001525~angiogenesis
GO:0030335~positive regulation of cell migration
GO:0006915~apoptotic process
GO:0007165~signal transduction
GO:0051897~positive regulation of phosphatidylinositol 3-kinase/protein kinase B signal tran
GO:0007399~nervous system development
GO:0030154~cell differentiation
GO:0045893~positive regulation of DNA-templated transcription
GO:0043066~negative regulation of apoptotic process
GO:0001958~endochondral ossification
GO:0006898~receptor-mediated endocytosis
GO:0030595~leukocyte chemotaxis
GO:0046849~bone remodeling
GO:0006654~phosphatidic acid biosynthetic process
GO:0000122~negative regulation of transcription by RNA polymerase II
GO:0046834~lipid phosphorylation
GO:0007200~phospholipase C-activating G protein-coupled receptor signaling pathway
GO:0007155~cell adhesion
GO:0006955~immune response
GO:0006954~inflammatory response
GO:0046339~diacylglycerol metabolic process
GO:0045944~positive regulation of transcription by RNA polymerase II
GO:0006897~endocytosis
GO:0046486~glycerolipid metabolic process
GO:0030282~bone mineralization
GO:0042327~positive regulation of phosphorylation
GO:0043524~negative regulation of neuron apoptotic process
GO:0043065~positive regulation of apoptotic process
GO:0008654~phospholipid biosynthetic process
GO:0001501~skeletal system development
GO:0043537~negative regulation of blood vessel endothelial cell migration
GO:0043277~apoptotic cell clearance
GO:0010976~positive regulation of neuron projection development

GO:0030207~chondroitin sulfate proteoglycan catabolic process
GO:0060392~negative regulation of SMAD protein signal transduction
GO:0048260~positive regulation of receptor-mediated endocytosis
GO:0051056~regulation of small GTPase mediated signal transduction
GO:0016477~cell migration
GO:0030182~neuron differentiation
GO:0042159~lipoprotein catabolic process
GO:0008203~cholesterol metabolic process
GO:0007507~heart development
GO:0048844~artery morphogenesis
GO:0030217~T cell differentiation
GO:0070371~ERK1 and ERK2 cascade
GO:0010757~negative regulation of plasminogen activation
GO:0007229~integrin-mediated signaling pathway
GO:0045766~positive regulation of angiogenesis
GO:0010467~gene expression
GO:0006874~intracellular calcium ion homeostasis
GO:0048512~circadian behavior
GO:0045589~regulation of regulatory T cell differentiation
GO:0045807~positive regulation of endocytosis
GO:0042593~glucose homeostasis
GO:0021510~spinal cord development
GO:0001701~in utero embryonic development
GO:0032940~secretion by cell
GO:0038060~nitric oxide-cGMP-mediated signaling
GO:2000320~negative regulation of T-helper 17 cell differentiation
GO:0000082~G1/S transition of mitotic cell cycle
GO:0048514~blood vessel morphogenesis
GO:0030032~lamellipodium assembly
GO:0001818~negative regulation of cytokine production
GO:0030168~platelet activation
GO:0001975~response to amphetamine
GO:0008284~positive regulation of cell population proliferation
GO:0030308~negative regulation of cell growth
GO:0043542~endothelial cell migration
GO:0007596~blood coagulation
GO:0060444~branching involved in mammary gland duct morphogenesis
GO:0030195~negative regulation of blood coagulation
GO:0008285~negative regulation of cell population proliferation
GO:0010629~negative regulation of gene expression
GO:0071466~cellular response to xenobiotic stimulus

GO:0033690~positive regulation of osteoblast proliferation
GO:2001237~negative regulation of extrinsic apoptotic signaling pathway
GO:0007167~enzyme-linked receptor protein signaling pathway
GO:1903671~negative regulation of sprouting angiogenesis
GO:2000669~negative regulation of dendritic cell apoptotic process
GO:0035313~wound healing, spreading of epidermal cells
GO:0060412~ventricular septum morphogenesis
GO:0050679~positive regulation of epithelial cell proliferation
GO:0040037~negative regulation of fibroblast growth factor receptor signaling pathway
GO:0030336~negative regulation of cell migration
GO:0034446~substrate adhesion-dependent cell spreading
GO:0030512~negative regulation of transforming growth factor beta receptor signaling pathv
GO:0001764~neuron migration

phages treated with bilirubin for
bilirubin treatment with an
ith a p-value < 0.01.

Count	%	PValue
17	6.614786	3.20E-09
16	6.2256809	9.07E-09
22	8.5603113	2.47E-08
26	10.116732	2.89E-07
13	5.0583658	3.92E-07
14	5.4474708	3.49E-06
20	7.7821012	3.72E-06
21	8.1712062	1.05E-05
19	7.3929961	1.42E-05
6	2.3346304	1.78E-05
7	2.7237354	1.90E-05
5	1.9455253	2.07E-05
5	1.9455253	3.70E-05
5	1.9455253	3.70E-05
23	8.9494163	4.14E-05
4	1.5564202	6.60E-05
8	3.1128405	6.92E-05
15	5.8365759	1.42E-04
14	5.4474708	1.82E-04
13	5.0583658	1.86E-04
4	1.5564202	1.95E-04
25	9.7276265	2.09E-04
9	3.5019455	2.13E-04
4	1.5564202	2.43E-04
6	2.3346304	2.64E-04
5	1.9455253	3.10E-04
9	3.5019455	3.67E-04
12	4.6692607	5.75E-04
5	1.9455253	6.37E-04
7	2.7237354	7.39E-04
4	1.5564202	7.87E-04
4	1.5564202	8.99E-04
8	3.1128405	9.60E-04

	3	1.1673152	0.0010181
	4	1.5564202	0.0010214
	4	1.5564202	0.001296
	4	1.5564202	0.001296
	10	3.8910506	0.0013384
	9	3.5019455	0.0013537
	3	1.1673152	0.0014175
	6	2.3346304	0.0016074
	10	3.8910506	0.0016091
	4	1.5564202	0.0017881
	5	1.9455253	0.0018084
	5	1.9455253	0.0018084
	3	1.1673152	0.0018796
	6	2.3346304	0.0023381
	7	2.7237354	0.0023752
	11	4.2801556	0.0028042
	6	2.3346304	0.0029432
	3	1.1673152	0.0029876
	3	1.1673152	0.0029876
	4	1.5564202	0.0030871
	7	2.7237354	0.0031078
	4	1.5564202	0.0033472
	10	3.8910506	0.0035014
	3	1.1673152	0.0036315
	3	1.1673152	0.0036315
	3	1.1673152	0.0036315
	5	1.9455253	0.0037983
	4	1.5564202	0.0039066
	4	1.5564202	0.0039066
	4	1.5564202	0.0042063
	4	1.5564202	0.0045197
	4	1.5564202	0.0045197
	13	5.0583658	0.0049675
	6	2.3346304	0.0051
	4	1.5564202	0.0055429
	5	1.9455253	0.0058936
	3	1.1673152	0.0059102
	3	1.1673152	0.0059102
	10	3.8910506	0.0063577
	10	3.8910506	0.0065438
	5	1.9455253	0.0066466

3	1.1673152	0.0067821
4	1.5564202	0.0075351
3	1.1673152	0.0077086
3	1.1673152	0.0077086
3	1.1673152	0.0077086
3	1.1673152	0.0077086
4	1.5564202	0.0079778
5	1.9455253	0.0080383
3	1.1673152	0.0086886
6	2.3346304	0.0088258
4	1.5564202	0.0089083
5	1.9455253	0.0089586
6	2.3346304	0.0098109

Genes
17064, 18186, 14254, 18792, 16410, 239027, 21825, 21812, 21944, 13836, 13819, 11727, 136
20353, 14254, 18792, 18550, 16410, 18222, 107895, 21825, 20306, 21812, 20303, 13836, 136
70415, 215114, 17258, 16332, 17260, 21825, 22401, 21812, 26362, 11803, 59126, 14456, 127
18575, 217303, 20750, 70031, 259302, 22034, 19262, 227333, 57266, 14456, 104418, 54519,
13051, 19128, 14254, 15442, 21825, 21416, 23886, 21812, 26362, 12487, 14456, 16412, 5461
244667, 17258, 20353, 18222, 19063, 17260, 29811, 21812, 79221, 20677, 26362, 54138, 541
20720, 16450, 215114, 17258, 20353, 67469, 14254, 17131, 239027, 104156, 17260, 29811, 2
107771, 17258, 20750, 235320, 18044, 12393, 104156, 17260, 21416, 21812, 21953, 20677, 1
20750, 16971, 19063, 21825, 21812, 26362, 11727, 14456, 12795, 12575, 12045, 15259, 1244
12393, 16332, 18605, 17260, 17937, 14425
217303, 11816, 15442, 16971, 19063, 12331, 100017
13051, 56744, 12802, 13609, 20303
13836, 16998, 18605, 22034, 16973
227333, 67469, 110197, 104418, 13139
69930, 22658, 107771, 17258, 235320, 17131, 104156, 22034, 22761, 17260, 21416, 20602, 7
227333, 110197, 104418, 13139
269604, 227333, 13051, 16971, 13609, 110197, 104418, 13139
13051, 20750, 14276, 15442, 16410, 16332, 14275, 21825, 170736, 239393, 11803, 13836, 16
13051, 619441, 14132, 22034, 21825, 20306, 21944, 20303, 56744, 57266, 12487, 18605, 130
13051, 16950, 16410, 279572, 21825, 16172, 20306, 79221, 20303, 56744, 26362, 12802, 138
227333, 110197, 104418, 13139
107771, 12393, 22034, 17260, 20677, 13609, 16973, 18612, 53945, 16658, 69930, 14282, 172
227333, 11803, 215114, 16971, 16332, 19063, 194590, 16973, 239393
227333, 110197, 104418, 13139
20750, 12393, 94249, 16998, 18605, 21416
18186, 22041, 11727, 14369, 21825
26362, 11816, 18186, 16971, 19063, 12443, 66270, 12048, 17260
78334, 13033, 235320, 54126, 12045, 12048, 16412, 11852, 22401, 21812, 21416, 20677
99010, 15212, 67469, 71780, 225845
16450, 13836, 235320, 15212, 12393, 16998, 21812
11816, 17260, 21825, 381290
26362, 16410, 16971, 14456
20720, 244667, 13803, 11816, 16971, 11881, 16412, 19735

15212, 50917, 11881
65112, 16971, 17131, 23886
215114, 22041, 19063, 100017
69993, 239027, 232201, 231532
26362, 13836, 14254, 16410, 11727, 13609, 21825, 16412, 16419, 239393
13836, 12393, 13609, 12443, 17260, 16412, 15901, 79221, 20677
11813, 11816, 13033
12606, 11816, 16971, 12642, 16973, 100017
17258, 18186, 18605, 12575, 22761, 17260, 21812, 15901, 79221, 20677
11816, 18186, 17131, 21812
16450, 107568, 12393, 18605, 20677
107771, 22041, 16410, 16332, 13036
20720, 18792, 21825
378460, 18186, 16410, 16412, 19262, 16419
13051, 18186, 14254, 21825, 16412, 13036, 11852
11816, 13819, 12393, 16332, 18605, 17260, 16973, 20602, 14425, 16658, 20677
20750, 11816, 15212, 53313, 94249, 381290
269604, 53376, 381413
12487, 13024, 16923
11816, 16971, 16019, 16412
12606, 75747, 72674, 18605, 21416, 20602, 20677
72324, 16950, 18605, 20677
16450, 22658, 12575, 22034, 12048, 16412, 21812, 21416, 20602, 16658
20720, 26362, 18550
11816, 21825, 381290
56744, 17131, 319817
107771, 12443, 12795, 12444, 16412
13836, 14254, 21825, 16973
54126, 13609, 170736, 16412
26362, 13836, 29811, 79221
20720, 16440, 56744, 26362
14282, 67865, 19735, 79221
21985, 14276, 14254, 18792, 16410, 12393, 21825, 21812, 20677, 13609, 12443, 12444, 1303
20720, 72674, 19063, 18605, 12575, 22401
18186, 16410, 269608, 21812
20720, 19128, 14456, 74145, 21824
13836, 16973, 18612
20720, 11816, 21824
20720, 12606, 235320, 16332, 12575, 21825, 16412, 14367, 16923, 20677
11816, 16971, 16332, 94249, 12487, 12575, 17260, 15901, 381290, 12457
107771, 14456, 17260, 76108, 20303

15442, 16410, 16973
18186, 16410, 21812, 21416
26362, 16971, 14456
14254, 21825, 16923
26362, 14456, 12048
16410, 16419, 231532
17131, 22761, 21812, 20677
107771, 12393, 12444, 21416, 15901
72043, 21825, 21416
13051, 70415, 259302, 76108, 11852, 21812
26362, 16410, 14369, 170736
65112, 16971, 17131, 380664, 23886
244667, 26362, 18186, 14456, 17260, 239393

List Total	Pop Hits	Pop Total	Fold Enrichment	Bonferroni	Benjamini	FDR
249	288	29712	7.043507363	6.56E-06	6.56E-06	6.37E-06
249	268	29712	7.123898579	1.86E-05	9.31E-06	9.04E-06
249	583	29712	4.502841555	5.07E-05	1.69E-05	1.64E-05
249	925	29712	3.354008466	5.94E-04	1.48E-04	1.44E-04
249	221	29712	7.019135365	8.04E-04	1.61E-04	1.56E-04
249	321	29712	5.204218744	0.0071371	0.0010898	0.0010585
249	665	29712	3.588730863	0.0075994	0.0010898	0.0010585
249	782	29712	3.204387884	0.0214138	0.0027058	0.002628
249	666	29712	3.40417526	0.0287731	0.0032439	0.0031507
249	39	29712	18.35773865	0.0358218	0.003535	0.0034334
249	66	29712	12.65571376	0.038304	0.003535	0.0034334
249	20	29712	29.8313253	0.0415333	0.003535	0.0034334
249	23	29712	25.94028287	0.0732013	0.0054298	0.0052738
249	23	29712	25.94028287	0.0732013	0.0054298	0.0052738
249	1005	29712	2.730827789	0.0814615	0.0056646	0.0055019
249	10	29712	47.73012048	0.1267585	0.0083627	0.0081223
249	120	29712	7.95502008	0.1325265	0.0083627	0.0081223
249	523	29712	3.422331775	0.253616	0.0162497	0.0157827
249	471	29712	3.54682424	0.3117575	0.0190556	0.018508
249	409	29712	3.792735735	0.3171668	0.0190556	0.018508
249	14	29712	34.0929432	0.3304361	0.0190556	0.018508
249	1284	29712	2.323311939	0.3491247	0.0190556	0.018508
249	191	29712	5.622658172	0.3548846	0.0190556	0.018508
249	15	29712	31.82008032	0.3924585	0.0207614	0.0201648
249	68	29712	10.52870305	0.4187638	0.0217011	0.0210774
249	39	29712	15.29811554	0.470576	0.0244564	0.0237536
249	207	29712	5.188056574	0.5291066	0.0278883	0.0270869
249	399	29712	3.588730863	0.6932192	0.0421886	0.0409762
249	47	29712	12.69418098	0.7299555	0.0451293	0.0438324
249	128	29712	6.52560241	0.7808568	0.0505823	0.0491286
249	22	29712	21.69550931	0.8012768	0.0521034	0.0506061
249	23	29712	20.7522263	0.842277	0.0576901	0.0560322
249	185	29712	5.160013025	0.8608933	0.0597444	0.0580275

249	6	29712	59.6626506	0.8764607	0.0599104	0.0581887
249	24	29712	19.8875502	0.8772906	0.0599104	0.0581887
249	26	29712	18.35773865	0.9302188	0.0712586	0.0692107
249	26	29712	18.35773865	0.9302188	0.0712586	0.0692107
249	313	29712	3.812309943	0.9360442	0.0712586	0.0692107
249	253	29712	4.244773561	0.9380215	0.0712586	0.0692107
249	7	29712	51.1394148	0.9456407	0.0727519	0.0706611
249	101	29712	7.088631755	0.9632165	0.0786561	0.0763957
249	321	29712	3.717299103	0.9633455	0.0786561	0.0763957
249	29	29712	16.45866224	0.9746313	0.0825011	0.0801302
249	62	29712	9.623008162	0.9756668	0.0825011	0.0801302
249	62	29712	9.623008162	0.9756668	0.0825011	0.0801302
249	8	29712	44.74698795	0.9789822	0.0838859	0.0814752
249	110	29712	6.508652793	0.9918175	0.101588	0.0986685
249	161	29712	5.188056574	0.9924183	0.101588	0.0986685
249	417	29712	3.147669816	0.9968648	0.1174893	0.1141129
249	116	29712	6.171998338	0.9976452	0.1179536	0.1145638
249	10	29712	35.79759036	0.997851	0.1179536	0.1145638
249	10	29712	35.79759036	0.997851	0.1179536	0.1145638
249	35	29712	13.63717728	0.998249	0.1181553	0.1147597
249	170	29712	4.913394755	0.9983223	0.1181553	0.1147597
249	36	29712	13.2583668	0.9989752	0.1249403	0.1213497
249	361	29712	3.305410006	0.9992541	0.1263635	0.122732
249	11	29712	32.54326396	0.9994295	0.1263635	0.122732
249	11	29712	32.54326396	0.9994295	0.1263635	0.122732
249	11	29712	32.54326396	0.9994295	0.1263635	0.122732
249	76	29712	7.850348763	0.9995954	0.1293585	0.125641
249	38	29712	12.56055802	0.9996764	0.1293585	0.125641
249	38	29712	12.56055802	0.9996764	0.1293585	0.125641
249	39	29712	12.23849243	0.9998255	0.1370729	0.1331336
249	40	29712	11.93253012	0.9999086	0.1427518	0.1386493
249	40	29712	11.93253012	0.9999086	0.1427518	0.1386493
249	602	29712	2.576792219	0.9999637	0.1545183	0.1500777
249	132	29712	5.423877327	0.9999724	0.1562735	0.1517824
249	43	29712	11.10002802	0.9999889	0.1673474	0.1625381
249	86	29712	6.937517512	0.9999946	0.1708956	0.1659843
249	14	29712	25.5697074	0.9999948	0.1708956	0.1659843
249	14	29712	25.5697074	0.9999948	0.1708956	0.1659843
249	397	29712	3.005675093	0.9999979	0.1812824	0.1760726
249	398	29712	2.998123146	0.9999986	0.1840332	0.1787443
249	89	29712	6.703668607	0.9999989	0.184397	0.1790977

249	15	29712	23.86506024	0.9999991	0.1856484	0.1803132
249	48	29712	9.9437751	0.9999998	0.1978207	0.1921356
249	16	29712	22.37349398	0.9999999	0.1978207	0.1921356
249	16	29712	22.37349398	0.9999999	0.1978207	0.1921356
249	16	29712	22.37349398	0.99999987	0.19782068	0.19213562
249	16	29712	22.37349398	0.99999987	0.19782068	0.19213562
249	49	29712	9.740840915	0.99999993	0.20125161	0.19546795
249	94	29712	6.34709049	0.99999994	0.20125161	0.19546795
249	17	29712	21.05740609	0.99999998	0.21386013	0.20771413
249	151	29712	4.741402697	0.99999999	0.21386013	0.20771413
249	51	29712	9.358847153	0.99999999	0.21386013	0.20771413
249	97	29712	6.150788722	0.99999999	0.21386013	0.20771413
249	155	29712	4.619043918	1	0.23151463	0.22486127

Supplementary Table 3. Gene Set Enrichment Analysis (GSEA) cor
genes with an adjusted p-value < 0.05 in bone marrrow-derived macr
normalization to each mouse as a covariate. Shown are the pathways

Pathway	P.Value
HALLMARK_INTERFERON_GAMMA_RESPONSE	1.31E-05
HALLMARK_TNFA_SIGNALING_VIA_NFKB	1.31E-05
HALLMARK_INFLAMMATORY_RESPONSE	1.36E-05
HALLMARK_COMPLEMENT	1.36E-05
HALLMARK_INTERFERON_ALPHA_RESPONSE	1.44E-05
HALLMARK_IL6_JAK_STAT3_SIGNALING	3.01E-05
HALLMARK_ALLOGRAFT_REJECTION	1.49E-04
HALLMARK_XENOBIOTIC_METABOLISM	3.57E-03
HALLMARK_KRAS_SIGNALING_UP	3.57E-03
HALLMARK_UV_RESPONSE_UP	6.23E-03
HALLMARK_GLYCOLYSIS	6.85E-03
HALLMARK_NOTCH_SIGNALING	1.13E-02
HALLMARK_COAGULATION	1.33E-02
HALLMARK_TGF_BETA_SIGNALING	1.35E-02
HALLMARK_HYPOXIA	1.70E-02
HALLMARK_APOPTOSIS	2.07E-02
HALLMARK_EPITHELIAL_MESENCHYMAL_TRANSITION	2.08E-02
HALLMARK_WNT_BETA_CATENIN_SIGNALING	2.16E-02
HALLMARK_APICAL_JUNCTION	2.21E-02
HALLMARK_HEDGEHOG_SIGNALING	5.77E-02
HALLMARK_KRAS_SIGNALING_DN	6.90E-02
HALLMARK_HEME_METABOLISM	8.08E-02
HALLMARK_MYC_TARGETS_V1	8.66E-02
HALLMARK_ANGIOGENESIS	9.31E-02
HALLMARK_MYOGENESIS	9.92E-02

nducted with 1x10^5 permutations of differentially expressed
ophages after treatment with bilirubin for 48 hours and
s with a P-value of less than 0.01.

adj.P.Val	ES	NES	nMoreExtreme	size
1.44E-04	7.51E-01	2.35E+00	0	164
1.44E-04	6.72E-01	2.10E+00	0	162
1.44E-04	7.20E-01	2.19E+00	0	131
1.44E-04	6.59E-01	2.00E+00	0	129
1.44E-04	7.35E-01	2.13E+00	0	88
2.51E-04	7.22E-01	2.01E+00	1	66
1.07E-03	6.09E-01	1.85E+00	10	130
1.99E-02	5.46E-01	1.66E+00	262	130
1.99E-02	5.56E-01	1.67E+00	259	120
3.11E-02	5.39E-01	1.63E+00	454	122
3.11E-02	5.08E-01	1.58E+00	519	156
4.71E-02	-6.45E-01	-1.73E+00	471	24
4.81E-02	5.77E-01	1.63E+00	899	74
4.81E-02	-5.30E-01	-1.63E+00	486	49
5.67E-02	4.89E-01	1.51E+00	1276	146
5.81E-02	5.02E-01	1.52E+00	1511	122
5.81E-02	5.28E-01	1.55E+00	1459	96
5.81E-02	-5.82E-01	-1.62E+00	876	29
5.81E-02	5.10E-01	1.52E+00	1587	111
1.44E-01	-5.80E-01	-1.49E+00	2473	20
1.64E-01	5.32E-01	1.45E+00	4486	56
1.84E-01	4.23E-01	1.32E+00	6153	159
1.88E-01	4.04E-01	1.29E+00	6849	200
1.94E-01	-5.46E-01	-1.41E+00	3989	20
1.98E-01	-3.73E-01	-1.27E+00	3044	87

LeadingEdge
Cfb_Cl5_Fpr1_Cmklr1_Sos3_Pde4b_Ptgs2_Sod2_Irf7_Ifi44l_Usp18_Isg20_H2Eb1_Fas_Nfkbi[illegible]
Cxl3_Il1a_Cxl1_Cl5_Il1b_Cxl2_Traf1_Sos3_Pde4b_Aba1_Tlr2_Ptgs2_Nfkbie_Sod2_Relb_Ier3_
Maro_Il1a_Cl5_Fpr1_Il1b_Mmp14_Mefv_Cmklr1_Pde4b_Aba1_Tlr2_Pdpn_Irf7_Cybb_Gpr132_
Cfb_Cxl3_Cxl1_Cl5_C3_Mmp14_Cxl2_F10_Sr_Irf7_Pla2g7_F7_Hspa1a_Casp4_Col4a2_Ehd1_
Irf7_Ifi44l_Usp18_Isg20_Cd74_Isg15_Oasl1_Rtp4_Oas1g_Gbp3_Mx2_Epsti1_Gbp2_Cxl10_Ifi[illegible]
Cxl3_Cxl1_Il1b_Cxl2_Il2rg_Sos3_Tlr2_Fas_Tnf_Cxl10_Map3k8_Csf2rb2_Ebi3_Tnfrsf1b_Sos1_
Mmp9_Cl5_Il1b_Fgr_Il2rg_Tlr2_Ly75_Irf7_Fas_Tnf_Cd74_Iam1_Igsf6_Il10_Gbp2_Bl3_Cd40_[illegible]
Cfb_Sl6a12_Adh7_Arg2_Ptges_F10_Px_Nqo1_Bar1_Fas_Maoa_Mt2_Gsta3_Aox1_Spint2_Pt[illegible]
Mmp9_Cfb_Il1b_Slpi_Traf1_Cmklr1_Il2rg_Ptgs2_Cle4a1_Bir3_Cxl10_Sn1b_Mmp10_Tnfrsf1b_[illegible]
Cxl1_Mmp14_Sl6a12_Sod2_Nfkbia_Rrad_Iam1_Maoa_Cdkn1_Btg3_Fos_Junb_Aaa1b_Ctsl_E[illegible]
Van_Sd1_Px_Ier3_Isg20_Chpf_Met_Adora2b_Sl16a3_Col5a1_Ak4_Nt5e_Gp1_Egfr_Txn1_Pk[illegible]
Fzd7_Cnd1_Tf7l2_Fzd1_Fzd5_Maml2_Lfng_Prka
Mmp9_Cfb_C3_Mmp14_Htra1_F10_Ctsk_Comp_Klf7_Gda_Crip2_Mmp10_Ctsl_F9_Anxa1
Thbs1_Pmepa1_Serpine1_Id1_Smad7_Tgfbr1_Cdh1_Id3_Id2_Bmpr1a_Smad1_Skil_Furin_Bm[illegible]
Lox_Cav1_Ier3_Isg20_Cavin3_Gbe1_Adora2b_Mt2_Cdkn1_Col5a1_Ak4_Klf7_Ampd3_Sd4_Pr[illegible]
Il1a_Il1b_Cav1_Sod2_Ier3_Isg20_Fas_Tnf_Bir3_Ifitm3_Casp4_Btg3_Ml1_Anxa1_Igf2r_Irf1_Bt[illegible]
Cxl3_Cxl1_Mmp14_Cxl2_Van_Lox_Sd1_Htra1_Fas_Comp_Col5a1_Nt5e_Col4a2_Dst_Sd4_G[illegible]
Cnd2_Jag2_Noth4_Peg12_Fzd1_Pth1_Numb_Fzd8
Mmp9_Van_Traf1_Sr_Iam1_Layn_Rras_Egfr_Baiap2_Tgfbi_Vasp_Cd274_Netin3_Evl_Syk_Pt[illegible]
Nram_Amot_Nrp1_Adgrg1_Cdk5r1_Pth1_Ophn1_Tle3
Mefv_Ifi44l_Coq8a_Mx2_Tex15_Cd207_Ryr1_Knn1_Gtf35_Rsad2_Sptbn2_Ptprj_Akr1b8_Btg2_
C3_Ap5_Bam_Px_Sl7a11_Sl6a9_Sl11a2_Sna_Agpat4_Sl25a37_Blvrb_Noa4_Sl2a1_Btg2_Sl2[illegible]
Pgk1_Ldha_Pabp1_Psma7_Psma6_Cox5a_Rplp0_Psmd8_Ssbp1_Impdh2_Dut_Eef1b2_Npm1
Cnd2_Jag2_Spp1_Itgav_Pf4_Pdgfa_Nrp1_Thbd
Gpx3_Myl6b_Col1a1_Sorbs3_Tnni2_Spar_Lsp1_Dmpk_Cox7a1_Itgb5_Mef2a_Bhlhe40_Cdkn1

a_Cd74_Iam1_Isg15_Oasl1_Rtp4_Gbp3_Mx2_Epsti1_Cxl10_P2ry14_Ifitm3_Zbp1_Tnfai
_Tnf_Nfkbia_Iam1_Bir3_Z3h12a_Cxl10_Map3k8_Sl2a6_Nfkb2_Serpinb8_Tnfaip2_Bl3_(
_Met_Nfkbia_Iam1_Sl11a2_Rtp4_Il10_Cxl10_Sn1b_Adora2b_Cd82_Lpar1_Sl31a2_Cd4(

m3_Pror_Crl2_Ifi44_Ifit3_Cmpk2_Ifit2_Ddx60_Rsad2_Lgals3bp_Samd9l_Parp14_B2m_

H2Aa_Cfp_Egfr_Nlrp3_Nf4_Itgb2_Sos1_Prkb_B2m_Tapbp_Cd47_Stat1_Ifngr2

o2_Glrx_Tgfbi_Pgk1_Eno1_Cyb5a_Aldoa_Pygl_Ldha_Gys1_G6pdx_Ext1_Fam162a_Mif

dx5_Fos_Gp1_Egfr_Tnfaip3_Glrx_Tgfbi_Pgk1_Sl2a1_Btg1_Dusp1_Pnr1_Eno1_Rbpj_P
g2_Cd14_Lmna_Sqstm1_Hmgb2_Gpx1_Ptk2_Gadd45b_Gsr_Bnip3l_Casp6
o1_Tnfaip3_Tgfbi_Tpm1_Edil3_Col4a1_Anpep_Tpm4_Sntb1_Em1_Vim_Noth2

_Mrpl23_Hspe1_Snrpg_Rps5_Psma4_Hnrnpa1_Tufm_Snrpb2_Snrpd2_Lsm7_Rps6_Sr

p2_Mt2_Oas2_Casp4_Cd40_Csf2rb2_Ifi44_Ddx58_Ifit3_H2Aa_Cmpk2_Ifit2_Rnf213_Oa
Crl2_Btg3_Tnip1_Ddx58_Ehd1_Fosl1_Sd4_Fos_Ifit2_Ml1_Cebpd_Tnfaip3_Phlda1_Junb
0_Crl2_Sgms2_Ebi3_Nod2_Adgre1_Tnfrsf1b_Nlrp3_Ahr_P2rx4_Sl7a2_Tapbp_Pik3r5_B

gm1_Aldoa_Ldha_Gys1_Anxa2_Pfkl_Ext1_Fam162a_Large1_Pdk1_Mif_S100a4_Tpi1_

nrpd3_Rpl14_Snrpd1_Rpl34_Psmd7_Rad23b_Ifrd1_Ranbp1_Hdgf_Mm7_Cy1_Snrpa1_

as3_Ddx60_Tnfaip3_Rsad2_Itgb7_Xaf1_Lgals3bp_Samd9l_Mvp_Sos1_Parp14_B2m_Ta
_Rnf19b_Ninj1_Ifngr2_Klf4_Btg1_Plpp3_Irf1_Egr2_Btg2_Cebpb_Dusp1_Pnr1_Marks_S

Nap1l1_Eif4e_Ddx18_Rpl6_Rpl22_Hspd1_Ssb_G3bp1_Rps3_Vda3_Nhp2_Xpo1_Psmb

apbp_Dhx58_Cd274_Bst2_Gbp9_Stat1_Samhd1_Il18bp_Btg1_Irf1_Helz2_Plsr1_Marhf1_

3_Hdd2_Apex1_Eif2s1_Rps10_Hsp90ab1_Psmb2_Rrm1_Cbx3_Tp1_Psma2_Got2_Ct3_

_Cd20_Ptges3_Cnbp_Phb2_Cad_Rak1_Tomm70a_Erh_U2af1_Nop16_Hda2_Rpl18_De

ek_Nop56_Vda1_Kpna2_Ppia_Pa2g4_Exos7_Gspt1_Gnl3_Mm5_Cdk4_Kars_Kpnb1_Pb

p1_Nbp2_Tardbp_Srsf3_Ube2e1_Hnrnpa3_Cd45_Prdx3_Rrp9_Etf1_Ran_Srpk1_Dhx1[illegible]

5_C1qbp_Ct5_Psma1_Cul1_Ct4_Psmd3_Ap1_Eprs_Hnrnp_Eif2s2

Supplementary Table 5. Top 50 transcrip
with an adjusted p-value < 0.01 after norm

motif
hocomoco__TF65_MOUSE.H11MO.0.A
dbcorrdb__RELA__ENCSR000EAQ_1__m1
cisbp__M6511
cisbp__M4497
cisbp__M6449
cisbp__M1928
transfac_pro__M03563
transfac_pro__M07221
dbcorrdb__RELA__ENCSR000EBA_1__m1
dbcorrdb__RELA__ENCSR000EBI_1__m1
hocomoco__NFKB2_HUMAN.H11MO.0.B
transfac_pro__M00774
transfac_public__M00052
cisbp__M4468
dbcorrdb__RELA__ENCSR000EBD_1__m1
jaspar__MA0107.1
hocomoco__NFKB1_MOUSE.H11MO.0.A
dbcorrdb__RELA__ENCSR000EBM_1__m1
cisbp__M6369
cisbp__M4498
dbcorrdb__RELA__ENCSR000EAN_1__m1
dbcorrdb__RELA__ENCSR000EAW_1__m1
homer__AGGGGATTTCCC_NFkB-p65
cisbp__M4496
cisbp__M6137
dbcorrdb__RELA__ENCSR000EAI_1__m1
cisbp__M4494
cisbp__M4499
jaspar__MA0101.1
transfac_public__M00053
hocomoco__TF65_HUMAN.H11MO.0.A
transfac_public__M00054
cisbp__M3623
transfac_pro__M08891
dbcorrdb__RELA__ENCSR000DYM_1__m1
swissregulon__hs__NFKB1_REL_RELA.p2

cisbp__M4485
transfac_pro__M03557
cisbp__M4495
factorbook__NFKB1
cisbp__M1930
transfac_pro__M03545
cisbp__M4491
cisbp__M1924
cisbp__M4444
cisbp__M3091
dbcorrdb__RELA__ENCSR000EAG_1__m1
transfac_pro__M01239
transfac_public__M00208

ıtional regulators predicted to be altered with bilirubin tre
ıalization to each mouse as a covariate were used for th

TF_highConf
Rela (directAnnotation).
Rela (inferredBy_Orthology).
Rela (inferredBy_Orthology).
Rela (inferredBy_Orthology).
Rel (inferredBy_Orthology).
Nfkb1 (inferredBy_Orthology).
Rela (directAnnotation).
Nfkb1 (inferredBy_Orthology).
Rela (inferredBy_Orthology).
Rela (inferredBy_Orthology).
Nfkb2 (inferredBy_Orthology).
Nfkb1; Nfkb2; Pold2; Rel; Rela (directAnnotation).
Rela (directAnnotation).
Rela (inferredBy_Orthology).
Rela (inferredBy_Orthology).
Rela (inferredBy_Orthology).
Nfkb1 (directAnnotation).
Rela (inferredBy_Orthology).
Nfkb1 (inferredBy_Orthology).
Rela (inferredBy_Orthology).
Rela (inferredBy_Orthology).
Rela (inferredBy_Orthology).
Rela (inferredBy_Orthology).
Rela (inferredBy_Orthology).
Rela (directAnnotation).
Rela (inferredBy_Orthology).
Rela (inferredBy_Orthology).
Rela (inferredBy_Orthology).
Rel (inferredBy_Orthology).
Rel (directAnnotation).
Rela (inferredBy_Orthology).
Rel; Rela (directAnnotation). Nfkb1 (inferredBy_Orthology).
Rela (inferredBy_Orthology).
Rela (directAnnotation). Nfkb1 (inferredBy_Orthology).
Rela (inferredBy_Orthology).
Nfkb1; Rel; Rela (inferredBy_Orthology).

Rela (inferredBy_Orthology).
Nfkb1; Pold2 (directAnnotation).
Rela (inferredBy_Orthology).
Bcl3; Nfkb1 (inferredBy_Orthology).
Rela (inferredBy_Orthology).
Rel (directAnnotation).
Rela (inferredBy_Orthology).
Rel (inferredBy_Orthology).
Rela (inferredBy_Orthology).
Rel (inferredBy_Orthology).
Rela (inferredBy_Orthology).
Rela; Relb (inferredBy_Orthology).
Rel (directAnnotation). Nfkb1 (inferredBy_Orthology).

eatment in bone marrow-derived macropahges. Differentially expressed genes
ie analysis.

TF_lowConf
Nfkb1; Nfkb2; Pold2; Rel; Relb (inferredBy_MotifSimilarity). Bcl3; Hivep1 (inferredBy_MotifSimila
Nfkb1; Nfkb2; Rel; Relb (inferredBy_MotifSimilarity). Bcl3; Hivep1 (inferredBy_MotifSimilarity_n_
Nfkb1; Nfkb2; Pold2; Rel; Relb (inferredBy_MotifSimilarity). Bcl3; Hivep1 (inferredBy_MotifSimila
Nfkb1; Nfkb2; Pold2; Rel; Relb (inferredBy_MotifSimilarity). Bcl3; Hivep1 (inferredBy_MotifSimila
Nfkb1; Nfkb2; Rela; Relb (inferredBy_MotifSimilarity). Bcl3; Hivep1 (inferredBy_MotifSimilarity_n
Nfkb2; Pold2; Rel; Rela; Relb (inferredBy_MotifSimilarity). Bcl3; Hivep1 (inferredBy_MotifSimilar
Nfkb1; Nfkb2; Pold2; Rel; Relb (inferredBy_MotifSimilarity). Bcl3 (inferredBy_MotifSimilarity_n_C
Nfkb2; Pold2; Rel; Rela; Relb (inferredBy_MotifSimilarity). Bcl3; Hivep1 (inferredBy_MotifSimilar
Nfkb1; Nfkb2; Rel; Relb (inferredBy_MotifSimilarity). Bcl3; Hivep1 (inferredBy_MotifSimilarity_n_
Nfkb1; Nfkb2; Rel; Relb (inferredBy_MotifSimilarity). Bcl3; Hivep1 (inferredBy_MotifSimilarity_n_
Nfkb1; Pold2; Rel; Rela; Relb (inferredBy_MotifSimilarity). Bcl3; Cd59a; Cd59b (inferredBy_Moti
Relb (inferredBy_MotifSimilarity). Bcl3 (inferredBy_MotifSimilarity_n_Orthology).
Nfkb1; Nfkb2; Pold2; Rel; Relb (inferredBy_MotifSimilarity). Bcl3 (inferredBy_MotifSimilarity_n_C
Nfkb1; Rel; Relb (inferredBy_MotifSimilarity). Bcl3; Hivep1 (inferredBy_MotifSimilarity_n_Orthol
Nfkb1; Nfkb2; Rel; Relb (inferredBy_MotifSimilarity). Bcl3; Hivep1 (inferredBy_MotifSimilarity_n_
Nfkb1; Nfkb2; Pold2; Rel; Relb (inferredBy_MotifSimilarity). Bcl3 (inferredBy_MotifSimilarity_n_C
Nfkb2; Pold2; Rel; Rela; Relb (inferredBy_MotifSimilarity). Bcl3; Hivep1 (inferredBy_MotifSimilar
Nfkb1; Nfkb2; Pold2; Rel; Relb (inferredBy_MotifSimilarity). Bcl3; Hivep1 (inferredBy_MotifSimila
Nfkb2; Pold2; Rel; Rela; Relb (inferredBy_MotifSimilarity). Bcl3; Hivep1 (inferredBy_MotifSimilar
Nfkb1; Rel; Relb (inferredBy_MotifSimilarity). Bcl3 (inferredBy_MotifSimilarity_n_Orthology).
Nfkb1; Nfkb2; Pold2; Rel; Relb (inferredBy_MotifSimilarity). Bcl3; Hivep1 (inferredBy_MotifSimila
Nfkb1; Nfkb2; Rel; Relb (inferredBy_MotifSimilarity). Bcl3; Hivep1 (inferredBy_MotifSimilarity_n_
Nfkb1; Nfkb2; Rel; Relb (inferredBy_MotifSimilarity). Bcl3; Hivep1; Hivep2; Hivep3; Zfp831 (infer
Nfkb1; Rel; Relb (inferredBy_MotifSimilarity). Bcl3 (inferredBy_MotifSimilarity_n_Orthology).
Nfkb1; Nfkb2; Pold2; Rel; Relb (inferredBy_MotifSimilarity). Bcl3 (inferredBy_MotifSimilarity_n_C
Nfkb1; Nfkb2; Pold2; Rel; Relb (inferredBy_MotifSimilarity). Bcl3; Hivep1 (inferredBy_MotifSimila
Nfkb1; Nfkb2; Pold2; Rel; Relb (inferredBy_MotifSimilarity). Bcl3; Hivep1 (inferredBy_MotifSimila
Nfkb1; Rel; Relb (inferredBy_MotifSimilarity). Bcl3; Hivep1 (inferredBy_MotifSimilarity_n_Orthol
Nfkb1; Rela; Relb (inferredBy_MotifSimilarity). Bcl3 (inferredBy_MotifSimilarity_n_Orthology).
Nfkb1; Rela; Relb (inferredBy_MotifSimilarity). Bcl3 (inferredBy_MotifSimilarity_n_Orthology).
Nfkb1; Rel; Relb (inferredBy_MotifSimilarity). Bcl3 (inferredBy_MotifSimilarity_n_Orthology).
Nfkb2; Pold2; Relb (inferredBy_MotifSimilarity). Bcl3; Hivep1; Hivep2; Hivep3; Zfp831 (inferredB
Nfkb1; Nfkb2; Pold2; Rel; Relb (inferredBy_MotifSimilarity). Bcl3 (inferredBy_MotifSimilarity_n_C
Nfkb2; Pold2; Rel (inferredBy_MotifSimilarity).
Nfkb1; Nfkb2; Rel; Relb (inferredBy_MotifSimilarity). Bcl3; Hivep1 (inferredBy_MotifSimilarity_n_
Nfkb2; Relb (inferredBy_MotifSimilarity). Bcl3 (inferredBy_MotifSimilarity_n_Orthology).

Nfkb1; Nfkb2; Pold2; Rel; Relb (inferredBy_MotifSimilarity). Bcl3; Hivep1 (inferredBy_MotifSimila
Nfkb2; Rel; Rela (inferredBy_MotifSimilarity).
Nfkb1; Nfkb2; Rel; Relb (inferredBy_MotifSimilarity). Bcl3; Hivep1 (inferredBy_MotifSimilarity_n_
Nfkb2; Pold2; Rel; Rela; Relb (inferredBy_MotifSimilarity). Hivep1 (inferredBy_MotifSimilarity_n_
Nfkb1; Nfkb2; Pold2; Rel; Relb (inferredBy_MotifSimilarity). Bcl3 (inferredBy_MotifSimilarity_n_C
Nfkb1; Nfkb2; Pold2; Rela; Relb (inferredBy_MotifSimilarity). Bcl3 (inferredBy_MotifSimilarity_n_
Nfkb1; Nfkb2; Pold2; Rel; Relb (inferredBy_MotifSimilarity). Bcl3; Hivep1 (inferredBy_MotifSimila
Nfkb1; Rela; Relb (inferredBy_MotifSimilarity). Bcl3 (inferredBy_MotifSimilarity_n_Orthology).
Nfkb1; Nfkb2; Pold2; Rel; Relb (inferredBy_MotifSimilarity). Bcl3; Hivep1 (inferredBy_MotifSimila
Nfkb1; Rela; Relb (inferredBy_MotifSimilarity). Bcl3 (inferredBy_MotifSimilarity_n_Orthology).
Nfkb1; Nfkb2; Rel; Relb (inferredBy_MotifSimilarity). Bcl3; Hivep1 (inferredBy_MotifSimilarity_n_
Rel (inferredBy_MotifSimilarity). Bcl3; Nfkb1 (inferredBy_MotifSimilarity_n_Orthology).
Nfkb2; Pold2; Rela; Relb (inferredBy_MotifSimilarity).

NES	AUC	nEnrGenes	rankAtMax
10.7	0.113	59	604
10.2	0.108	54	623
9.7	0.104	90	1943
9.65	0.104	90	1923
9.54	0.103	49	544
9.29	0.101	150	4831
9.26	0.1	101	2381
9.21	0.0999	151	4676
9.14	0.0992	78	1866
8.91	0.0972	46	606
8.9	0.0972	86	1751
8.83	0.0966	150	4737
8.8	0.0963	57	765
8.8	0.0963	120	3505
8.76	0.096	61	1158
8.7	0.0954	58	920
8.63	0.0949	88	1849
8.61	0.0947	144	4982
8.6	0.0946	84	1637
8.59	0.0946	117	3249
8.49	0.0937	85	2143
8.47	0.0936	80	2009
8.38	0.0928	91	2210
8.22	0.0914	131	3952
8.21	0.0913	98	2384
8.18	0.0911	57	1055
8.14	0.0907	138	4145
8.11	0.0904	148	4775
8.1	0.0903	69	1389
8.02	0.0897	71	1494
8.02	0.0897	98	2337
7.97	0.0892	108	3081
7.96	0.0892	106	2986
7.93	0.0889	84	1909
7.81	0.0878	91	2492
7.75	0.0873	77	1798

7.74	0.0873	121	3527
7.73	0.0872	113	3396
7.69	0.0868	122	3636
7.55	0.0857	128	3901
7.49	0.0851	107	2897
7.38	0.0842	79	1880
7.37	0.0841	117	3187
7.36	0.084	58	996
7.27	0.0832	115	2806
7.25	0.0831	65	1290
7.23	0.0829	113	3705
6.84	0.0796	86	2094
6.52	0.0768	121	3425

EnrichedGenes
Ahnak;Bcl3;Birc3;Cav1;Ccl5;Cd82;Csf2rb2;Cxcl1;Cxcl2;Ebi3;Flrt3;Gpr141;Icam1;Ier3;Ikbke;Il1b;
Ahnak;Arg2;Bcl3;Birc3;Ccl5;Cd74;Cd82;Cdc42ep2;Csf2rb;Csf2rb2;Cxcl1;Fgr;Gas7;Gpr141;Ican
Ahnak;Aldoa;Anxa2;Arg2;B2m;Bcl3;Birc3;Cav1;Ccl5;Ccrl2;Cd74;Cd82;Cdc42ep2;Crip1;Csf2rb2
Acsl4;Ahnak;Aldoa;Anxa2;Arg2;B2m;Bcl3;Birc3;Cav1;Ccl5;Cd74;Cd82;Cdc42ep2;Csf2rb;Csf2rb
Ahnak;Arg2;Bcl3;Birc3;Ccl5;Cd82;Cxcl1;Fgr;Gpx1;Icam1;Ier3;Ikbke;Il1b;Mcoln2;Mllt6;Mmp9;N4
Abca1;Abcc1;Acsl4;Adam17;Agpat4;Ahnak;Aldoa;Ampd3;Anxa2;Arap1;Arg2;Arl6ip5;Ass1;Atox
Adam17;Ahnak;Aldoa;Anxa2;Arap1;Ass1;B2m;Bcl3;Birc3;C1rl;C3;Cav1;Ccl5;Ccrl2;Cd47;Cd74;
Abca1;Abcc1;Acsl4;Adam17;Agpat4;Ahnak;Aldoa;Ampd3;Anxa2;Arap1;Arg2;Arl6ip5;Ass1;Atox
Ahnak;Atrnl1;B2m;Bcl3;Birc3;Ccl5;Cd207;Cd74;Cd82;Cdc42ep2;Csf2rb;Csf2rb2;Cxcl1;Cxcl16;F
Ahnak;Bcl3;Birc3;Ccl5;Cd74;Cd82;Csf2rb;Csf2rb2;Fgr;Gfra2;Gpr68;Icam1;Ikbke;Il1b;Junb;Mao
Acot7;Ahnak;Anxa2;Arg2;Ass1;B2m;Bcl3;Birc3;Ccl5;Ccrl2;Cd74;Cd82;Cmklr1;Csf2rb;Csf2rb2;C
Abca1;Acsl1;Adam17;Agpat4;Ahnak;Aldoa;Ampd3;Anxa2;Anxa4;Arap1;Arg2;Arl6ip5;Atox1;Atrn
Ahnak;Anxa2;Bcl3;Birc3;Ccl5;Cd74;Cd82;Csf2rb2;Cxcl1;Cxcl2;Cxcl3;Fgr;Flrt3;Gpr141;Icam1;Ier
Acsl1;Acsl4;Adam17;Ahnak;Aif1;Aldoa;Ampd3;Anxa2;Arap1;Arl6ip5;Atox1;Atrnl1;B2m;Bcl3;Birc
Ahnak;Bcl3;Birc3;Ccl5;Cd74;Cd82;Csf2rb;Csf2rb2;Ednrb;Fgr;Gas7;Gfra2;Gpr141;Gpr68;Icam1;
Ahnak;Anxa2;Bcl3;Birc3;C1rl;Ccl5;Cd74;Cd82;Csf2rb2;Cxcl1;Cxcl2;Cxcl3;Fgr;Gpr141;Icam1;Ier
Abca1;Ahnak;Aldoa;Anxa4;Arap1;Arg2;B2m;Bcl3;Birc3;Ccl5;Cd74;Cd82;Csf2rb2;Cxcl1;Cxcl2;C
Abcc1;Acot7;Acsl1;Acsl4;Adh7;Agpat4;Ahnak;Aif1;Aldh3b1;Aldoa;Ampd3;Anxa2;Anxa4;Arap1;
Ahnak;Anxa2;Arap1;Arg2;Ass1;B2m;Bcl3;Birc3;Ccl5;Ccrl2;Cd47;Cd74;Cd82;Crip1;Csf2rb2;Cxcl
Acsl1;Acsl4;Adh7;Ahnak;Aif1;Aldoa;Ampd3;Anxa2;Arap1;Arg2;Arl6ip5;Ass1;Atox1;Atrnl1;B2m;
Agpat4;Ahnak;Ampd3;Arg2;Atox1;Atrnl1;B2m;Bcl3;Birc3;Ccl5;Cd207;Cd74;Cd82;Cdc42ep2;Csf
Agpat4;Ahnak;Ampd3;Atox1;Atrnl1;B2m;Bcl3;Birc3;Ccl5;Cd207;Cd74;Cd82;Cdc42ep2;Csf2rb;C
Acsl4;Ahnak;Anxa2;Arap1;Arg2;Bcl3;Birc3;C1rl;Cav1;Ccl5;Ccrl2;Cd74;Cd82;Cdc42ep2;Csf2rb2
Abca1;Acsl1;Acsl4;Adam17;Adh7;Agpat4;Ahnak;Aif1;Aldoa;Ampd3;Arap1;Arl6ip5;Ass1;Atox1;A
Adam17;Ahnak;Aldoa;Ampd3;Anxa2;Arl6ip5;B2m;Bcl3;Birc3;C1rl;Casp4;Cav1;Ccl5;Ccrl2;Cd74;
Ahnak;Arg2;B2m;Bcl3;Birc3;Ccl5;Cd82;Csf2rb;Csf2rb2;Cxcl1;Ednrb;Fgr;Gfra2;Gpr68;Icam1;Ier
Acsl1;Acsl4;Adh7;Agpat4;Ahnak;Aif1;Aldoa;Ampd3;Anxa2;Anxa4;Arap1;Arg2;Arl6ip5;Ass1;Atox
Abca1;Acsl1;Acsl4;Adh7;Agpat4;Ahnak;Aif1;Aldh3b1;Aldoa;Ampd3;Anxa2;Anxa4;Arap1;Arg2;A
Ahnak;Anxa2;Arg2;Bcl3;Birc3;C1rl;Ccl5;Cd82;Csf2rb;Csf2rb2;Ctsz;Cxcl1;Cxcl2;Cxcl3;Ebi3;Fgr;F
Ahnak;Anxa2;Arg2;Bcl3;Birc3;C1rl;Ccl5;Cd82;Csf2rb;Csf2rb2;Ctsz;Cxcl1;Cxcl2;Cxcl3;Ebi3;Fas;
Ahnak;Ampd3;Arap1;Arl6ip5;Atrnl1;B2m;Bcl3;Birc3;C1rl;Cav1;Ccl5;Cd74;Cd82;Crip1;Csf2rb;Cs
Acsl4;Ahnak;Aif1;Anxa2;Arap1;Ass1;B2m;Bcl3;Birc3;C1rl;Cav1;Ccl5;Ccrl2;Cd207;Cd74;Cd82;C
Acsl4;Ahnak;Aif1;Aldoa;Ampd3;Anxa2;Anxa4;Arl6ip5;Bcl3;Birc3;C1rl;Cav1;Ccl5;Cd207;Cd74;C
Ahnak;Anxa2;Arap1;Arg2;Ass1;Bcl3;Ccl5;Cd74;Cd82;Cdc42ep2;Csf2rb2;Cxcl1;Ebi3;Fas;Fgr;Ga
Agpat4;Ahnak;Ampd3;Arap1;Arg2;Atox1;B2m;Bcl3;Birc3;Ccl5;Cd207;Cd74;Cd82;Cdc42ep2;Cri
Acsl4;Ahnak;Aldoa;Anxa2;Anxa4;Arap1;B2m;Bcl3;Ccl5;Ccrl2;Cd74;Cd82;Cdc42ep2;Csf2rb2;Ct

Acsl4;Adh7;Ahnak;Aif1;Aldoa;Ampd3;Anxa2;Arap1;Arg2;Atox1;B2m;Bcl3;Birc3;C3;Cav1;Ccl5;C
Adam17;Ahnak;Aldh3b1;Aldoa;Anxa2;Anxa4;Arap1;Arg2;Ass1;Atrnl1;B2m;Bcl3;C1rl;Cav1;Ccl5;
Acsl1;Acsl4;Adh7;Ahnak;Aif1;Aldoa;Ampd3;Anxa2;Arap1;Atox1;B2m;Bcl3;Birc3;C3;Cav1;Ccl5;C
Acsl1;Acsl4;Adh7;Agpat4;Ahnak;Aif1;Aldoa;Ampd3;Anxa2;Arap1;Arg2;Ass1;Atox1;B2m;Bcl3;Bir
Agpat4;Ahnak;Aldoa;Ampd3;Anxa2;Anxa4;Arap1;Arl6ip5;B2m;Bcl3;Birc3;C1rl;Cav1;Ccl5;Cd207
Ahnak;Aldoa;Anxa2;Arg2;Ass1;B2m;Bcl3;C3;Cav1;Ccl5;Ccrl2;Cd207;Cd82;Cmklr1;Csf2rb2;Cxc
Acsl1;Acsl4;Agpat4;Ahnak;Aif1;Aldoa;Ampd3;Anxa2;Arap1;Arg2;Ass1;B2m;Bcl3;Birc3;Cav1;Ccl
Ahnak;Anxa2;Arg2;Bcl3;Birc3;C1rl;Ccl5;Cd82;Csf2rb;Csf2rb2;Ctsz;Cxcl1;Cxcl2;Cxcl3;Ebi3;Fgr;F
Acsl1;Acsl4;Adh7;Ahnak;Aif1;Aldoa;Ampd3;Arap1;Arg2;Ass1;Atox1;B2m;Bcl3;Birc3;Cav1;Ccl5;C
Ahnak;Anxa2;Arg2;Bcl3;Birc3;C1rl;Casp4;Ccl5;Cd82;Csf2rb;Csf2rb2;Ctsz;Cxcl1;Cxcl2;Cxcl3;Eb
Acsl1;Acsl4;Agpat4;Ahnak;Aif1;Ampd3;Arap1;Arg2;Atox1;Atrnl1;B2m;Bcl3;Birc3;Cav1;Ccl5;Cd2
Acsl4;Agpat4;Ahnak;Ampd3;Arap1;Arl6ip5;Atox1;Bcl3;Birc3;C1rl;Ccl5;Cd74;Cd82;Csf2rb;Csf2rb
Abca1;Abcc1;Acot7;Acsl4;Adam17;Ahnak;Ahrr;Aif1;Aldoa;Ampd3;Anxa2;Anxa4;Arap1;Ass1;Atr

Supplementary Table 6. List of primers utilized for qRT-PCR analysis.

		Primer Sequences	
**Gene**	**Species**	**Fwd Sequence 3' -> 5'**	**Rev Sequence 3' -> 5'**
Gapdh	Mouse	GACGTGCCGCCTGGAGAAAC	AGCCCAAGATGCCCTTCAGT
GAPDH	Human	CTGACTTCAACAGCGACACC	TAGCCAAATTCGTTGTCATACC
MerTK	Mouse	CCTAACCGTACCTGGTCTGAC	GGGAGGGGATTACTTTGATGTTG
MERTK	Human	CTCTGGCGTAGAGCTATCACT	AGGCTGGGTTGGTGAAAACA
Gas6	Mouse	TGCTGGCTTCCGAGTCTTC	CGGGGTCGTTCTCGAACAC
GAS6	Human	CTCGTGCAGCCTATAAACCCT	TCCTCGTGTTCACTTTCACCG
Cd274	Mouse	GCTCCAAAGGACTTGTACGTG	TGATCTGAAGGGCAGCATTTC
CD274	Human	GGACAAGCAGTGACCATCAAG	CCCAGAATTACCAAGTGAGTCCT

Harvard Primer Bank ID or Company/Cat#
N/A
N/A
7106354c3
66932917c1
9506715a1
221316737c3
11230798a1
292658763c3
