# Supplementary material for: Aggressive breast cancers secrete heme metabolites to alter macrophage immune suppression and function
Source: Commun Biol. 2026 May 9;9:977. doi: 10.1038/s42003-026-10212-0 (PMC13376764; doi:10.1038/s42003-026-10212-0)
Supplement: Supplementary file 3 — Reporting Summary [file 42003_2026_10212_MOESM3_ESM.pdf]

Corresponding author(s): Michelle M Williams

Last updated by author(s): Apr 7, 2026

Reporting Summary

Nature Portfolio wishes to improve the reproducibility of the work that we publish. This form provides structure for consistency and transparency in reporting. For further information on Nature Portfolio policies, see our Editorial Policies and the Editorial Policy Checklist.

Statistics

For all statistical analyses, confirm that the following items are present in the figure legend, table legend, main text, or Methods section.

n/a	Confirmed	
☐	☒	The exact sample size (*n*) for each experimental group/condition, given as a discrete number and unit of measurement
☐	☒	A statement on whether measurements were taken from distinct samples or whether the same sample was measured repeatedly
☐	☒	The statistical test(s) used AND whether they are one- or two-sided<br>*Only common tests should be described solely by name; describe more complex techniques in the Methods section.*
☐	☒	A description of all covariates tested
☐	☒	A description of any assumptions or corrections, such as tests of normality and adjustment for multiple comparisons
☐	☒	A full description of the statistical parameters including central tendency (e.g. means) or other basic estimates (e.g. regression coefficient) AND variation (e.g. standard deviation) or associated estimates of uncertainty (e.g. confidence intervals)
☒	☐	For null hypothesis testing, the test statistic (e.g. *F*, *t*, *r*) with confidence intervals, effect sizes, degrees of freedom and *P* value noted<br>*Give P values as exact values whenever suitable.*
☒	☐	For Bayesian analysis, information on the choice of priors and Markov chain Monte Carlo settings
☒	☐	For hierarchical and complex designs, identification of the appropriate level for tests and full reporting of outcomes
☒	☐	Estimates of effect sizes (e.g. Cohen's *d*, Pearson's *r*), indicating how they were calculated

Our web collection on statistics for biologists contains articles on many of the points above.

Software and code

Policy information about availability of computer code

Data collection	Microsoft Excel 2016-2024, IncuCyte Zoom Software (ver2018A-2024B), ImageScope x64 Software (ver12.4.0), Applied Biosystems 7500 Software (ver2.3), cBioPortal, Image Studio Software (ver12)
Data analysis	ImageScope Software (v12) was used for all histological analysis. InForm(2.6.0) and Phenoptr Softwares were used for multispectral florescence analysis. Phenoptr was run with R Studio 4.4.1. For mRNAseq, RNA-seq data were processed using the nf-core RNAseq pipeline (v3.12.0). Cutadapt (v3.4)66 as part of the trimgalore (0.6.7) package, STAR (v2.7.9a), Salmon (v1.10.1), tximport70, and the limma R package. Gene set enrichment analysis (GSEA) was performed usin the fgsea R. Over-representation analysis was performed using DAVID. The RcisTarget78 R package was used to calculate transcription regulator. Live cell imaging was assessed using the IncuCyte Software (2022B-2024B). Flow Cytometry was assessed using FlowJo (10.10.0). Data was presented with GraphPad Prism (v10.2.3 for Windows, GraphPad Software, Boston, Massachusetts USA).

For manuscripts utilizing custom algorithms or software that are central to the research but not yet described in published literature, software must be made available to editors and reviewers. We strongly encourage code deposition in a community repository (e.g. GitHub). See the Nature Portfolio guidelines for submitting code & software for further information.

Data

Policy information about availability of data

All manuscripts must include a data availability statement. This statement should provide the following information, where applicable:

- Accession codes, unique identifiers, or web links for publicly available datasets
- A description of any restrictions on data availability
- For clinical datasets or third party data, please ensure that the statement adheres to our policy

For any novel reagent or resource (e.g. 66Cl-4 HO-1 knockdown cell lines) created in the course of this project, we will follow the NIH policy on timely distribution and sharing on biomedical research resources, as published in the NIH Grants Policy Statement. As appropriate, sharing will be under the guidance of the University of Pittsburgh technology transfer operations. Raw and processed RNA-seq data was deposited in the Gene Expression Omnibus (GSE277310). All other raw data for the manuscript can be accessed on FigShare (doi.org/10.6084/m9.figshare.31014661 and doi.org/10.6084/m9.figshare.31012045).

Research involving human participants, their data, or biological material

Policy information about studies with human participants or human data. See also policy information about sex, gender (identity/presentation), and sexual orientation and race, ethnicity and racism.

Reporting on sex and gender	N/A
Reporting on race, ethnicity, or other socially relevant groupings	N/A
Population characteristics	N/A
Recruitment	N/A
Ethics oversight	N/A

Note that full information on the approval of the study protocol must also be provided in the manuscript.

Field-specific reporting

Please select the one below that is the best fit for your research. If you are not sure, read the appropriate sections before making your selection.

☒ Life sciences ☐ Behavioural & social sciences ☐ Ecological, evolutionary & environmental sciences

For a reference copy of the document with all sections, see nature.com/documents/nr-reporting-summary-flat.pdf

Life sciences study design

All studies must disclose on these points even when the disclosure is negative.

Sample size	When appropriate, tissue culture experiments are the mean of three separate experiments conducted in triplicate to decuple. For in vivo experiments, animal numbers were calculated at 80% power to the expected difference (based on published studies) at P < 0.05 (two-tailed).
Data exclusions	Mice that developed ulcers were excluded from the data analysis. For experiments with an N > 9, data points were excluded if they were two standard deviations above or below the mean.
Replication	All experiments, including animal experiments, were repeated at least once to ensure reproducibility.
Randomization	Microsoft Excel 2016-2023 was used to randomly sort animals by number and mice were then divided equally into treatment groups.
Blinding	All investigators who performed data analysis and statistics on animal experiments were blinded to study group.

Reporting for specific materials, systems and methods

We require information from authors about some types of materials, experimental systems and methods used in many studies. Here, indicate whether each material, system or method listed is relevant to your study. If you are not sure if a list item applies to your research, read the appropriate section before selecting a response.

Materials & experimental systems

n/a	Involved in the study
☐	☒ Antibodies
☐	☒ Eukaryotic cell lines
☒	☐ Palaeontology and archaeology
☐	☒ Animals and other organisms
☒	☐ Clinical data
☒	☐ Dual use research of concern
☒	☐ Plants

Methods

n/a	Involved in the study
☒	☐ ChIP-seq
☐	☒ Flow cytometry
☒	☐ MRI-based neuroimaging

Antibodies

Antibodies used

Primary - HO-1: Abcam, AB13243, 1:400; Ki67, Abcam, AB15580, 1:1600; CC-3, Cell Signaling, 9661, 1:400; CD4, Dako, 4B12, 1.4 ug/mL; FoxP3, Abcam, 236A/E7, 1:400; HO-1, Abcam, ab13243, 1:800; CD8, Dako, C8/144B, 0.4 ug/mL; Cd68, Dako, KP1, 0.12 ug/mL; PanCK, DAKO AE1/AE3, 0.18 ug/mL; F4/80, Thermofisher, MF480000, 1:12800; PD-L1, Cell Signaling, 64988, 1:50; PD-1, Cell Signaling, 84651, 1:400; CD8a, Thermofisher, clone 4SM15, 1:1600; Tox/Tox2, Cell Signaling, 73758, 1:12800; CD206, Sigma, HPA004114, 1:2000; iNOS, Cell Signaling, D6B6S, 1:1600; Arg1, Cell Signaling, D4E3M, 1:12800; COX2, Cell Signaling, D5H5, 1:300
Secondary - Anti-mu/rb Akoya ARH1001EA; Vector MP-7451 or MP-7444
Other - Spectral DAPI (Akoya, FP1490), Perkin Elmer FITC, TS000200 1:100; Perkin Elmer, Cy3.5, TS-000202, 1:500, Akoya Opals Opals 520, 540, 570, 620, 690 NEL811001KT
Flow - CD11b – Thermofisher, 364-0112-80, 0.625 uL/well; F4/80 – Biolegend, 123137, 0.375uL/well; PD-L1 – Biolegend, 124307, 0.25 uL/well; MerTK – Fisher Scientific, FAB5912U, 0.625 uL/well; Arg1 – Thermofisher, 53-3697-80, 0.3 uL/well; CD206 – Biolegend, 141727, 0.45 uL/well; iNOS – Thermofisher, 17-5920-80, 0.09uL/well; CD45 – Biolegend, 304023, 0.3 uL/well; CD14 – Biolegend, 301817, 0.75 uL/well; CD11b – Biolegend, 301345, 0.75 uL/well; HLA-DR – Biolegend, 980406, 0.75 uL/well; PD-L1 – Fisher Scientific, BDB568621, 0.75 uL/well; CD206 – Biolegend, 321105, 0.75 uL/well; Perforin – Biolegend, 154305, 0.375 uL/well; Granzyme-B – Biolegend, 515403, 0.75 uL/well; CD8a – Biolegend, 100714 or isotype control – Biolegend 400523, 0.3 [illegible]l/well, 0.3 uL/well; CD5 – Biolegend, 100625, 0.625 uL/well

Validation

We confirmed that all antibodies detected the mouse protein of interest by conducting a titer via immunohistochemistry on positive mouse tissues, as recommended by each manufacturer.

Eukaryotic cell lines

Policy information about cell lines and Sex and Gender in Research

Cell line source(s)

Mouse mammary carcinoma cells were kindly provided by Dr. Hiede Ford. RAW264.7, THP-1 and MH-S cells were kindly provided by Dr. Phillip Owens. Human BC cells were purchased from the ATCC (BT549 in 2008). Primary cells were isolated from animals or healthy female human PBMCs were purchased (Stem Cell, 200-0077).

Authentication

Human cell lines were short Tandem Repeat Fingerprinted by the University of Colorado Anschutz Medical Campus Cell Culture Services Core and had 100% match to ATCC for BT549 cells (August 2017).

Mycoplasma contamination

Cells were tested for mycoplasma contamination every three months and prior to use in in vivo experiments. The MycoAlert PLUS mycoplasma detection kit (Lonza, Cat# 75860-358) was used.

Commonly misidentified lines
(See ICLAC register)

N/A

Animals and other research organisms

Policy information about studies involving animals; ARRIVE guidelines recommended for reporting animal research, and Sex and Gender in Research

Laboratory animals

6-8 week old female (IMSR_JAX:000651; Strain #:000651)

Wild animals

N/A

Reporting on sex

Since breast cancer primarily afflicts women, only female mice were used for the proposed experiments.

Field-collected samples

N/A

Ethics oversight

All animal experiments were performed in accordance with international, national and institutional guidelines for humane research under a protocol (#00407) approved by the University of Colorado Institutional Animal Care and Use Committee (IACUC).

Note that full information on the approval of the study protocol must also be provided in the manuscript.

Plants

Seed stocks	N/A
Novel plant genotypes	N/A
Authentication	N/A

Flow Cytometry

Plots

Confirm that:

☒ The axis labels state the marker and fluorochrome used (e.g. CD4-FITC).

☒ The axis scales are clearly visible. Include numbers along axes only for bottom left plot of group (a 'group' is an analysis of identical markers).

☒ All plots are contour plots with outliers or pseudocolor plots.

☒ A numerical value for number of cells or percentage (with statistics) is provided.

Methodology

Sample preparation	Bone-marrow derived and monocyte-derived macrophages were harvested with vigorous pipetting after a 10. Minute incubation at 4-degrees in 1x PBS. Tumor associated macrophages were isolated from digested 66Cl-4 mammary tumors using a monocyte isolation kit (Miltenyi Biotech, 130-100-629).
Instrument	Penteon NovoCyte or Cytoflex LX (6-L UV)
Software	FlowJo 10.10.0 was used to analyze the data. Standard NovoCyte and Beckman Coulter software were used to collect the data.
Cell population abundance	Fluorescence minus one (FMO) controls were used to assist with the gating strategy after which the percent or MFI of each population was determined.
Gating strategy	Gating strategies for all flow panels can be seen in the supplement data.

☒ Tick this box to confirm that a figure exemplifying the gating strategy is provided in the Supplementary Information.
