# Supplementary material for: Aggressive breast cancers secrete heme metabolites to alter macrophage immune suppression and function
Source: Commun Biol. 2026 May 9;9:977. doi: 10.1038/s42003-026-10212-0 (PMC13376764; doi:10.1038/s42003-026-10212-0)
Supplement: Supplementary file 1 — Transparent Peer Review file [file 42003_2026_10212_MOESM1_ESM.pdf]

nature portfolio

Peer Review File

Aggressive breast cancers secrete heme metabolites to alter macrophage immune suppression and function

Corresponding Author: Dr Michelle Williams

This file contains all reviewer reports in order by version, followed by all author rebuttals in order by version.

Version 0:

Reviewer comments:

Reviewer #1

(Remarks to the Author)
The manuscript entitled "Aggressive breast cancers secrete heme metabolites to alter macrophage immune suppression and function" by Williams et al. addresses an important and timely question regarding the role of heme catabolism and bilirubin in shaping the tumor microenvironment (TME).
• The paper is well written, logically structured, and easy to follow.
• The authors combine clinical specimen analysis, in vivo tumor models, and in vitro functional assays to convincingly demonstrate that tumor-derived bilirubin alters macrophage function and contributes to immune suppression.
• The use of both genetic and pharmacological approaches to inhibit HO-1 increases the robustness of the findings.
Weaknesses and Points for Consideration
While the study is strong overall, there are some areas where additional clarification or experimentation could further strengthen the conclusions:
1. Tumor microenvironment characterization: Much of the immunological data relies on bulk analyses and selected markers. A more detailed dissection of the immune contexture could be obtained through flow cytometric profiling of the TME, beyond macrophages, including myeloid-derived suppressor cells (MDSCs), dendritic cells, and T cell subsets.
2. Functional heterogeneity of macrophages: Although the study assesses classical markers (CD206, PD-L1, iNOS, Arg1), macrophage biology is more complex than the M1/M2 dichotomy.
3. Direct effects on T cells: While the authors tested T cell function in co-culture, it would be informative to explore whether tumor-secreted bilirubin directly influences T cell exhaustion markers and cytokine production in vivo.
4. Mechanistic validation: The manuscript suggests NF-κB signaling as a mediator of bilirubin-induced macrophage dysfunction. This could be strengthened by rescue experiments using pharmacological or genetic inhibition of NF-κB in macrophages.
Suggested Additional Experiments
• Comprehensive flow cytometric immunophenotyping of the tumor microenvironment, with a focus on macrophage subsets, MDSCs, and T cell exhaustion markers.
• Functional validation of NF-κB involvement in bilirubin-driven macrophage reprogramming.
• Dose–response experiments to establish clinically relevant bilirubin concentrations in human tumors.
Conclusion
In summary, this is an important and well-executed study with high potential impact. Incorporating additional immunophenotyping and mechanistic validation would further enhance the strength and translational relevance of the conclusions.

Reviewer #2

(Remarks to the Author)
The authors present a study demonstrating pro-tumor effects of HO1 breast tumor expression and its metabolite bilirubin, through immune modulation leading to disease progression and increased metastatic burden. The studies are well designed, and the manuscript is well written. The materials and methods section provide sufficient detail to repeat experiments and appropriate statistical analyses were performed. I have only a few comments

1) Figure 1 shows significantly elevated HO1 as well as BV expression in ER+, HER2+, and TNBC subtypes. In panel D,

does breast tumor site refer to all subtypes or TNBC only? This should be specified. The same question applies to panel E breast cancer survival; are the survival and node status survival graphs specific to TNBC? If these graphs reflect survival among all subtypes, is HO1 more strongly predictive in TNBC? Also, the n values should be shown in the graphs.
2) Does Supp Fig 1C reflect all subtypes or TNBC?
3) Bilirubin, the product of heme catabolism by HO1, dynamically impacted both macrophage physiology and tumor-associated functions including abated engulfment of dead tumor cells and increased T cell suppression. Toward this end, the authors showed elevated CD206 by bilirubin, which the authors attribute to a pro-metastatic phenotype. Along this line, did the authors look at matrix remodeling through increased EGF and downstream signaling? CD206 can also be associated with antigen presentation; given that macrophages are antigen presenting cells, have the authors looked at modulation of antigen presentation and its effect on adaptive immunity in their model?
4) The authors show no changes in free iron in the HO1 ko mouse; did they look at tumor expression of other heme proteins like iNOS and COX2, which also require NFkB and promote immune suppression?
5) The authors used multiplex fluorescence imaging; did it reveal any spatial information regarding the effect of HO1 tumor expression and bilirubin associated with stroma restriction of CD8+ T cells or their ability to infiltrate into the tumor?

Reviewer #3

(Remarks to the Author)
In this study, the authors addressed a current topic of great interest: the role of HO-1 and its metabolites in the tumor microenvironment and how the metabolites of HO-1 are involved in maintaining an immunosuppressive and consequently pro-tumor microenvironment. Numerous studies in literature are now addressing this topic.

The authors using a public dataset portal evaluate the expression of enzymes involved in heme catabolism in different subtypes of breast cancer especially in TNBC highlighting how its high expression is correlate with lymphnode and distan metastasis and with a decrease in overall survival.

To understand the role of HMOX1 on TME they use a Cybersort analysis for predicting immune cell abundance in TNBC samples showing that M2 macrophages are the most abundant cells in tumors with high HO-1 expression while no difference was found between M1, CD8+, and Tregs.

By using an orthotopic model of breast cancer that metastasizes to the lung, the authors using a well known inhibitor of HMOX1 activity, to demonstrate the role of HMOX1 in tumor progression. Moreover, they developped an orthotopic model with HMOX1 knockdown ashowing delayed tumor growth and smaller lung metastases.

The authors argue that breast tumors have a microenvironment rich in bilirubin and, to verify whether bilirubin can alter immune functions, they treat BMDM with exogenous bilirubin and profile cells with the NOVASEQ 600 platform, highlighting that treatment with bilirubin significantly upregulates genes encoding pro-inflammatory cytokines and pro-metastatic factors, while downregulating genes that encode physiological functions such as phagocytosis and efferocytosis. With regards to efferocytosis in cell culture (Raw264.7, THP-1 and BMDM) they showed that bilirubin treatment affects efferocytosis function. Also proved by culturing Raw264.7 cells with conditioned media from66CL-4 cells treated with SnMP (Fig 4 F). Furthermore, they identified NFkB as the transcription factor primarily involved after bilirubin treatment, and confirmed this on Raw264.7 cells.
Main adress:
Fig. 4: This set of experiments lacks treatment of Raw 264.7 with bilirubin alone to see the effect that bilirubin alone has on the expression of MerkT and Gas6. In addition, the control medium conditioned with DMSO alone must be added.

Fig. 5 and related supplementary figures:
To test the effects of bilirubin on macrophage phenotype, the authors treated BMDMs with increasing concentrations of bilirubin. Bilirubin at the highest concentration increases the number of live macrophages (Supplementary Figure 9B): did the authors want to indicate the toxicity of bilirubin with these data? It is unclear what the authors mean by this result.

Furthermore, bilirubin itself does not affect the number of Arg1-positive (M2) cells, but it increases the number of iNOS+ cells. Since the authors hypothesized that HMOX1 generates an immunosuppressive microenvironment through its byproducts, focusing in particular on bilirubin, I would have expected BR-treated BMDMs to express an M2 polarization marker (Supplementary Figure 9 C and D). In contrast, BMDMs exposed to bilirubin express a marker typical of M1 polarization (iNOS). To better explain this result and in this regard, in the results section (page 12, line 262), the sentence should be revised: bilirubin generates an immunosuppressive microenvironment and possibly a reprogramming of macrophages but not an inflammatory programming of macrophages. Please revise this sentence as well.
In an immunosuppressive tumor microenvironment, efferocytosis is decreased. How can the increase in MerTK expression be explained? (supp Figure 9E).

In Figures 5A and B, the statistics for treatments with bilirubin versus IL4/13 are missing; otherwise, the graphs should be separated. Furthermore, line 302 on page 14, Fig. 5G does not correspond to the figure where panel 5G is missing but panel 5E is present. In this panel, CD8 control is missing to make comparisons. Line 307-308, rewrite the sentence because it is not clear.

In Supp Figure 10 the y-axis of the graph shows CD14 positive, so correct the graph title with PBMCs number. Furthermore, HLA-DR, a marker used in combination with other markers to distinguish between different types of macrophages, is

expressed by M1. In this case, bilirubin, which should create an immunosuppressive microenvironment, promotes the expression of an M1 marker. How do you explain this?

Fig. 6 and related supplementary figures:
The authors use software to evaluate survival curves (KMplotter) and state that “during treatment, they predicted a decrease in progression-free survival and overall survival in several types of cancer” (lines 311-315, page 14). This is true; in fact, HMOX1 expression is associated with decreased survival. My question is: which types of cancer were evaluated? They are not mentioned in the results, in the figure, or in the legend. However, this sentence and this result would perhaps be more appropriate at the beginning of the article, when discussing HMOX1 in different types of breast cancer and where survival curves are already present.

The sentence referring to OB24 restoring sensitivity to immunotherapy is perhaps superfluous, especially since experiments with SnMP and shRNA are then continued to prove this. Perhaps it would be better to rewrite it.

Generally, in an immunosuppressive TME, in addition to M2 macrophages, Tregs are also present. The data presented indicate that Tregs increase in the experimental condition of HMOX1 knockdown (supp fig 12). How can this be explained?

The work deals with a very recent and important topic. However, there are many points that need further investigation. The authors focused on the role of HMOX1 and its metabolites and in particular bilirubin expressed at the level of tumor cells and what effects its products have on TME cells, but it is also interesting and important understand the role of HMOX1 when expressed by immune system cells such as macrophages and T cells (Tregs and CD8+).
The results are numerous but are not listed in an orderly manner, which creates confusion during read.
Furthermore, probably due to the impressive number of results, the graphs contain some errors and are not stylistically consistent (minor points but important to revise). However, some control experiments are missing. The discussion is very general, also in the conclusion, and lacks some recent evidence on the subject.

Version 1:

Reviewer comments:

Reviewer #1

(Remarks to the Author)

Reviewer #2

(Remarks to the Author)
Accept
The authors have addressed all comments

Reviewer #3

(Remarks to the Author)
The authors have taken all comments and suggestions into consideration, revising the manuscript point by point. I believe the paper is now ready for publication.

Open Access This Peer Review File is licensed under a Creative Commons Attribution 4.0 International License, which permits use, sharing, adaptation, distribution and reproduction in any medium or format, as long as you give appropriate credit to the original author(s) and the source, provide a link to the Creative Commons license, and indicate if changes were made.
In cases where reviewers are anonymous, credit should be given to 'Anonymous Referee' and the source.
The images or other third party material in this Peer Review File are included in the article's Creative Commons license, unless indicated otherwise in a credit line to the material. If material is not included in the article's Creative Commons license and your intended use is not permitted by statutory regulation or exceeds the permitted use, you will need to obtain permission directly from the copyright holder.
To view a copy of this license, visit https://creativecommons.org/licenses/by/4.0/

Point-by-Point Rebuttal COMMSBIO-25-7981-T

Reviewer #1 (Remarks to the Author):

The manuscript entitled “Aggressive breast cancers secrete heme metabolites to alter macrophage immune suppression and function” by Williams et al. addresses an important and timely question regarding the role of heme catabolism and bilirubin in shaping the tumor microenvironment (TME).

- The paper is well written, logically structured, and easy to follow.
- The authors combine clinical specimen analysis, in vivo tumor models, and in vitro functional assays to convincingly demonstrate that tumor-derived bilirubin alters macrophage function and contributes to immune suppression.
- The use of both genetic and pharmacological approaches to inhibit HO-1 increases the robustness of the findings.

Weaknesses and Points for Consideration

While the study is strong overall, there are some areas where additional clarification or experimentation could further strengthen the conclusions:

1. Tumor microenvironment characterization: Much of the immunological data relies on bulk analyses and selected markers. A more detailed dissection of the immune contexture could be obtained through flow cytometric profiling of the TME, beyond macrophages, including myeloid-derived suppressor cells (MDSCs), dendritic cells, and T cell subsets.

> We thank the reviewer for this comment and agree that there are likely broad impacts of HO-1 inhibition on the tumor microenvironment. For this manuscript, we took a targeted approach and focused on delineating the effect of tumor-HO-1 on macrophages as they are the most prevalent immune cell type in most solid tumors including breast (PMID: 30361552). Our analysis showed that macrophage density was significantly increased in breast tumors with elevated tumor-HO-1 (Figure 2D-E). Our ongoing work is using a broader approach by implementing spatial sequencing to further dissect changes in heterogeneous macrophage populations, as well as other immune cell types including T cells, dendritic cells, and MDSCs. Based on the data presented here, we predict we will see changes in several suppressive immune cell populations such as Tregs and exhausted T cells upon pharmacologic and genetic HO-1 inhibition as discussed now:
>
> > Lines 419-421: “These works highlight the need to expand our findings with additional studies that assess the impact of HO-1 inhibition on immune cell populations other than macrophages including myeloid-derived suppressor cells, dendritic cells, and T cells.”

2. Functional heterogeneity of macrophages: Although the study assesses classical markers (CD206, PD-L1, iNOS, Arg1), macrophage biology is more complex than the M1/M2 dichotomy.

> We agree wholeheartedly with the reviewer and include discussion of this point in the revised text. This complexity is also what drove us to characterize macrophages beyond common M1/M2 markers in Figure 4 and 5 by observing their function (efferocytosis and immune suppression).
>
> > Lines 426-434: “The combination of *Hmox1* knockdown and checkpoint inhibition dramatically impacted expression of PD-L1 and CD206. […] Conversely, expression of macrophage polarization markers was not significantly altered in any treatment group. These findings are consistent with the idea that macrophages are more heterogenous and diverse than the binary M1/M2 macrophage polarization model and led us to focus on testing the effects of bilirubin on

macrophage function. Importantly, this is the first report to demonstrate that bilirubin blocks macrophage dead cell engulfment."

3. Direct effects on T cells: While the authors tested T cell function in co-culture, it would be informative to explore whether tumor-secreted bilirubin directly influences T cell exhaustion markers and cytokine production in vivo.

We thank the reviewer for this comment and would like to highlight the data presented in Figure 6A-B. Using a multispectral fluorescence panel, we demonstrated that mammary tumors harboring *Hmox1* knockdown had a significant increase in $CD8^+$ T cells and exhausted T cells ($CD8^+PD\text{-}1^+TIM3^+TOX^+$) compared to control tumors. These findings suggest that upon tumor-HO-1 inhibition there was an anti-tumor T cell response, justifying our subsequent experiments combining *Hmox1* knockdown with checkpoint inhibition.

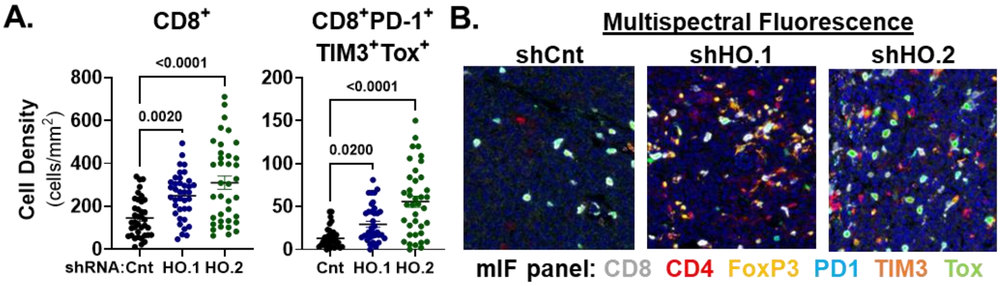

Figure 6 A-B. *Suppressive T cells were observed in 66Cl-4 shCnt, shHO.1, and shHO.2 mammary tumors using a custom multispectral fluorescence panel (CD8/grey, CD4/red, FoxP3/yellow, PD-1/cyan, TIM3/orange, TOX1/2/green). Shown is the quantification for the density of $CD8^+$ cells and exhausted $PD\text{-}1^+TIM3^+Tox^+$ cytotoxic T cells (A) and representative images (B, mean ± SEM, one-way ANOVA with Tukey's multiple comparison test).*

4. Mechanistic validation: The manuscript suggests NF-κB signaling as a mediator of bilirubin-induced macrophage dysfunction. This could be strengthened by rescue experiments using pharmacological or genetic inhibition of NF-κB in macrophages.

This is an excellent point. Ongoing work in the lab is focused on further interrogating the molecular mechanism by which bilirubin induces NFκB signaling and macrophage-mediated tumor progression. These studies are being conducted with an increasing concentration of bilirubin (0-150 µM) to further assess dose dependent responses. These concentrations were selected based on our analysis of tumor interstitial fluid conducted in Figure 4B that revealed bilirubin levels vary from ~2 to ~130 µM in mammary tumors. Importantly, this range includes levels of serum bilirubin detected in breast cancer patients that varied from ~20 µM to ~50 µM (PMID: 12865918).

As we develop these studies, we tested if the NFκB inhibitor BAY 11-7082 would reverse the impact of bilirubin on macrophage efferocytosis (Supp Fig 7E). Our new data suggests that bilirubin may alter macrophage function through this signaling pathway:

Lines 246-250: "Next, we assessed the impact of bilirubin-induced NFκB signaling on efferocytosis conducting a similar assay in the presence and absence of an NFκB inhibitor (Supp Fig 7E). Blockade of NFκB signaling rescued efferocytosis when used in combination with physiologic bilirubin concentrations."

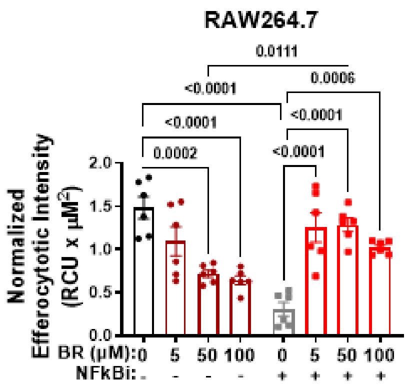

Supp Figure 7E. *RAW264.7 macrophages were cultured with pHrodo dyed dead tumor cells (66Cl-4) and treated with 0-100 µM BR and/or the NFκBi inhibitor (BAY 11-7082, 1.5 µM). Efferocytic intensity (red signal per µM²) was observed at 24 hours using the IncuCyte Zoom imaging system (n = 6, mean ± SEM, one-way ANOVA with Tukey's multiplex comparison test).*

We also added to the discussion the following comments to emphasize that we predict that further studies will reveal that bilirubin alters macrophage function and NFκB signaling in a dose dependent manner:

> Lines 446-451: "Although we observed that macrophage efferocytosis was altered by a wide range of bilirubin concentrations (0-100 µM), dose dependency needs to be assessed in future studies that will first evaluate levels of bilirubin in human TIF to provide a clinically relevant intratumoral concentration. After which dose response to physiological bilirubin concentrations should be assessed to evaluate macrophage survival, efferocytosis, and immune suppression."

Suggested Additional Experiments – Addressed above

- Comprehensive flow cytometric immunophenotyping of the tumor microenvironment, with a focus on macrophage subsets, MDSCs, and T cell exhaustion markers.
- Functional validation of NF-κB involvement in bilirubin-driven macrophage reprogramming.
- Dose–response experiments to establish clinically relevant bilirubin concentrations in human tumors.

Conclusion

In summary, this is an important and well-executed study with high potential impact. Incorporating additional immunophenotyping and mechanistic validation would further enhance the strength and translational relevance of the conclusions.

Reviewer #2 (Remarks to the Author):

The authors present a study demonstrating pro-tumor effects of HO1 breast tumor expression and its metabolite bilirubin, through immune modulation leading to disease progression and increased metastatic burden. The studies are well designed, and the manuscript is well written. The materials and methods section provide sufficient detail to repeat experiments and appropriate statistical analyses were performed. I have only a few comments

1) Figure 1 shows significantly elevated HO1 as well as BV expression in ER+, HER2+, and TNBC subtypes. In panel D, does breast tumor site refer to all subtypes or TNBC only? This should be specified. The same question applies to panel E breast cancer survival; are the survival and node status survival graphs specific to TNBC? If these graphs reflect survival among all subtypes, is HO1 more strongly predictive in TNBC? Also, the n values should be shown in the graphs.

2) Does Supp Fig 1C reflect all subtypes or TNBC?

We thank the reviewer for requesting additional details for Figure 1 and Supplemental Figure 1. The language in the manuscript has been edited in the second paragraph of the results section to clarify that the data shown in Figure 1D-E and Supplemental Figure 1C includes all breast cancer subtypes.

We also included the n for all groups presented in Figure 1D-E and Supp Figure 1C (see example below). There was a small n for HER2+ (n=20) and TNBC (n=25) specimens included in the TMA and an overall low n in KM Plotter breast cancer protein cohort (n = 65). For this reason, we did not further stratify by subtype.

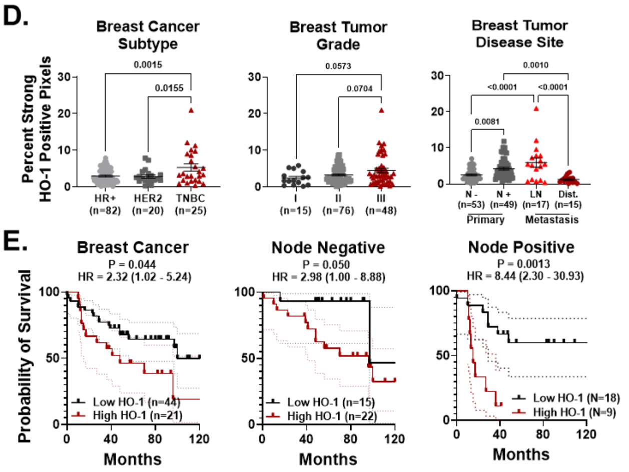

Figure 1D-E. D. *HO-1 levels were assessed in a tissue microarray via IHC. Shown is the average percent medium (2+) and strong (3+) positive pixels determined with ImageScope software for 1-4 tissue cores per specimen for primary node negative, primary node positive, local lymph node metastases, and distant metastases (mean ± SEM, one-way ANOVA with Tukey's multiple comparison test). Examples of HO-1 positive cells are denoted with arrows.* ***E.*** *Overall survival of breast cancer patients was assessed using Kaplan-Meier Plotter and the best cutoff for HO-1 protein expression (p-value log-rank/Mantel-Cox test and hazard ratio/HR Mantel-Haenszel test). The same analysis was completed after patients were stratified by lymph node involvement.*

Although we cannot stratify the data in Figure 1E by subtype due to small cohort size, using *HMOX1* expression from mRNAseq data in the KM Plotter dataset, we show that gene expression predicts a

moderate but significant decrease in overall survival in both ER$^+$ and TNBC patients. Similar results were seen in the METABRIC and TCGA datasets.

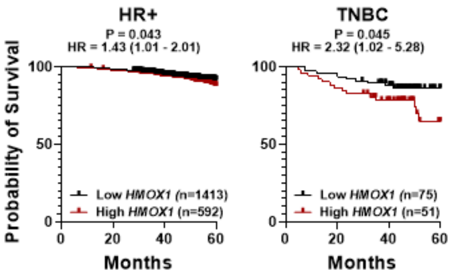

Overall survival of ER$^+$ and TNBC breast cancer patients assessed using Kaplan-Meier Plotter and the best cutoff for the mean of HMOX1 *mRNA expression (p-value log-rank/Mantel-Cox test and hazard ratio/HR Mantel-Haenszel test).*

3) Bilirubin, the product of heme catabolism by HO1, dynamically impacted both macrophage physiology and tumor-associated functions including abated engulfment of dead tumor cells and increased T cell suppression. Toward this end, the authors showed elevated CD206 by bilirubin, which the authors attribute to a pro-metastatic phenotype. Along this line, did the authors look at matrix remodeling through increased EGF and downstream signaling? CD206 can also be associated with antigen presentation; given that macrophages are antigen presenting cells, have the authors looked at modulation of antigen presentation and its effect on adaptive immunity in their model?

We appreciate these comments from the reviewer as they led us to explore a potential mechanism used by bilirubin-induced CD206$^+$ macrophages to support tumor progression. In new data, we show that HO-1 inhibition altered ECM composition via trichrome staining (Supp Figure 15A&C). We also attempted to assess EGF concentrations in our macrophage models but could not detect EGF at baseline or with bilirubin stimulation via ELISA. Our new results suggest that tumor-HO-1 and bilirubin may support a pro-tumor, ECM remodeling CD206$^+$ macrophage population, although the mechanism supporting this phenotype requires further exploration.

> Lines 369-372: "An important pro-metastatic function of CD206$^+$ macrophages is extracellular matrix (ECM) remodeling.[50] Analysis of mammary tumors using Masson's Trichrome staining revealed a significant decrease in collagen deposition within mammary tumors harboring *Hmox1* knockdown when compared to controls (Supp Fig 15A)."

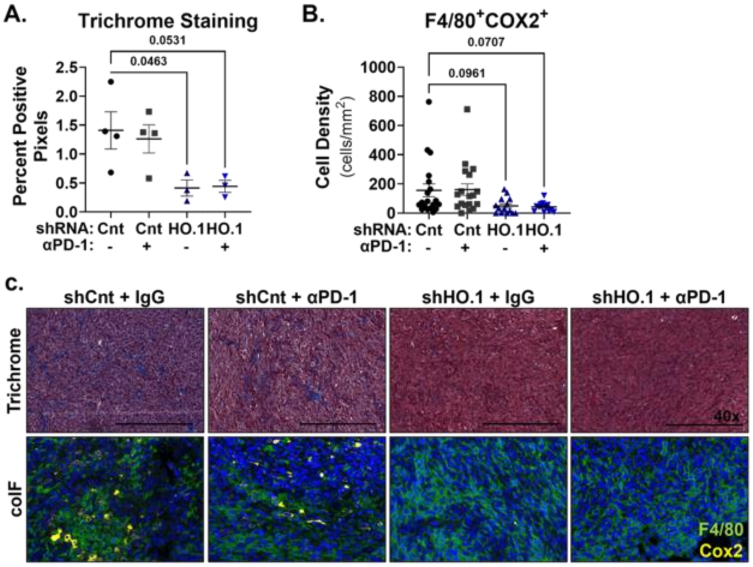

Supp Figure 15. HO-1 inhibition alters tumor extracellular matrix composition. ***A.*** *Masson's trichrome staining was conducted in tumors from Figure 6C. The percent blue positive pixels (collagen) were quantified and shown (mean ± SEM, one-way ANOVA with Dunnett's multiple comparison test).* ***B.*** *Co-immunofluorescence for F4/80 and Cox2 was conducted in the same tissues. Shown is the density of dual positive cells (mean ± SEM, one-way ANOVA with Dunnett's multiple comparison test).* ***C.*** *Representative images for trichrome staining and coIF are shown (scale bar = 200 microns, green = F4/80, yellow = COX2).*

We also assessed the expression of MHC-II in macrophages from mammary tumors with and without *Hmox1* knockdown. Although there was a trend for increased density of MHC-II$^+$F4/80$^+$ cells in HO-1 depleted tumors when compared to controls, it was not significant. Together these results suggest that CD206$^+$ macrophages in the context of tumor-HO-1 modulation have an altered matrix remodeling phenotype more so than an antigen presentation phenotype.

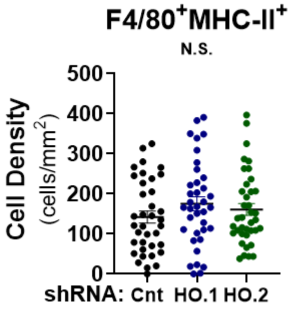

MHC-II expression was observed in F4/80$^+$ macrophages in 66Cl-4 shCnt, shHO.1, and shHO.2 mammary tumors using co-immunofluorescence. Shown is the quantification for the density of F4/80$^+$MHC-II$^+$ cells (mean ± SEM, one-way ANOVA with Tukey's multiple comparison test).

4) The authors show no changes in free iron in the HO1 ko mouse; did they look at tumor expression of other heme proteins like iNOS and COX2, which also require NFkB and promote immune suppression?

In new data, we assessed the expression of Cox2 in F4/80$^+$ macrophages in *Hmox1* knockdown tumors treated with and without checkpoint blockade via coIF. This analysis revealed a trend towards a decrease in Cox2$^+$F4/80$^+$ cells in HO-1 depleted tumors (data can be seen above under comment #3).

<u>Lines 373-375:</u> "The density of Cox2$^+$ macrophages, which can be induced by ECM[51] and support breast tumor progression[52,53], trended towards a decrease in HO-1 depleted mammary tumors compared to controls (Supp Fig 15B-C)."

We observed expression of iNOS in bilirubin treated bone marrow derived macrophages and saw a dose dependent increase in expression (Supp Figure 9D). This finding further strengthens our mRNAseq analysis that revealed NFκB is transcriptionally active upon bilirubin treatment:

<u>Lines 276-278</u>: "These findings are consistent with our transcriptional data that revealed that bilirubin induces an NFκB transcriptional program in macrophages, which has been shown by others to support macrophage survival and iNOS expression.[48,49]"

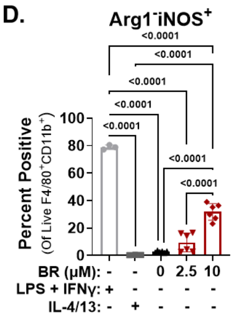

Supp Figure 9D. *Bone marrow-derived macrophages treated with controls for pro-tumor (20 ng/mL IL-4 and IL-13) and anti-tumor (20 ng/mL IFNγ and 100 ng/mL LPS) polarization or 0-10 μM bilirubin for 48 hours. Shown is the number percent Arg1⁻iNOS⁺ positive live macrophages (n = 3-6 per group, mean $\pm$ SEM, one-way ANOVA with Tukey's multiplex comparison test).*

5) The authors used multiplex fluorescence imaging; did it reveal any spatial information regarding the effect of HO1 tumor expression and bilirubin associated with stroma restriction of CD8+ T cells or their ability to infiltrate into the tumor?

We acknowledge the importance of spatial localization of T cell populations mentioned by the reviewer. However, we did not see any alterations in spatial location of T cells in our data. Instead, T cells were evenly distributed throughout tumors in all treatment groups. To demonstrate this, we added lower magnification images of our multiplex fluorescence to Supp Figure 12B and edited the text:

> Lines 335-338: "Interestingly, $CD8^+$ T cells and exhausted T cells were not spatially restricted to the stroma or periphery in any of the treatment groups, but they were significantly increased in *Hmox1* knockdown tumors when compared to shCnt tumors (Fig 6A-B, Supp Fig 12B)."

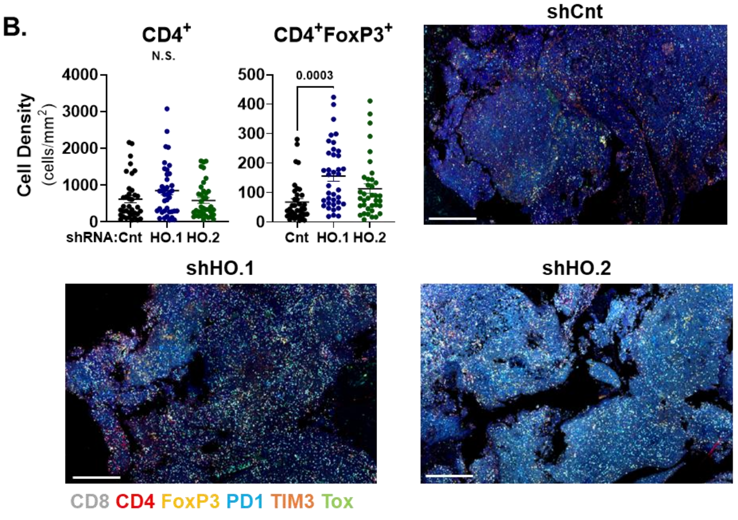

Supp Figure 12B. *Suppressive T cells were observed in 66Cl-4 shCnt, shHO.1, and shHO.2 mammary tumors using a custom multispectral fluorescence panel (T cell: CD8/grey, CD4/red, FoxP3/yellow, PD-1/cyan, TIM3/orange, TOX1/2/green). Shown is the quantification for the density of regulatory T cells (for all mean $\pm$ SEM, one-way ANOVA with Tukey's multiple comparison test). Zoomed out representative images are also shown, scale bar = 1 mm.*

Reviewer #3 (Remarks to the Author):

In this study, the authors addressed a current topic of great interest: the role of HO-1 and its metabolites in the tumor microenvironment and how the metabolites of HO-1 are involved in maintaining an immunosuppressive and consequently pro-tumor microenvironment. Numerous studies in literature are now addressing this topic.

The authors using a public dataset portal evaluate the expression of enzymes involved in heme catabolism in different subtypes of breast cancer especially in TNBC highlighting how its high expression is correlate with lymphnode and distan metastasis and with a decrease in overall survival.

To understand the role of HMOX1 on TME they use a Cybersort analysis for predicting immune cell abundance in TNBC samples showing that M2 macrophages are the most abundant cells in tumors with high HO-1 expression while no difference was found between M1, CD8+, and Tregs.

By using an orthotopic model of breast cancer that metastasizes to the lung, the authors using a well-known inhibitor of HMOX1 activity, to demonstrate the role of HMOX1 in tumor progression. Moreover, they developped an orthotopic model with HMOX1 knockdown ashowing delayed tumor growth and smaller lung metastases.

The authors argue that breast tumors have a microenvironment rich in bilirubin and, to verify whether bilirubin can alter immune functions, they treat BMDM with exogenous bilirubin and profile cells with the NOVASEQ 600 platform, highlighting that treatment with bilirubin significantly upregulates genes encoding pro-inflammatory cytokines and pro-metastatic factors, while downregulating genes that encode physiological functions such as phagocytosis and efferocytosis. With regards to efferocytosis in cell culture (Raw264.7, THP-1 and BMDM) they showed that bilirubin treatment affects efferocytosis function. Also proved by culturing Raw264.7 cells with conditioned media from66CL-4 cells treated with SnMP (Fig 4 F). Furthermore, they identified NFkB as the transcription factor primarily involved after bilirubin treatment and confirmed this on Raw264.7 cells.

Main adress:
Fig. 4: This set of experiments lacks treatment of Raw 264.7 with bilirubin alone to see the effect that bilirubin alone has on the expression of MerkT and Gas6. In addition, the control medium conditioned with DMSO alone must be added.

> We thank the reviewer for this comment and clarified the figure to show that the DMSO conditioned medium control was included in our original analysis (black bars in Figure 4F). We'd also like to highlight the data in the supplement where various macrophages models were treated with bilirubin alone at 2.5 and 10 µM concentrations. A modest but significant decrease in *MerTK* or *Gas6* was seen in each model the 2.5 µM concentration (Supp Figure 7D).

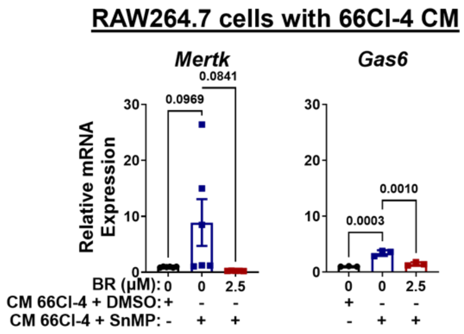

Figure 4F. *Efferocytosis genes (MertK and Gas6) were observed via qRT-PCR in RAW264.7 cells after 48 hours treatment with conditioned medium (CM) from 66Cl-4 cells with and without HO-1 inhibition (10 µM SnMP) and 2.5 µM bilirubin treatment (n = 3-6, mean ± SD, one-way ANOVA with Tukey's multiple comparison test).*

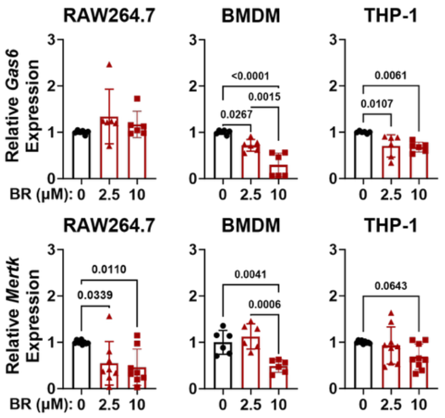

Supp Figure 7D. *RAW264.7, BMDM, or THP-1 cells were treated with 0-10 μM bilirubin for 48 hours and qRT-PCR was used to assess mRNA expression (n = 6-9 per group, mean ± SD, one-way ANOVA with Tukey's multiplex comparison test).*

Fig. 5 and related supplementary figures:
To test the effects of bilirubin on macrophage phenotype, the authors treated BMDMs with increasing concentrations of bilirubin. Bilirubin at the highest concentration increases the number of live macrophages (Supplementary Figure 9B): did the authors want to indicate the toxicity of bilirubin with these data? It is unclear what the authors mean by this result.

Thank you for letting us know this finding was unclear. We discussed this data because bilirubin at very high concentrations is commonly thought to be toxic; for example, in neurons from newborns with jaundice. Our result showing that bilirubin treatment increased the number of live macrophages was unexpected. However, it supports our mRNAseq data demonstrating that bilirubin induced NFκB signaling since this pathway can support macrophage survival. We clarified these ideas in the manuscript:

> Lines 276-278: "These findings are consistent with our transcriptional data that revealed that bilirubin induces an NFκB transcriptional program in macrophages, which has been shown by others to support macrophage survival and iNOS expression.[48,49]"

Further, we call for additional studies that go beyond these findings to explore the impact of a wider range of bilirubin doses on these macrophage phenotypes. It is possible that these studies will reveal toxicity at very high doses of bilirubin.

> Lines 446-451: "Although we observed that macrophage efferocytosis was altered by a wide range of bilirubin concentrations (0-100 μM), dose dependency needs to be assessed in future studies that will first evaluate levels of bilirubin in human TIF to provide a clinically relevant intratumoral concentration. After which, dose response to physiological bilirubin concentrations should be assessed to evaluate macrophage survival, efferocytosis, and immune suppression."

Furthermore, bilirubin itself does not affect the number of Arg1-positive (M2) cells, but it increases the number of iNOS+ cells. Since the authors hypothesized that HMOX1 generates an immunosuppressive microenvironment through its byproducts, focusing in particular on bilirubin, I would have expected BR-treated BMDMs to express an M2 polarization marker (Supplementary Figure 9 C and D). In contrast, BMDMs exposed to bilirubin express a marker typical of M1 polarization (iNOS). To better explain this result and in this regard, in the results section (page 12, line 262), the sentence should be revised: bilirubin generates an immunosuppressive microenvironment and possibly a reprogramming of macrophages but not an inflammatory programming of macrophages. Please revise this sentence as well.

We agree with the reviewer that this result was unexpected. However, it supports our finding that bilirubin induces NFκB signaling in macrophages. We have clarified the language in line 262 (now lines 276-278):

> Lines 276-278: "These findings are consistent with our transcriptional data that revealed that bilirubin induces an NFκB transcriptional program in macrophages, which has been shown by others to support macrophage survival and iNOS expression.[48,49]"

We would also like to highlight our discussion of M1 versus M2 macrophages:

> Lines 426-434: "The combination of *Hmox1* knockdown and checkpoint inhibition dramatically impacted expression of PD-L1 and CD206. […] Conversely, expression of macrophage polarization markers was not significantly altered in any treatment group. These findings are consistent with the idea that macrophages are more heterogenous and diverse than the binary M1/M2 macrophage polarization model and led us to focus on testing the effects of bilirubin on macrophage function. Importantly, this is the first report to demonstrate that bilirubin blocks macrophage dead cell engulfment."

In an immunosuppressive tumor microenvironment, efferocytosis is decreased. How can the increase in MerTK expression be explained? (supp Figure 9E).

We thank the reviewer for pointing out this contradiction. Upon re-analysis we decided to include additional data demonstrating that while the population of MerTK$^+$ macrophages increases by ~3-fold upon bilirubin treatment, it only comprises about 4% of live macrophages (Supp Figure 9E). It is possible that even though the MerTK MFI is elevated, this small population of macrophages is not sufficient to alter overall efferocytosis. In current work, we are exploring this hypothesis using flow cytometry to profile MerTK and efferocytosis in the same cells. We are also exploring expression of additional TAM receptors such as Tryo3 and Axl, as well as other pathways that regulate efferocytosis that could be impacted by bilirubin.

> Lines 274-275: "There was a modest but significant increase in the percent MerTK positive BMDM and the mean fluorescence intensity (MFI) of MerTK in BMDM treated with bilirubin (Supp Fig 9E)."

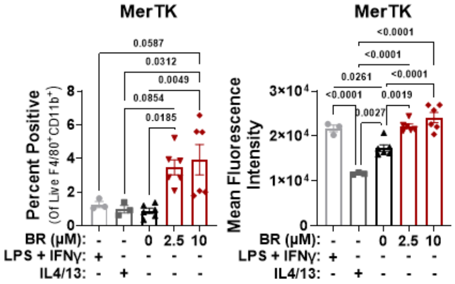

Supp Figure 9E. *Bone marrow-derived macrophages treated with controls for pro-tumor (20 ng/mL IL-4 and IL-13) and anti-tumor (20 ng/mL IFNγ and 100 ng/mL LPS) polarization or 0-10 μM bilirubin for 48 hours. Shown is the percent positive and mean fluorescence intensity of MerTK in live macrophages (n = 3-6 per group, mean ± SEM, one-way ANOVA with Tukey's multiplex comparison test).*

In Figures 5A and B, the statistics for treatments with bilirubin versus IL4/13 are missing; otherwise, the graphs should be separated. Furthermore, line 302 on page 14, Fig. 5G does not correspond to the figure where panel

5G is missing but panel 5E is present. In this panel, CD8 control is missing to make comparisons. Line 307-308, rewrite the sentence because it is not clear.

We thank the reviewer for pointing out these inconsistencies. We have appropriately edited the manuscript text and added the requested statistics to Figure 5A and B.

To ensure that our CD8a antibody was specific, we conducted a similar T cell activation assay to the experiments presented in Figure 5 and included an isotype control. When the isotype control was compared to CD8a staining in CD8⁺ splenocytes activated with CD3/CD28 + IL-2, there was little to no non-specific CD8a staining. We also included additional controls such as splenocytes prior to sorting for CD8-positivity (Presort) and CD8⁺ solenocytes that were only activated with IL-2 or those only activated with CD3/CD28 (the same controls that were used for the assays shown in Figure 5). This new data was added to the manuscript:

Lines 294-295: An isotype control antibody was used in a similar T cell activation assay to ensure that the detected CD8a staining was specific (Supp Fig 11B).

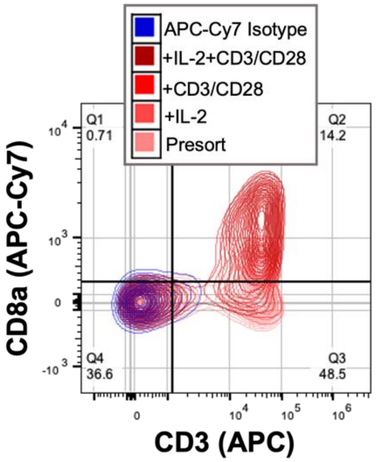

Supp Figure 11B. *CD8⁺ splenocytes stained with CellTrace Blue and treated with T cell stimulating factors (CD28/CD3 beads and IL-2). After 72 hours co-culture, expression of CD8a (APC-Cy7) was compared between splenocytes stained with an APC-Cy7 isotype control antibody (blue) versus the full T cell activation panel (red).*

In Supp Figure 10 the y-axis of the graph shows CD14 positive, so correct the graph title with PBMCs number. Furthermore, HLA-DR, a marker used in combination with other markers to distinguish between different types of macrophages, is expressed by M1. In this case, bilirubin, which should create an immunosuppressive microenvironment, promotes the expression of an M1 marker. How do you explain this?

The graph title for Supp Figure 10 has been edited. We agree with the reviewer that the increase in MHC-II expression at 2.5 µM bilirubin treatment was unexpected. Current work in the lab is further assessing the impact of bilirubin on MHC-I and MHC-II expression using a broader range of doses (from 0-150 µM). It is possible that although we saw an increase in MHC-II MFI at a low bilirubin concentration, this phenotype will not be maintained or will be the opposite at high concentrations.

Fig. 6 and related supplementary figures:
The authors use software to evaluate survival curves (KMplotter) and state that “during treatment, they predicted a decrease in progression-free survival and overall survival in several types of cancer” (lines 311-315, page 14). This is true; in fact, HMOX1 expression is associated with decreased survival. My question is: which types of cancer were evaluated? They are not mentioned in the results, in the figure, or in the legend.

However, this sentence and this result would perhaps be more appropriate at the beginning of the article, when discussing HMOX1 in different types of breast cancer and where survival curves are already present.

> We thank the reviewer for this suggestion and have moved this data to Figure 1F. Additionally we included details on cancer type in the manuscript:
>
> > Lines 96-100: "High expression of heme catabolism genes (*HMOX1, HMOX2, BLVRA,* and *BLVRB*) during immunotherapy treatment predicted a decrease in progression free and overall survival in a cohort of patients representing diverse cancer types (bladder, esophageal, adenocarcinoma, glioblastoma, hepatocellular cancer, HNSCC, NSCLC, melanoma, NSLC, and urothelial cancer, Fig 1F)."

The sentence referring to OB24 restoring sensitivity to immunotherapy is perhaps superfluous, especially since experiments with SnMP and shRNA are then continued to prove this. Perhaps it would be better to rewrite it.

> This sentence has been removed.

Generally, in an immunosuppressive TME, in addition to M2 macrophages, Tregs are also present. The data presented indicate that Tregs increase in the experimental condition of HMOX1 knockdown (supp fig 12). How can this be explained?

> We agree that this finding was unexpected. The endpoint selected for these studies was after each treatment group reached an average tumor volume of >500mm^3. At this timepoint, the tumors from all treatment groups were actively growing and we believe that initial immune responses seen in HO-1 knockdown tumors were overcome, possibly by increased expression of Tregs that are known to compensate for a loss of suppressive macrophages (PMID: 29875321). We are currently testing the impact of timing on T cell responses in these models, including assessing earlier timepoints when anti-tumor immune responses may be elevated. These ongoing works will be included in a follow-up manuscript and are beyond the scope of the current study.

The work deals with a very recent and important topic. However, there are many points that need further investigation. The authors focused on the role of HMOX1 and its metabolites and in particular bilirubin expressed at the level of tumor cells and what effects its products have on TME cells, but it is also interesting and important understand the role of HMOX1 when expressed by immune system cells such as macrophages and T cells (Tregs and CD8+).
The results are numerous but are not listed in an orderly manner, which creates confusion during read.
Furthermore, probably due to the impressive number of results, the graphs contain some errors and are not stylistically consistent (minor points but important to revise).
However, some control experiments are missing.
The discussion is very general, also in the conclusion, and lacks some recent evidence on the subject.

We thank the reviewer for summarizing their overall impressions and comments. These concluding recommendations have been addressed above or through modifications to the discussion section including the addition of recent, relevant publications.
